# PROTAC derivatization of natural products for target identification and drug discovery: Design of evodiamine-based PROTACs as novel REXO4 degraders

**DOI:** 10.1016/j.jare.2023.10.014

**Published:** 2023-10-31

**Authors:** Shuqiang Chen, Kaijian Bi, Huixin Liang, Zhe Wu, Min Huang, Xi Chen, Guoqiang Dong, Chunquan Sheng

**Affiliations:** a The Center for Basic Research and Innovation of Medicine and Pharmacy (MOE), School of Pharmacy, Second Military Medical University (Naval Medical University), Shanghai 200433, People’s Republic of China; b State Key Laboratory of Drug Research, Shanghai Institute of Materia Medica, Chinese Academy of Sciences, University of Chinese Academy of Sciences, Shanghai 201203, People’s Republic of China; c Key Laboratory of Synthetic and Natural Functional Molecule of the Ministry of Education, College of Chemistry and Materials Science, Northwest University, Xi’an 710127, People’s Republic of China

**Keywords:** PROTAC, Natural products, Target identification, Quantitative proteomics, Antitumor activities

## Abstract

**Introduction:**

Natural products (NPs) play a crucial role in the development of therapeutic drugs. However, it is still highly challenging to identify the targets of NPs. Besides, NPs usually exert their pharmacological activities *via* acting on multiple targets or pathways, which also poses great difficulties for the target identification of NPs.

**Objectives:**

Inspired by our continuous efforts in designing drug-like protein degraders, this study introduced a successful example for the target identification and drug discovery of natural products evodiamine by employing PROTAC technology.

**Methods:**

Taking advantages of proteolysis targeting chimera (PROTAC), herein an integrated strategy combining PROTAC derivatization, quantitative proteomic analysis and binding affinity validation was developed for target identification and drug discovery of antitumor NP evodiamine.

**Results:**

In this study, both highly potent PROTACs and negative controls were designed for quantitative proteomic analysis. Furthermore, REXO4 was confirmed as a direct target of 3-fluoro-10-hydroxylevodiamine, which induced cell death through ROS. In addition, the PROTAC **13c** effectively degraded REXO4 both *in vitro* and *in vivo*, leading to potent antitumor activities and reduced toxic side effects.

**Conclusion:**

In summary, we developed an integrated strategy for the target identification and drug discovery of NPs, which was successfully applied to the PROTAC derivatization and target characterization of evodiamine. This proof-of-concept study highlighted the superiority of PROTAC technology in target identification of NPs and accelerated the process of NPs-based drug discovery, exhibiting broad application in NP-based drug development.

## Highlights

• A new strategy combining PROTAC derivatization, quantitative proteomic analysis and binding affinity validation was developed for target identification and drug discovery of natural products.
• The PROTAC-based target identitication strategy was successfully applied to characterize REXO4 as a direct target of 3-fluoro-10-hydroxylevodiamine.
• Evodiamine-inspired PROTAC **13c** showed potent antitumor activity and reduced toxic side effects through REXO4 degradation.

## Graphical abstract

An integrated platform was developed for NP-inspired PROTAC design, target identification and drug discovery. As a proof-of-concept study, this platform was applied to target characterization of 3-fluoro-10-hydroxylevodiamine, which highlighted the superiority of PROTAC technology in target identification of NPs and accelerated the process of NPs-based drug discovery.

**Figure:**
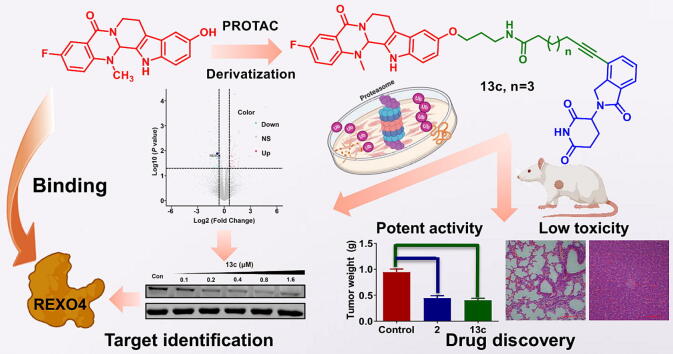

## Introduction

Natural products (NPs) have played a dominant role in the development of new drugs for the treatment of a myriad of diseases [1], [2]. Based on the analysis of the approved drugs from 1981 to 2019, more than one half of the approved small-molecule anticancer drugs were originated from NPs [3]. However, NPs generally exhibit moderate activity and possess undesirable physicochemical properties, and structural optimizations are required before they can be developed into therapeutic agents [4]. More importantly, the molecular targets of most NPs remain unclear, and elucidation of the binding targets of NPs are still highly challenging due to limited analytical approaches [5], [6], [7]. In addition, a number of NPs exert pharmacological activities *via* acting on multiple targets or pathways [8], which also poses great difficulties for the target identification of NPs.

Proteolysis targeting chimera (PROTAC) represents a promising therapeutic strategy, which degrades the disease-related proteins by employing endogenic proteasomes [9], [10], [11]. A heterobifunctional PROTAC molecule consists of three subunits: a ligand binding to the protein of interest (POI), a ligand targeting the E3 ubiquitin ligase and a chemical linker used to conjugate these two ligands [12], [13]. The POI ligand of the chimeric molecule could recognize and bind to the target protein, meanwhile the E3 ligand recruits E3 ubiquitin ligase to elicit POI poly-ubiquitination, following by the formation of the POI-PROTAC-E3 ligase ternary complex and degradation of POI through the ubiquitin–proteasome system (UPS) [14].

**Fig. 1:**
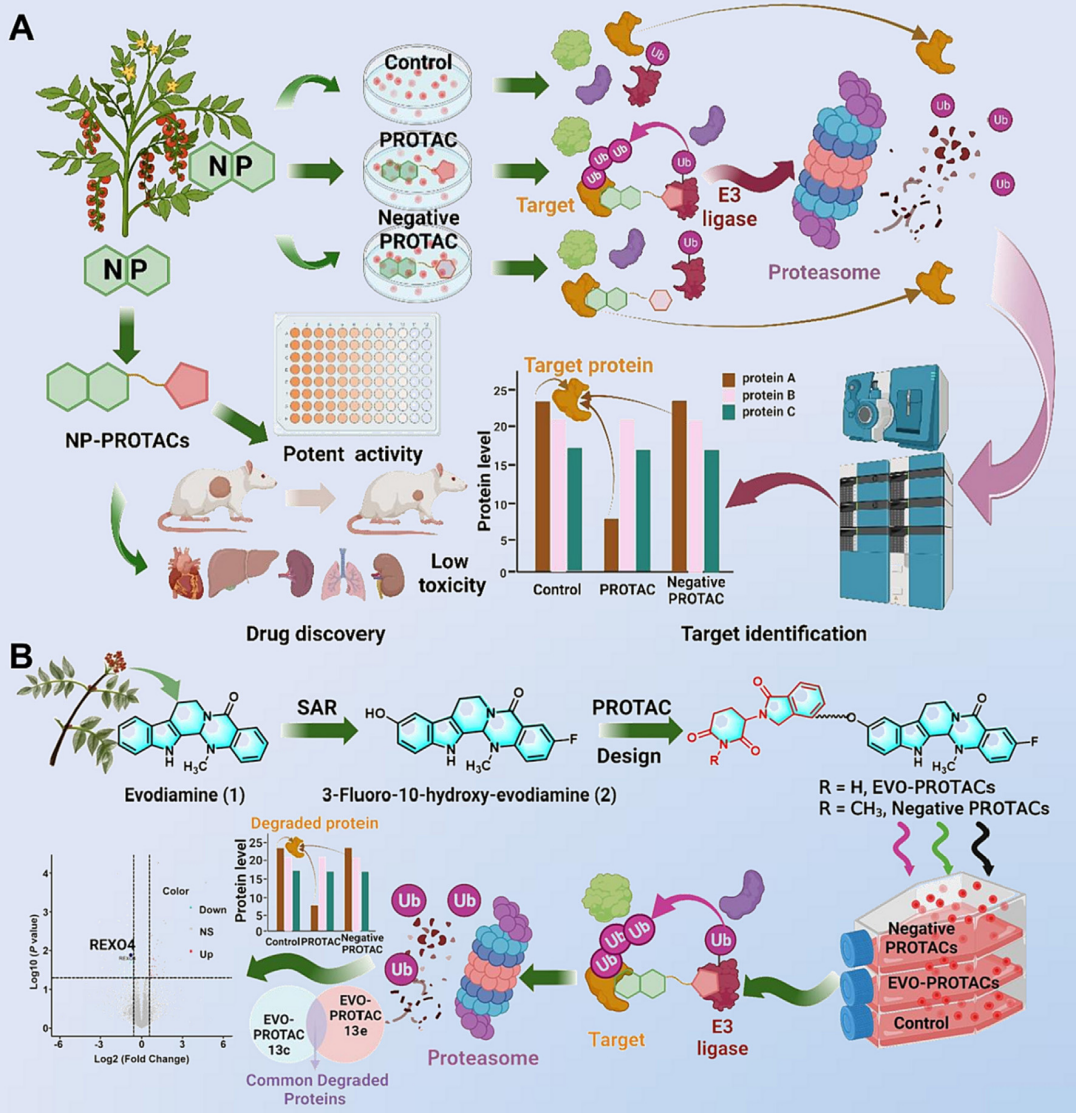
The PROTAC strategy for the target identification and drug discovery of NPs. (A) Schematic diagram of the integrated strategy combining PROTAC derivatization, quantitative proteomic analysis and binding affinity validation; (B) The application of the strategy to the target identification of the evodiamine derivative. The figure was prepared with BioRender.com.

NPs have been widely used as POI warheads or E3 ligase ligands in the design of PROTAC molecules [15]. However, successful examples using PROTAC to identify the molecular targets of NPs are still rather rare [16]. The bottlenecks mainly include: (1) the design of highly active PROTACs when the target information of NPs is unknown; (2) the identification and validation of potential targets. Herein, we developed an integrated platform for NP-inspired PROTAC design, target identification and drug discovery (Fig. 1**A**). As a proof-of-concept study, this platform was applied to target identification of antitumor NP evodiamine (compound **1** in Fig. 1**B**).

Evodiamine, an alkaloid ingredient obtained from the fruit of *Evodia rutaecarpa*, has been reported to possess multiple biological effects [17], [18]. In recent years, the antitumor effect of evodiamine has gained increasing attention and a series of highly active evodiamine derivatives were discovered by our group and others [19], [20], [21], [22], [23], [24], [25], [26], [27], [28], [29], [30]. Furthermore, our previous study indicated that 3-fluoro-10-hydroxylevodiamine (compound **2** in Fig. 1**B**) exhibited excellent antitumor activity against a variety of cancer cell lines [19]. Despite of these successes, the clinical development of evodiamine derivatives was impeded due to the limited information of molecular targets. Therefore, it is urgent to develop new technologies to accelerate the drug development process of evodiamine derivatives.

Recently, important progress has been made in NP-based PROTAC drug discovery [31], [32], [33], [34], [35]. Herein, an integrated strategy combining PROTAC derivatization, quantitative proteomic analysis and binding affinity validation was developed to identify the target of NPs. Inspired by our previous efforts in the elucidation of structure–activity relationship (SAR) of evodiamine [19], [20], [21], [36], [37] and the success on the rational design of PROTAC molecules [38], [39], [40], [41], [42], [43], highly active evodiamine-inspired PROTACs were successfully designed and RNA exonuclease 4 (REXO4) was identified to be the direct molecular target (Fig. 1**B**). The REXO4 PROTAC degraders exerted excellent antitumor activities with decreased toxic side effects. Thus, this study demonstrated that the PROTAC derivatization of NPs in combination with quantitative proteomic analysis provided a promising strategy for the target identification and drug discovery of NPs.

## Materials and methods

### Materials

Unless otherwise mentioned, all reagents and solvents were commercially available and employed without further purification. The known compounds were prepared according to the reported procedures. Colorectal carcinoma (HCT116), lung carcinoma (A549) and breast cancer (MCF-7) were obtained from Cell Bank/Stem Cell Bank, Chinese Academy of Sciences. HCT116 cells, A549 cells and MCF-7 cells were maintained in McCoy’s 5A medium, F-12K medium and MEM medium with NEAA supplemented with 10 % FBS, penicillin G (100 U/mL) and streptomycin (100 mg/mL). The cells were incubated in a humidified incubator containing 5 % CO_2_ at 37 °C. All animal studies were approved by the Committee on the Ethics of Medicine of the Second Military Medical University, which abided by the international guidelines. The female BALB/c nude mice (5 weeks old) were purchased from Chang Zhou Cavens Laboratory Animal Ltd and housed under specific pathogen-free conditions.

### Ethics statement

All experiments involving animals were conducted according to the ethical policies and procedures approved by the Committee on Ethics of Medicine, Navy Medical University (Shanghai, China) (approval number: NMU20220071).

### Synthesis procedures

**3-Fluoro-14-methyl-5-oxo-5,7,8,13,13b,14-hexahydroindolo[2′,3′:3,4]pyrido[2,1-b]quinazolin-10-yl 6-(2-(2,6-dioxopiperidin-3-yl)-1-oxoisoindolin-4-yl)hex-5-ynoate (6a)**. Intermediate **5a** (51 mg, 0.14 mmol) and compound **2** (48 mg, 0.14 mmol) were dissolved in DCM (5 mL), followed by addition of DMAP (19 mg, 0.14 mmol) and EDCI (138 mg, 0.96 mmol), and the mixture was reacted for 6 h. The final product (pale yellow solid) was afforded *via* purification by silica gel and C18 column chromatography (60 mg, 62 %). ^1^H NMR (600 MHz, DMSO‑*d*_6_): *δ* 11.30 (s, 1H), 10.98 (s, 1H), 7.72 (d, *J* = 7.6 Hz, 1H), 7.67 (d, *J* = 7.5 Hz, 1H), 7.57–7.48 (m, 2H), 7.42–7.36 (m, 1H), 7.34 (dd, *J* = 8.6, 1.7 Hz, 1H), 7.25–7.17 (m, 2H), 6.86 (d, *J* = 8.7 Hz, 1H), 6.09 (s, 1H), 5.16–5.06 (m, 1H), 4.60 (dd, *J* = 12.6, 2.8 Hz, 1H), 4.47 (dd, *J* = 17.7, 2.7 Hz, 1H), 4.37–4.30 (m, 1H), 3.24–3.15 (m, 1H), 2.92–2.83 (m, 1H), 2.82–2.72 (m, 4H), 2.68 (s, 3H), 2.64 (t, *J* = 6.9 Hz, 2H), 2.55–2.53 (m, 1H), 2.43–2.31 (m, 1H), 2.00–1.92 (m, 3H). ^13^C NMR (150 MHz, DMSO‑*d*_6_): *δ* 172.76, 172.06, 170.91, 167.62, 163.01, 157.17 (d, *J* = 238.7 Hz), 145.99, 143.85, 143.61, 134.36, 134.15, 131.98, 131.11, 128.59, 125.83, 122.73, 122.09 (d, *J* = 7.1 Hz), 121.81 (d, *J* = 7.2 Hz), 120.59 (d, *J* = 23.0 Hz), 118.65, 116.32, 113.38 (d, *J* = 23.5 Hz), 111.96, 111.84, 110.60, 95.31, 77.01, 69.16, 51.60, 46.97, 40.27, 36.70, 32.64, 31.13, 23.51, 22.29, 19.45, 18.27. HR-ESI-MS calcd for C_38_H_32_FN_5_O_6_ [M + H]^+^ 674.2409, found 674.2414.

The synthetic methods of target compounds **6b-6e**, **9a-9b** refered to compound **6a**.

**6-(2-(2,6-Dioxopiperidin-3-yl)-1-oxoisoindolin-4-yl)-*N*-(3-((3-fluoro-14-methyl-5-oxo-5,7,8,13,13b,14-hexahydroindolo[2′,3′:3,4]pyrido[2,1-b]quinazolin-10-yl)oxy)propyl)hex-5-ynamide (13a).** Intermediate **11** (78 mg, 0.16 mmol) was dissolved in DCM (3 mL) and TFA (1 mL) was dropwise added. 1 h later, the solvent was evaporated under reduced pressure and the residue was redissolved in dry DMF (2 mL), followed by addition of intermediate **5a** (56 mg, 0.16 mmol), HATU (180 mg, 0.48 mmol) and DIPEA (82 mg, 0.63 mmol). The reaction mixture was stirred for 5 h, followed by EA extraction, concentration and purification by silica gel and C18 column chromatography to give pale yellow solid (62 mg, 55 %). ^1^H NMR (600 MHz, DMSO‑*d*_6_): *δ* 10.99 (s, 2H), 7.95 (t, *J* = 5.5 Hz, 1H), 7.70 (d, *J* = 7.6 Hz, 1H), 7.63 (d, *J* = 7.6 Hz, 1H), 7.55–7.48 (m, 2H), 7.41–7.35 (m, 1H), 7.24 (d, *J* = 8.8 Hz, 1H), 7.17 (dd, *J* = 8.9, 4.6 Hz, 1H), 6.98 (d, *J* = 2.4 Hz, 1H), 6.76 (dd, *J* = 8.8, 2.4 Hz, 1H), 6.06 (s, 1H), 5.12 (dd, *J* = 13.3, 5.2 Hz, 1H), 4.64–4.56 (m, 1H), 4.45 (d, *J* = 17.7 Hz, 1H), 4.31 (d, *J* = 17.6 Hz, 1H), 3.97 (t, *J* = 6.3 Hz, 2H), 3.25–3.14 (m, 3H), 2.95–2.86 (m, 1H), 2.86–2.75 (m, 2H), 2.67 (s, 3H), 2.62–2.55 (m, 1H), 2.48 (t, *J* = 7.0 Hz, 2H), 2.46–2.40 (m, 1H), 2.26 (t, *J* = 7.4 Hz, 2H), 2.03–1.95 (m, 1H), 1.87–1.83 (m, 2H), 1.83–1.77 (m, 2H). ^13^C NMR (150 MHz, DMSO‑*d*_6_): *δ* 172.82, 171.41, 170.93, 167.65, 163.03, 157.07 (d, *J* = 238.6 Hz), 152.56, 145.97, 143.78, 134.10, 131.95, 131.75, 130.24, 128.53, 126.11, 122.61, 121.93 (d, *J* = 7.1 Hz), 121.54 (d, *J* = 7.5 Hz), 120.55 (d, *J* = 23.0 Hz), 118.77, 113.36 (d, *J* = 23.7 Hz), 112.58, 112.28, 111.36, 101.32, 95.79, 76.64, 69.30, 65.82, 51.64, 46.99, 40.43, 36.57, 35.64, 34.23, 31.18, 29.14, 24.33, 22.32, 19.60, 18.45. HR-ESI-MS calcd for C_41_H_39_FN_6_O_6_ [M + H]^+^ 731.2988, found 731.2985.

The synthetic methods of target compounds **13b-13e**, **14a-14b** refered to compound **13a**.

### *In vitro* cytotoxicity assay

The CCK-8 method was adopted to determine the *in vitro* antiproliferative activities of the final compounds. HCT116, A549, MCF-7 cells (5 × 10^3^ cells/well) were seeded in 96-well plates and cultured in 5 % CO_2_ at 37 °C for another 24 h. Subsequently, the target compounds were added to wells in triplicate at various concentrations and incubated for 72 h. Then, 10 μL CCK-8 solution was added into every well and the plate was incubated for another appropriate time. Finally, the Biotek Synergy H2 reader was used to measure the absorbance (OD) at 450 nm. The IC_50_ values (half maximal inhibitory concentration) were calculated by GraphPad Prism 5.0.

### Mass spectrometry for proteomics

HCT116 cells were seeded in T25 culture dishes at a density of 1 × 10^6^ cells/mL and cultured overnight. Then 100 nM of the test compounds were added and co-incubated for additional 24 h. Then the cells were collected and washed with PBS for 3 times, followed by a proteomic analysis implemented by the Proteomics Platform of Core Facility of Basic Medical Science, Shanghai Jiao Tong University School of Medicine (STJU-SM, Shanghai, China).

### Western blotting analysis

HCT116 cells (4 × 10^5^ cells/well) were seeded in six-well plates and cultured overnight. And then, the cells were co-incubated with diverse concentrations of the test compounds for another 24 h. Subsequently, the cells were collected and washed with cold PBS. Cells were then lysed with RIPA buffer containing phosphatase inhibitor (EpiZyme #GRF102) and protease inhibitors (EpiZyme #GRF101) on ice to isolate the total protein, and the cell lysis solution was centrifuged at 12000 rpm at 4 °C for 15 min. The cell supernatant was collected and measured the protein concentration with the BCA kit. The same amounts of protein (30 μg) were separated by SDS-PAGE gels, and then the protein was transferred to polyvinylidene fluoride membranes. 5 % BSA was then used to block the membrane for 2 h at room temperature. Subsequently, primary antibodies against REXO4 (Proteintech, 18890-1-AP, 1:1000), γH_2_AX (Abcam # ab2893, 1:1000) and anti-GAPDH (Abcam #ab181602, 1:10,000) were incubated with various membranes overnight at 4 °C. The fluorescent secondary antibodies were then used to incubate with them for 2 h after three times washing with TBST, followed by the analysis using a LI-COR Odyssey imaging system.

### Detection of intracellular ROS

The fluorescent probe 2′,7′-dichloro-fluorescein diacetate (DCFH-DA, Beyotime, China) was employed to determine the intracellular ROS. HCT116 cells (2 × 10^5^ cells/well) were seeded into 6-well plates and cultured overnight before the incubation of various concentrations of the test compounds for 48 h. The cells were then treated with DCFH-DA (10 μM) in serum-free medium for another 30 min. Finally, the collected cells were resuspended with 50 μL of PBS and analyzed by flow cytometry (BD Accuri C6).

### Expression and purification of REXO4

The GST-REXO4-pET28a recombinant plasmid was prepared by Wuxi Biortus Biosciences Co. Ltd. Subsequently, the recombinant plasmid was transformed into *E. coli* strain BL21 (DE3), which was cultured at 37 °C until OD_600_ = 0.6 and was induced by 0.1 mM IPTG for 18–20 h at 18 °C. The cells were collected by centrifugation at 6000 rpm at 4 °C for 15 min, and then resuspended in lysis buffer (50 mM NaH_2_PO_4_, 20 mM imidazole, 500 mM NaCl, pH 8.0), and then dissociated by sonication. The cell debris were removed by centrifugation (4 °C, 13000 rpm, 45 min) and the supernatant was filtered. And then the supernatant was loaded onto HisTrap HP column (GE Healthcare) preequilibrated with lysis buffer containing 20 mM imidazole. The protein was eluted by 50 mM NaH_2_PO_4_, pH 8.0, 500 mM NaCl, with the gradient of imidazole between 20 and 250 mM. The elution was tested by SDS-PAGE, collected based on purity, concentrated and loaded onto Superdex 200 10/300 GL(GE Healthcare) equilibrated with 20 mM HEPES, 150 mM NaCl, 1 mM EDTA, pH 7.0. Eluted protein corresponding to the major peak was collected and concentrated to 11 mg/mL, and then was flash frozen by using liquid nitrogen and preserved at −80 °C.

### Microscale thermophoresis (MST) assay

The purified REXO4 protein was labeled in accordance with the protocol of Protein labelling kit RED-NHS (Nanotemper, cat. no. L001). The labeled REXO4 was then attenuated to 200 nM with the assay buffer containing 20 mM HEPES (pH 7.4) and 5 % (v/v) DMSO. The test compound was diluted to various concentrations and incubated with REXO4 protein at room temperature for 15 min. Subsequently, the samples were loaded into Monolith standard-treated capillaries and the thermophoresis was proceeded at 25 °C on a Monolith NT.115 instrument (Nano Temper Technologies). The experiment data were acquired with 100 % light-emitting diode (LED) power. The dissociation constant *K*_D_ values were calculated by the NT Analysis software (Nano Temper Technologies).

### Real-time PCR

HCT116 cells (2 × 10^5^ cells/well) were seeded in six-well plates and cultured overnight. Subsequently, compound **2** was added to co-incubate with HCT116 cells for another 24 h. After that, TRIzol reagent (ambion) was used to extract the total RNA. The cDNA was reverse transcribed by using the cDNA synthesis kit (TaKaRa, 6210A). A real-time PCR (20 μL) experiment was conducted with deionized water (7.2 μL), forward primers (0.4 μL), 2 × SYBR Green Master (10 μL), cDNA solution (2 μL) and reverse primers (0.4 μL) on a QuantStudioTM 3 Real-Time PCR apparatus. Finally, the RNA expression levels of REXO4 were calculated using the 2^-ΔΔCT^ method. GAPDH was used for normalization.

REXO4 (5′ to 3′) forward primers: TCTCTTCCGGAGTCTTTTCCTG.

REXO4 (5′ to 3′) reverse primer: CCTTCACTTGAGGCGAGGTC.

GAPDH (5′ to 3′) forward primers: CATGAGAAGTATGACAACAGCCT.

GAPDH (5′ to 3′) reverse primer: AGTCCTTCCACGATACCAAAGT.

### Cell apoptosis detection

HCT116 cells (2 × 10^5^ cells/well) were seeded in six-well plates and cultured overnight. Subsequently, various concentrations of the test compounds were added and co-treated for 48 h. And then the HCT116 cells were collected, washed and centrifuged at 1000 rpm for 5 min. After that, the sediment was resuspended in 300 μL of 1 × assay buffer, and then the mixture of Annexin V-FITC (5 μL) and PI (10 μL) were added for an additional incubation (15 min) at room temperature in the dark. The samples were then determined by flow cytometry (BD Accuri C6).

### Statistical analysis

An unpaired *t* test was performed when comparing two different groups, and one-way ANOVA was used to compare the multiple group in this study. The calculated values are presented as the mean ± standard deviation (SD).

## Results

### Design and chemical synthesis of evodiamine-based PROTACs

The PROTAC technology has the advantage of continuous cellular effect and only sub-stoichiometric amount of PROTAC molecules is required to trigger target degradation [39]. Therefore, the protein degradation effect might occur at a lower concentration than that required for protein inhibition by traditional small molecule inhibitors, resulting in a higher activity and fewer side effects. Due to these distinct advantages [44], we envisioned that the PROTAC derivatization of NPs might provide a new strategy to elucidate the molecular targets of NPs and improve their pharmacological activities simultaneously.

The first step of NP-based PROTAC design is to select a template molecule. In this study, evodiamine derivative **2** (see Fig. 1**B**) was selected as the POI ligand for PROTAC design due to high antitumor activity, convenient chemical synthesis and well-established SARs [20]. The 10-hydroxyl group in compound **2** provides a suitable site for introducing the linker and E3 ligase ligand, because derivatization at the 10-hydroxyl group had little impact on the antitumor activity [20]. Lenalidomide was selected as the E3 ligase cereblon (CRBN) ligand, which was conjugated with the POI ligand through various length of alkyl linkers. To ensure the desired chemical stability, both ester and ether linkers were used. As a result, a series of PROTAC molecules (herein referred to EVO-PROTACs) were successfully designed. To improve the efficiency and accuracy of quantitative proteomics, negative controls were designed by the methylation of the glutarimide amino group (compounds **9a-b** and **14a-b**) to block the binding with CRBN ligase.

**Scheme 1:**
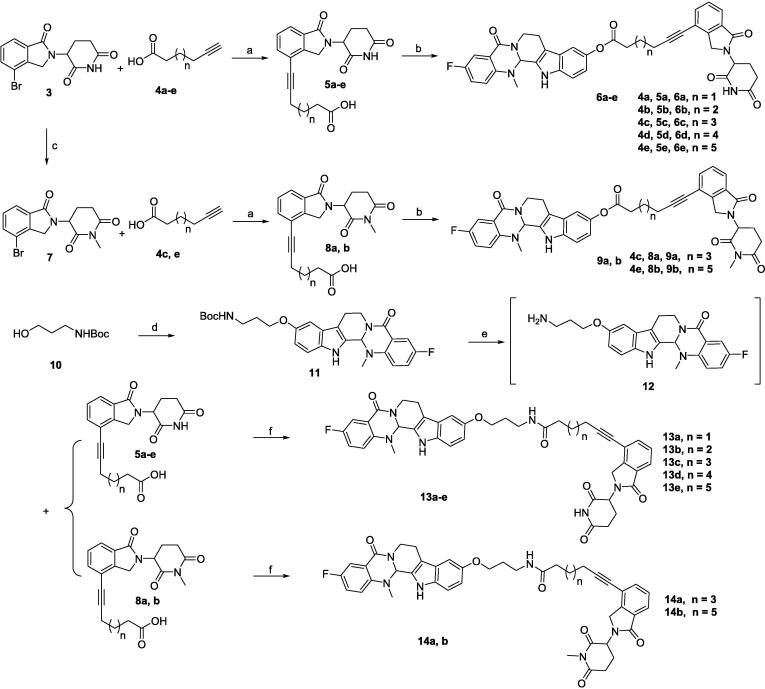
Reagents and Conditions: (a) compound **2**, Pd(PPh_3_)Cl_2_, CuI, TEA, DMF, 80 °C, 5 h, 74–83 %; (b) DMAP, EDCI, DCM, 6 h, 61–69 %, (c) NaH, MeI, 0 °C to rt, 4 h, 78 %. (d) compound **2**, PPh_3_, DIAD, THF, 0 °C to rt, 5 h, 82 %; (e) TFA, DCM, rt, 1 h; (f) HATU, DIPEA, DMF, 5 h, 48–56 % over 2 steps.

EVO-PROTACs were synthesized according to the synthetic routes depicted in Scheme 1. Evodiamine derivative **2** was synthesized referring to the reported procedure [36]. Lenalidomide derivative (compound **3**) was reacted with various alkynoic acids by Sonogashia coupling reaction to obtain key intermediates **5a-e**, followed by a condensation reaction with compound **2** catalyzed by 4-dimethylaminopyridine (DMAP) and 1-ethyl-3-(3-dimethylaminopropyl) carbodiimide hydrochloride (EDCI) to afford the target compounds **6a-e.** Meanwhile, negative controls **9a** and **9b** were prepared *via* a similar condensation reaction.

For the synthesis of EVO-PROTACs with ether linkers, 3-(Boc-amino)-1-propanol (compound **10**) was reacted with compound **2** by the Mitsunobu reaction to obtain intermediate **11**, and then the di-*tert*-butyl dicarbonate (Boc) was removed in the presence of trifluoroacetic acid (TFA) to give compound **12**, which was used directly in the next step without further purification. Subsequently, the target compounds **13a-e** were acquired by a condensation reaction of compound **12** with intermediates **5a-e** in the presence of *N*,*N*-diisopropylethylamine (DIPEA) and 2-(7-Azabenzotriazol-1-yl)-*N*,*N*,*N*',*N*'-tetramethyluronium hexafluorophosphate (HATU). Similarly, negative controls **14a** and **14b** containing an ether linker were also prepared.

### *In vitro* antiproliferative activities of EVO-PROTACs against HCT116 cells

Initially, the *in vitro* antiproliferative activities of the target compounds against human colon cancer HCT116 cells were evaluated by using cell counting kit-8 (CCK-8) assay. 3-Fluoro-10-hydroxylevodiamine was used as the positive control. As demonstrated in Table 1, all the EVO-PROTACs exhibited moderate to excellent antitumor activities against HCT116 cells. Specifically, target compounds **6a-6e** with the ester linkers exhibited slightly better or comparable antiproliferative activity compared with parent compound **2**. For the target compounds tethered by an ether bond, the antiproliferative activities was increased with the extension of the linker. The IC_50_ value of compounds **13c** and **13e** was 0.019 μM and 0.021 μM, respectively, which was comparable to parent compound **2** (IC_50_ = 0.015 μM).

**Table 1:** *In vitro* antitumor activities (IC_50_, μM, 72 h) of the EVO-PROTACs.

Compounds	HCT116	A549	MCF-7
**6a**	0.011 ± 0.0090	0.37 ± 0.0050	0.075 ± 0.016
**6b**	0.007 ± 0.0010	0.032 ± 0.00020	0.040 ± 0.0039
**6c**	0.034 ± 0.0050	0.030 ± 0.0020	0.060 ± 0.0057
**6d**	0.029 ± 0.0020	0.035 ± 0.0030	0.031 ± 0.00050
**6e**	0.029 ± 0.0010	0.045 ± 0.029	0.039 ± 0.0053
**13a**	8.7 ± 0.87	>10	>10
**13b**	4.8 ± 0.81	2.9 ± 0.29	3.2 ± 0.43
**13c**	0.019 ± 0.0020	0.12 ± 0.015	0.045 ± 0.0064
**13d**	0.019 ± 0.0010	0.082 ± 0.0090	0.021 ± 0.0011
**13e**	0.021 ± 0.0060	0.096 ± 0.0090	0.014 ± 0.00080
**3**	>20	NT[a]	NT
**2**	0.015 ± 0.0040	0.050 ± 0.0017	0.068 ± 0.0067

[a] Not Tested.

In order to further determine the antitumor spectrum of EVO-PROTACs, human lung cancer (A549) and human breast cancer (MCF-7) cell lines were selected to assay the antiproliferative activities. In accordance with the antitumor activities against HCT116 cells, all the target compounds also demonstrated moderate to excellent antitumor activities against MCF-7 and A549 cells (Table 1). For instance, compounds **13c-e** exhibited superior antitumor activities than parent compound **2**. Especially, compound **13e** (IC_50_ = 0.014 μM) were approximately 5 times more potent than parent compound **2** against the MCF-7 cell line (IC_50_ = 0.068 μM). These results confirmed that the antitumor activity was retained or improved for the EVO-PROTACs, providing potent probes for the subsequent target validation explorations.

To further investigate the stability of EVO-PROTACs, compounds **6a** and **13c** were selected to perform the stability test. The results revealed that the hydrolysis ratio of compound **6a** was 23.5 % after the incubation with HCT116 cells for 72 h (Fig. S1A in Supporting Information). In contrast, ether compound **13c** showed high stability because compound **2** was not detected after the incubation of EVO-PROTAC **13c** with HCT116 cells for 72 h (Fig. S1B in Supporting Information).

### Rational design of the integrated platform for PROTAC-based target identification

**Fig. 2:**
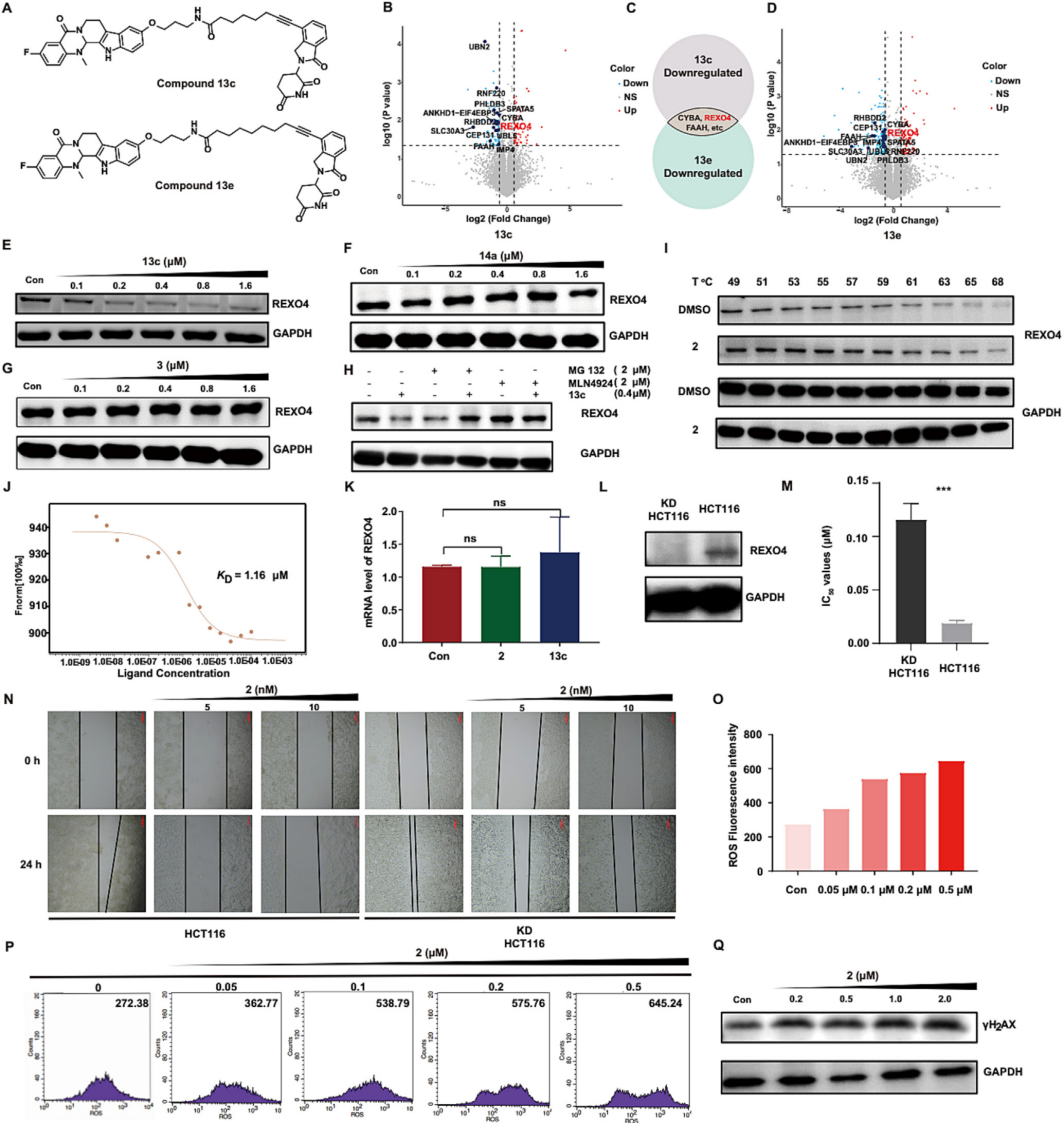
REXO4 is a target of 3-fluoro-10-hydroxylevodiamine. (A) The chemical structures of EVO-PROTAC **13c** and **13e**. The up-regulated (red) and down-regulated (light blue) proteins whose fold change (EVO-PROTAC/negative control) exceed the value of 1.5 (*P* < 0.05) after the treatment with EVO-PROTAC **13c** (B) and **13e** (D). (C) The partial common proteins degraded by EVO-PROTACs by taking an intersection. The expression level of REXO4 protein in HCT116 cells after the treatment of EVO-PROTAC **13c** (E), negative control **14a** (F) and thalidomide (G) for 24 h. (H) The expression level of REXO4 protein after the pretreatment with proteasome inhibitor MG132 (2 μM) or the neddylation inhibitor MLN4924 (2 μM) for 2 h before the incubation with either vehicle (DMSO) or EVO-PROTAC **13c** (0.4 μM) for 24 h. (I) Western blot analysis of REXO4 protein levels in the presence of 3-fluoro-10-hydroxylevodiamine treatment for 4 h and subsequently heated at different temperatures (49–68 °C). (J) MST assay between REXO4 and 3-fluoro-10-hydroxylevodiamine. (K) Quantitative real-time PCR analysis of the mRNA level of REXO4 after 24 h of treatment with either vehicle (DMSO), 3-fluoro-10-hydroxylevodiamine (0.8 μM) or EVO-PROTAC **13c** (0.8 μM) in HCT116 cells. Data were normalized with GAPDH. (L) The expression level of REXO4 protein in HCT116 cells and REXO4 KD HCT116 cells. (M and N) The IC_50_ values and wound heal inhibition of 3-fluoro-10-hydroxylevodiamine against HCT116 cells and REXO4 KD HCT116 cells. (O and P) The concentration-dependent increase of ROS level after 24 h of treatment with 3-fluoro-10-hydroxylevodiamine at various concentration. (Q) The expression level of γ-H_2_AX protein after the treatment with 3-fluoro-10-hydroxylevodiamine for 24 h.

In consideration of the stability and antitumor activities of EVO-PROTACs, PROTACs **13c** and **13e** (see Fig. 2**A**) and their negative controls **14a** and **14b** were selected for further investigations. PROTACs selectively degraded target proteins and the decreased protein expression level could be determined by the quantitative proteomics analysis. Therefore, the PROTAC technology could be adopted to identify potential targets of the NPs. HCT116 cells were cultured in the presence or absence of EVO-PROTACs (compounds **13c** and **13e**) and negative controls (compounds **14a** and **14b**) for a certain time, respectively. Subsequently, HCT116 cells were harvested and analyzed by a data independent acquisition (DIA)-based quantitative proteomic approach. As illustrated in Fig. 2**C**, the proteins downregulated by compound **13c** but not by compound **14a** were selected. To obtain reliable results, similar procedure was also applied to obtain the selectively degraded proteins induced by compound **13e** but not by compound **14b**. Finally, the common proteins degraded by the two EVO-PROTACs were further identified by taking their intersection.

### Evo-protacs induced the protein degradation of REXO4 via the UPS

Preliminary data analysis revealed that more than 9000 quantifiable proteins were detected in this experiment. Among them, several proteins could be degraded by EVO-PROTACs with significant differences (*p* < 0.05) in protein levels when the fold change value was set as 1.5 fold (Fig. 2**B–D**). Further bioinformatic analysis of the results indicated that REXO4 could be degraded by EVO-PROTACs but not by the negative controls. In order to further validate that the REXO4 protein degradation was induced by EVO-PROTACs, compound **13c** was subjected for the western blotting analysis. As illustrated in Fig. 2**E**, REXO4 was effectively degraded by EVO-PROTAC **13c** in HCT116 cells at a dose-dependent manner. The maximum degradation efficiency (D_max_ = 88.3 %) was observed at the concentration of 0.8 μM. Nevertheless, negative control EVO-PROTAC **14a** (Fig. 2**F**) and CRBN ligand thalidomide (Fig. 2**G**) were unable to decrease the expression level of REXO4 in HCT116 cells. These results indicated that EVO-PROTACs could selectively induce the degradation of REXO4. Furthermore, the western blotting analysis was performed after the pretreatment with proteasome inhibitor MG132 or the neddylation inhibitor MLN4924 to determine whether EVO-PROTAC degraded REXO4 protein through the UPS. As shown in Fig. 2**H**, both MG132 and MLN4924 blocked the degradation effect, confirming that the degradation of REXO4 induced by EVO-PROTAC **13c** relied on the UPS. Similarly, PROTAC **13e** also effectively degraded REXO4 *via* the UPS mechanism (Fig. S2 in Supporting Information).

### Validation of REXO4 as a direct target of 3-Fluoro-10-hydroxylevodiamine

Furthermore, to confirm the direct binding between compound **2** and REXO4, cellular thermal shift assay (CETSA) was carried out. As exhibited in Fig. 2**I**, compound **2** increased the cellular thermal stability of REXO4 when the temperature was higher than 53 °C. Moreover, the direct binding affinity between compound **2** and REXO4 was also assessed by the microscale thermophoresis (MST) assay. The results indicated that compound **2** could bind to REXO4 with an equilibrium dissociation constant (*K*_D_) value of 1.16 μM (Fig. 2**J**). In addition, the quantitative real-time PCR was also used to explore whether EVO-PROTAC downregulated the expression level of REXO4 at the transcriptional level. The results indicated that EVO-PROTAC **13c** and compound **2** had little effect to induce significant changes in REXO4 mRNA level (Fig. 2**K**). Based on these results, REXO4 could be confirmed to be a direct target of evodiamine derivative **2**. In order to further investigate whether REXO4 is a key determinant of antitumor activity, the REXO4 KD HCT116 cell line (knockdown of REXO4 gene) were prepared (Fig. 2**L**), which was employed to conduct the cell viability and migration assays. As demonstrated in Fig. 2**M**, compound **2** exhibited lower inhibitory activity against KD HCT116 cells compared with that against normal HCT116 cells, with a 6.1 times elevation of the IC_50_ value. In addition, the co-incubation of HCT116 cells with compound **2** significantly inhibited the wound heal, while compound **2** only exhibited moderate inhibition on the wound heal against REXO4 KD HCT116 cells (Fig. 2**N**). These results further illustrated that compound **2** exerted antitumor effect thorugh the inhibition of REXO4.

### 3-Fluoro-10-hydroxylevodiamine induced the DNA damage by increasing the ROS

Based on the results of previous studies, REXO4 was reported to be highly correlated with neuropathic pain and familial isolated pituitary adenoma (FIPA) [45]. Whilst, the effect of the REXO4 expression level on tumor growth was seldom reported. Inspired by the result that downregulation of REXO4 protein in hepatocellular carcinoma (HCC) was beneficial to the cell multiplication inhibition [46], [47], we hypothesized that the interaction with REXO4 might be helpful for the proliferation inhibition of HCT116 cells. Moreover, REXO4 was reported to possess exonuclease domains, which were related to the transcriptional regulation of quinone reductase (QR) and the repair of DNA damage [48]. QR is a kind of antioxidative stress enzyme that prevents the production of reactive oxygen species (ROS) [49]. Therefore, using fluorescence probe 2′, 7′-dichlorodihydrofluorescein diacetate (DCFH-DA) and flow cytometry, the intracellular ROS level was evaluated after the treatment with compound **2**. As demonstrated in Fig. 2**O and 2P**, the co-incubation of HCT116 cells with compound **2** significantly elevated the ROS level in a concentration-dependent manner. The increased ROS could enhance the oxidative stress and result in the oxidative damage to DNA [50]. Hence, the expression of γ-H_2_AX, a biomarker to monitor the generation of DNA damage [51], was evaluated by the western blotting analysis. The results indicated that HCT116 cells treated with compound **2** led to increased expression level of γ-H_2_AX (Fig. 2**Q**). Collectively, compound **2** could induce the elevation of ROS and DNA damage in tumor cells by interacting with REXO4.

### EVO-PROTAC **13c** inhibited HCT116 cell Proliferation, invasion and metastasis

**Fig. 3:**
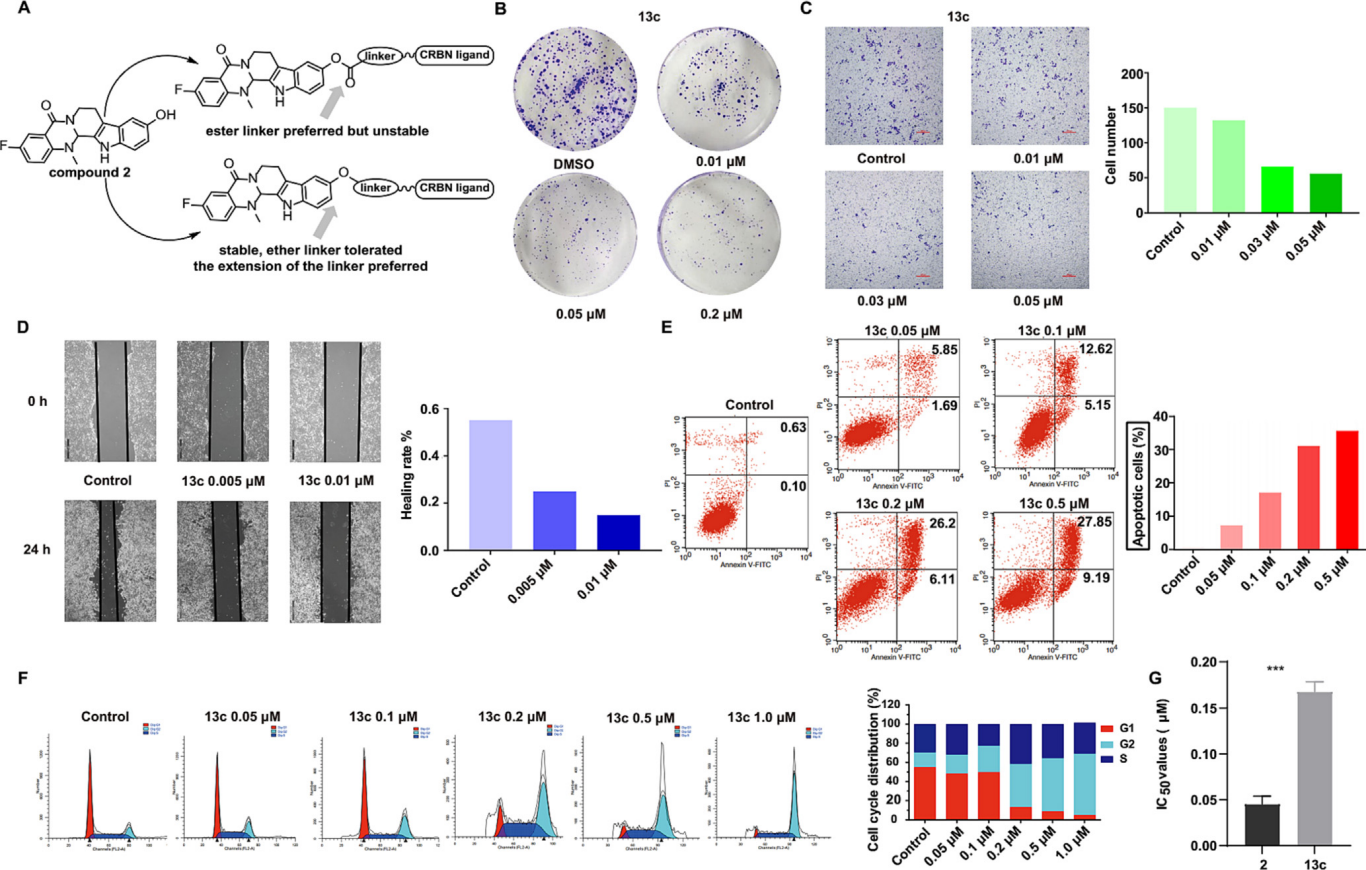
The *in vitro* antitumor activity of EVO-PROTAC **13c**. (A) A diagram summarize of the EVO-PROTAC SARs. (B) Results of clonogenicity on HCT116 cells treated with EVO-PROTAC **13c** at indicated concentrations. The migration and invasion inhibition effect of EVO-PROTAC **13c** on HCT116 cells at indicated concentrations for 24 h by transwell (C) and wound-healing assay (D). Apoptosis (E) and cell cycle arrest (F) analysis of HCT116 cells after the incubation with EVO-PROTAC **13c** for 48 h by a flow cytometer. (G) The IC_50_ values of EVO-PROTAC **13c** against human normal cell line NCM-460.

The antitumor potency of REXO4 degrader **13c** was further evaluated. Initially, the inhibitory effect of compound **13c** against colony formation was determined. As shown in Fig. 3**B**, compound **13c** dose-dependently restrained the growth and colony formation of HCT116 cells. The invasion and migration of cancer cells is highly correlated with the cancer related deaths, and it is of great significance to restrain the tumor cell metastasis [26]. Furthermore, the anti-metastasis capacity of compound **13c** was evaluated by cell invasion and migration (transwell and wound-healing) assays. Specifically, the transwell assay manifested that compound **13c** significantly decreased the number of HCT116 cells in migrating into the lower compartment (Fig. 3**C**), demonstrating efficient anti-invasion capacity against HCT116 cells. Besides, the wound-healing assay was performed and the cell images were photographed at 0 and 24 h, respectively. The wound-healing rate of the control group reached more than 55 %, while compound **13c** effectively inhibited the wound heal with the wound-healing rate of 15 % at the concentration of 0.01 μM (Fig. 3**D**). These results proved that compound **13c** could protect the normal tissues by effectively inhibiting the metastasis of tumor cells.

### EVO-PROTAC 13c induced cell apoptosis and cycle arrest at G2/M phase

Uncontrolled cell growth is one of the primary characteristics of tumor cells. The promotion of tumor cell apoptosis and regulation of cell cycle distribution contributes to the growth inhibition of tumor cells. Therefore, flow cytometric analysis was implemented to explore the influence of EVO-PROTAC **13c** on the induction of cell apoptosis and cell cycle distribution. As shown in Fig. 3**E**, after the incubation with compound **13c** at the concentrations of 0.05, 0.1, 0.2 and 0.5 μM for 48 h, the number of apoptotic cells were obviously increased to 7.54 %, 17.77 %, 32.31 % and 37.04 %, respectively, while the percentages of apoptotic cells in the control group was only 0.73 %. Furthermore, compound **13c** remarkably arrested HCT116 cell cycles at the G2/M stage in a dose-dependent manner (Fig. 3**F**). Furthermore, the cytotoxicity of compound **13c** was evaluated agaisnt human normal cell line NCM460. The results indicated that compound **13c** exhibited low cytotoxicity, Moreover, the IC_50_ of compound **13c** was 3.7 times higher than that of compound **2** against NCM460 cell lines **(**Fig. 3**G)**.

### EVO-PROTAC 13c showed potent *in vivo* anti-tumor activities with reduced toxicity

**Fig. 4:**
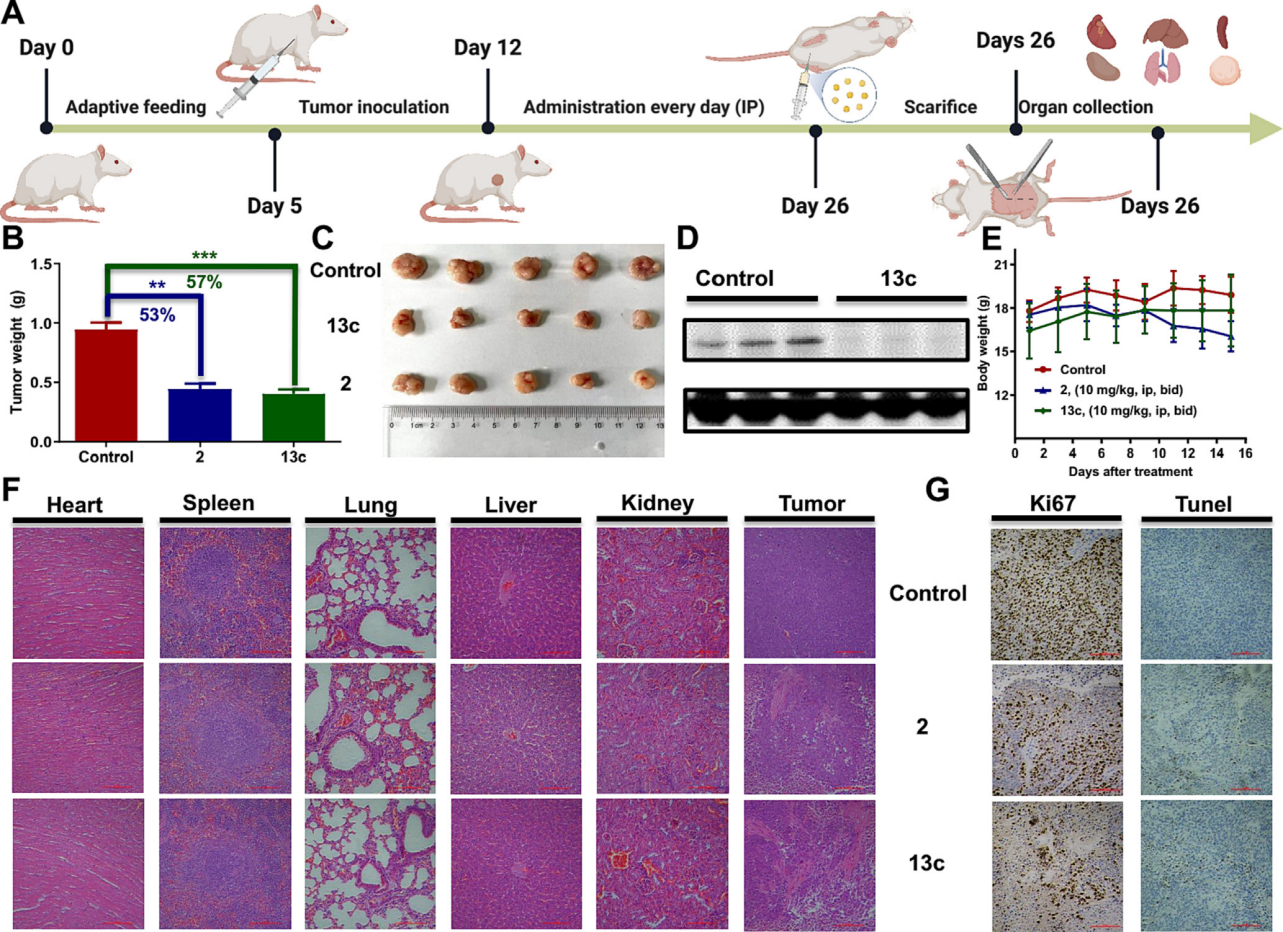
The *in vivo* antitumor activity, REXO4 degradation and toxicity of EVO-PROTAC **13c**. (A) The schematic illustration of the *in vivo* tumor-inhibition experiment and the general treatment procedure. (B) The comparison of tumor weight in each group. (C) The macroscopic views of dissected xenograft tumor tissues in each group. (D) The expression levels of REXO4 in the tumors after IP administration with EVO-PROTAC **13c**. (E) The changes in body weight during the treatment. (F) The H&E staining of major organs and the tumor after the treatment with EVO-PROTAC **13c** or compound **2**. (G) The IHC of Ki67 and Tunel staining of tumor tissues after the treatment with EVO-PROTAC **13c** or compound **2**. The figure was prepared with BioRender.com.

To further validate the therapeutic potential of the evodiamine-based PROTAC derivatives, compound **13c** was selected to explore the *in vivo* anticancer capacity against HCT116 tumor xenograft BALB/c nude mice models. After the tumor volume of the mice reached to about 100 mm^3^, compound **13c** was administered intraperitoneally (IP) at the dose of 10 mg/kg twice a day for 14 consecutive days using parent compound **2** as the control. Significant *in vivo* tumor inhibitory effect was observed for compound **13c** with tumor growth inhibition (TGI) value of 57 % (Fig. 4**B-C**), which was slightly better than parent compound **2** (TGI = 53 %). Furthermore, in order to investigate the *in vivo* degradation of REXO4 by EVO-PROTAC **13c**, the tumor was segregated after the sacrifice of the mice and extracted the total protein to perform western blotting analysis. As described in Fig. 4**D**, compound **13c** exhibited good degradation effect, indicating that compound **13c** exerted the anti-tumor efficiency through the degradation of REXO4 protein *in vivo*. Therefore, the result further convinced that REXO4 is a direct target of compound **2**. Interestingly, PROTAC **13c** showed lower toxicity than compound **2** during the animal studies. The body weight of the nude mice treated with compound **13c** was increased during the test, whereas the nude mice in the compound **2** treated group decreased visibly (Fig. 4**E,** *p* < 0.05) with one of the mice died.

Consistently, the analysis of the hematoxylin-eosin (H&E)-stained sections of major organs (heart, liver, spleen, lung and kidney) demonstrated low toxic side effects of compound **13c**. In contrast, compound **2** caused damage to several organs. Specifically, the thickened alveolar walls, atrophy of glomerulus and varied sizes of vacuoles were observed in lung, spleen and liver of the compound **2**-treated mice. The number of Ki67-stained cells in the drug-treated groups decreased remarkably, indicating that compound **13c** significantly inhibited the proliferation of tumor cells. Moreover, the number of Tunel-stained cells in the drug-treated groups elevated obviously compared with the control group, manifesting that compound **13c** inhibited the proliferation of tumor cells by the induction of tumor cell apoptosis.

## Discussion

Due to the importance of NPs in drug discovery, the development of effective methods for the identification of molecular target(s) is highly important in NPs-based drug development. Affinity-based protein profiling (ABPP), activity-based protein profiling (ABP), drug affinity responsive target stability (DARTS) and cellular thermal shift assay (CETSA) have been widely used in target identification of NPs [52], [53], [54]. However, the targets of most NPs are still unknown because of the limitations of current methods. Thus, it is highly desirable to develop new strategies for the target identifications of NPs. Taking advantages of the PROTAC technology, herein an integrated platform combining PROTAC derivatization of NPs, quantitative proteomic analysis and binding affinity validation was successfully developed.

Comparing with a recent example of PROTAC-based target identification [16], our method rationally designed the workflow to improve the efficiency of the target identification. From rather limited examples, degraders after PROTAC derivatization of NPs are generally active at micromolar concentrations [16], which is insufficient for target identification. Highly active PROTACs are necessary to improve the sensitivity of target degradation. Guided by our previous SAR investigations on evodiamine, EVO-PROTAC **13c** (IC_50_ = 0.019 μM) and **13e** (IC_50_ = 0.021 μM) showed excellent antiproliferative activity against HCT116 cells. Data analysis of quantitative proteomic assay is a bottleneck to identify a potential target. Herein negative PROTACs (**14a** and **14b**) were designed to avoid false positives. Furthermore, to assure the reliability of the target identification, two active PROTACs were subjected for quantitative proteomic analysis and the common proteins degraded by the two EVO-PROTACs but not by the negative controls were selected as potential targets.

Based on the integrated strategies, REXO4 was confirmed to be a direct target of evodiamine derivative **2**. Previous studies indicated that REXO4 was involved in neuropathic pain and familial isolated pituitary adenoma (FIPA) [45]. However, the relationship between REXO4 and tumor was poorly understood. The downregulation of REXO4 in breast cancer MDA-MB-231 cells resulted in improved sensitivity of temozolomide by increasing the number of apurinic/apyrimidinic sites in the DNA and the enhancement of DNA double strand breaks [55]. In addition, a clinical study indicated that the REXO4 expression level could act as a biomarker for targeted therapeutic regimens and predictor for the prognosis of liver cancer [46], [47]. The proliferation and progression of hepatocellular carcinoma (HCC) could be inhibited by the downregulation of REXO4 [46], [47]. Our studies confirmed that REXO4 is a direct target of 3-fluoro-10-hydroxylevodiamine. To our knowledge, it is the first small molecule binder of REXO4. Importantly, degradation of REXO4 resulted in potent *in vitro* and *in vivo* antitumor activity. Thus, REXO4 showed the potential to be an antitumor target. Also, the designed EVO-PROTACs provide valuable tools for better understanding the role of REXO4 in tumor.

Notably, evodiamine derivatives might act by multi-targeting mechanisms [56]. Our previous studies manifested that evodiamine and derivatives were weak binders of topoisomerase 1 and 2 and tubulin [37]. Further analysis of the proteomic data and subsequent validation are required to identify more potential targets (*e.g.* FAAH and CYBA). Also, further optimization of EVO-PROTACs is necessary to improve REXO4 degrading activity and antitumor potency.

## Conclusion

In summary, we developed an integrated strategy for the target identification and drug discovery of NPs, which was successfully applied to the PROTAC derivatization and target characterization of evodiamine. To improve the efficiency and reliability of the target identification, both highly potent PROTACs and negative controls were designed for quantitative proteomic analysis. REXO4 was confirmed as a direct target of evodiamine derivative **2**, which induced cell death through ROS. The EVO-PROTACs effectively degraded REXO4, leading to excellent *in vivo* antitumor activity and reduced toxicity. Thus, this study also prompted better understanding of the relationship between REXO4 and tumor. REXO4 may represent a potential target for the development of novel antitumor agents. Notably, evodiamine derivatives act by multi-targeting mechanisms and further analysis of the proteomic data is required to identify more potential targets. Taken together, this proof-of-concept study highlighted the superiority of PROTAC technology in target identification of NPs and accelerated the process of NPs-based drug discovery.

## Declaration of Competing Interest

The authors declare that they have no known competing financial interests or personal relationships that could have appeared to influence the work reported in this paper.

## References

[1] Cragg G.M., Newman D.J. (1830). Natural products: a continuing source of novel drug leads. BBA.

[2] Butler M.S., Robertson A.A., Cooper M.A. (2014). Natural product and natural product derived drugs in clinical trials. Nat Prod Rep.

[3] Newman D.J., Cragg G.M. (2020). Natural products as sources of new drugs over the nearly four decades from 01/1981 to 09/2019. J Nat Prod.

[4] Yao H., Liu J., Xu S., Zhu Z., Xu J. (2017). The structural modification of natural products for novel drug discovery. Exp Opin Drug Disc.

[5] Eder J., Sedrani R., Wiesmann C. (2014). The discovery of first-in-class drugs: origins and evolution. Nat Rev Drug Discov.

[6] Moffat J.G., Rudolph J., Bailey D., D. (2014). Phenotypic screening in cancer drug discovery-past, present and future. Nat Rev Drug Discov.

[7] Robles O., Romo D. (2014). Chemo- and site-selective derivatizations of natural products enabling biological studies. Nat Prod Rep.

[8] Baell J.B. (2016). Feeling Nature's PAINS: natural products, natural product drugs, and Pan Assay Interference Compounds (PAINS). J Nat Prod.

[9] Zhao L., Zhao J., Zhong K., Tong A., Jia D. (2022). Targeted protein degradation: mechanisms, strategies and application. Sig Transduct Target Ther 7.

[10] Lai A.C., Crews C.M. (2017). Induced protein degradation: an emerging drug discovery paradigm. Nat Rev Drug Discov.

[11] Zhang Y., Loh C., Chen J., Mainolfi N. (2019). Targeted protein degradation mechanisms. Drug Disc Today: Technol.

[12] Toure M., Crews C.M. (2016). Small-Molecule PROTACS: new approaches to protein degradation. Angew Chem Int Ed Engl.

[13] Deshaies R.J. (2015). Protein degradation: prime time for PROTACs. Nat Chem Biol.

[14] Zeng S., Huang W., Zheng X., Liyan C., Zhang Z., Wang J. (2021). Proteolysis targeting chimera (PROTAC) in drug discovery paradigm: recent progress and future challenges. Eur J Med Chem.

[15] Li J., Cai Z., Li X.W., Zhuang C. (2022). Natural product-inspired targeted protein degraders: advances and perspectives. J Med Chem.

[16] Wu Y., Yang Y., Wang W., Sun D., Liang J., Zhu M. (2022). PROTAC technology as a novel tool to identify the target of lathyrane diterpenoids. Acta Pharm Sin B.

[17] Yu H., Jin H., Gong W., Wang Z., Liang H. (2013). Pharmacological actions of multi-target-directed evodiamine. Molecules.

[18] Sun Q., Xie L., Song J., Li X. (2020). Evodiamine: a review of its pharmacology, toxicity, pharmacokinetics and preparation researches. ScienceDirect J. Ethnopharmacol..

[19] Dong G., Wang S., Miao Z., Yao J., Zhang Y., Guo Z. (2012). New tricks for an old natural product: discovery of highly potent evodiamine derivatives as novel antitumor agents by systemic structure-activity relationship analysis and biological evaluations. J Med Chem.

[20] Chen S., Bi K., Wu S., Li Y., Huang Y., Sheng C. (2021). Water-soluble derivatives of evodiamine: Discovery of evodiamine-10-phosphate as an orally active antitumor lead compound. Eur J Med Chem.

[21] Wang L., Fang K., Cheng J., Li Y., Huang Y., Chen S. (2020). Scaffold hopping of natural product evodiamine: discovery of a novel antitumor scaffold with excellent potency against colon cancer. J Med Chem.

[22] Xu S., Yao H., Qiu Y., Zhou M., Li D., Wu L. (2021). Discovery of novel polycyclic heterocyclic derivatives from evodiamine for the potential treatment of triple-negative breast cancer. J Med Chem.

[23] Fan X., Deng J., Shi T., Wen H., Li J., Liang Z. (2021). Design, synthesis and bioactivity study of evodiamine derivatives as multifunctional agents for the treatment of hepatocellular carcinoma. Bioorg Chem.

[24] Huang Y., Chen S., Wu S., Dong G., Sheng C. (2020). Evodiamine-inspired dual inhibitors of histone deacetylase 1 (HDAC1) and topoisomerase 2 (TOP2) with potent antitumor activity. Acta Pharm Sin B.

[25] Hu X., Jiao R., Li H., Wang X., Lyu H., Gao X. (2018). Antiproliferative hydrogen sulfide releasing evodiamine derivatives and their apoptosis inducing properties. Eur J Med Chem.

[26] Liu X.M., Li Z., Xie X.R., Wang J.Q., Qiao X., Qiao X. (2023). Combination of DNA damage, autophagy, and ERK inhibition: novel evodiamine-inspired multi-action Pt(IV) prodrugs with high-efficiency and low-toxicity antitumor activity. J Med Chem.

[27] Lei F., Xiong Y., Wang Y., Zhang H., Liang Z., Li J. (2022). Design, synthesis, and biological evaluation of novel evodiamine derivatives as potential antihepatocellular carcinoma agents. J Med Chem.

[28] Ma Z., Huang Y., Wan K., Zhu F., Dong G. (2021). Structural simplification of evodiamine: discovery of novel tetrahydro-β-carboline derivatives as potent antitumor agents. Bioorg Med Chem Lett.

[29] Liang H., Wang W., Zhu F., Chen S., Liu D., Sheng C. (2022). Discovery of novel bis-evodiamine derivatives with potent antitumor activity. Bioorg Med Chem.

[30] He S., Dong G., Wang Z., Chen W., Huang Y., Li Z. (2015). Discovery of novel multiacting topoisomerase I/II and histone deacetylase inhibitors. ACS Med Chem Lett.

[31] Puppala D., Lee H., Kim K., Swanson H. (2008). Development of an aryl hydrocarbon receptor antagonist using the proteolysis-targeting chimeric molecules approach: a potential tool for chemoprevention. Mol Pharmacol.

[32] Bian J., Ren J., Li Y., Wang J., Xu X., Feng Y. (2018). Discovery of Wogonin-based PROTACs against CDK9 and capable of achieving antitumor activity. Bioorg Chem.

[33] Kim S., Lim S., Choi J. (2022). Drug discovery inspired by bioactive small molecules from nature. Anim Cells Syst.

[34] Liu M., Martyn A., Quinn R. (2022). Natural product-based PROteolysis TArgeting Chimeras (PROTACs). Nat Prod Rep.

[35] Sakamoto K., Kim K., Kumagai A., Mercurio F., Crews C., Deshaies R. (2001). Protacs: chimeric molecules that target proteins to the Skp1-Cullin-F box complex for ubiquitination and degradation. PNAS.

[36] Dong G., Sheng C., Wang S., Miao Z., Yao J., Zhang W. (2010). Selection of evodiamine as a novel topoisomerase I inhibitor by structure-based virtual screening and hit optimization of evodiamine derivatives as antitumor agents. J Med Chem.

[37] Wang S., Fang K., Dong G., Chen S., Liu N., Miao Z. (2015). Scaffold diversity inspired by the natural product evodiamine: discovery of highly potent and multitargeting antitumor agents. J Med Chem.

[38] Cheng J., Li Y., Wang X., Dong G., Sheng C. (2020). Discovery of novel PDEdelta degraders for the treatment of KRAS mutant colorectal cancer. J Med Chem.

[39] He S., Ma J., Fang Y., Liu Y., Wu S., Dong G. (2021). Homo-PROTAC mediated suicide of MDM2 to treat non-small cell lung cancer. Acta Pharm Sin B.

[40] Cheng J., He S., Xu J., Huang M., Dong G., Sheng C. (2022). Making protein degradation visible: discovery of theranostic PROTACs for detecting and degrading NAMPT. J Med Chem.

[41] He S., Dong G., Cheng J., Wu Y., Sheng C. (2022). Strategies for designing proteolysis targeting chimaeras (PROTACs). Med Res Rev.

[42] He S., Gao F., Ma J., Ma H., Dong G., Sheng C. (2021). Aptamer-PROTAC Conjugates (APCs) for tumor-specific targeting in breast cancer. Angew Chem Int Ed.

[43] Wu Y., Pu C., Fu Y., Dong G., Huang M., Sheng C. (2022). NAMPT-targeting PROTAC promotes antitumor immunity via suppressing myeloid-derived suppressor cell expansion. Acta Pharm Sin B.

[44] Yao T., Xiao H., Wang H., Xu X. (2022). Recent advances in PROTACs for drug targeted protein research. Int J Mol Sci.

[45] Cai G., Zhu Y., Zhao Y., Chen J., Guo C., Wu F. (2020). Network analysis of miRNA and mRNA changes in the prelimbic cortex of rats with chronic neuropathic pain: pointing to inflammation. Front Genet.

[46] Ruan Y., Chen W., Gao C., Xu Y., Shi M., Zhou Z. (2021). REXO4 acts as a biomarker and promotes hepatocellular carcinoma progression. J Gastrointest Oncol.

[47] Chen W., Gao C., Shen J., Yao L., Liang X., Chen Z. (2021). The expression and prognostic value of REXO4 in hepatocellular carcinoma. J Gastrointest Oncol.

[48] Krishnamurthy N., Ngam C.R., Berdis A.J., Montano M.M. (2011). The exonuclease activity of hPMC2 is required for transcriptional regulation of the QR gene and repair of estrogen-induced abasic sites. Oncogene.

[49] Ray P.D., Huang B.W., Tsuji Y. (2012). Reactive oxygen species (ROS) homeostasis and redox regulation in cellular signaling. Cell. Signal..

[50] Filomeni G., De Zio D., Cecconi F. (2015). Oxidative stress and autophagy: the clash between damage and metabolic needs. Cell Death Differ.

[51] Mah L.J., El-Osta A., Karagiannis T.C. (2010). γH2AX: a sensitive molecular marker of DNA damage and repair. Leukemia.

[52] Cui Z., Li C., Chen P., Yang H. (2022). An update of label-free protein target identification methods for natural active products. Theranostics.

[53] Pan S., Zhang H., Wang C., Yao S.C., Yao S.Q. (2016). Target identification of natural products and bioactive compounds using affinity-based probes. Nat Prod Rep.

[54] Yoshida M. (2019). Recent advances in target identification of bioactive natural products. Biosci Biotech Bioch.

[55] Krishnamurthy N., Liu L., Xiong X., Zhang J., Montano M.M. (2015). Downregulation of hPMC2 imparts chemotherapeutic sensitivity to alkylating agents in breast cancer cells. Cancer Biol. Ther..

[56] Fan M., Yao L. (2022). The synthesis, structural modification and mode of anticancer action of evodiamine: a review. Recent Pat Anticancer Drug Discov.

