# Supplementary material for: PROTAC derivatization of natural products for target identification and drug discovery: Design of evodiamine-based PROTACs as novel REXO4 degraders
Source: J Adv Res. 2023 Oct 31;63:219–30. doi: 10.1016/j.jare.2023.10.014 (PMC11380026; doi:10.1016/j.jare.2023.10.014)
Supplement: Supplementary data 1 [file mmc1.docx]

Supplementary Information

**PROTAC Derivatization of Natural Products for Target Identification and Drug Discovery: Design of Evodiamine-based PROTACs as Novel REXO4 Degraders**

**Table of Contents**

**Experiment methods** S3

[**Figure S1.** The stability test of compounds **6a** and **13c** S16](#_Toc117414919)

[**Figure S2.** EVO-PROTAC **13e** induce the REXO4 protein degradation through the UPS. S16](#_Toc117414920)

[**Representative ^1^H-NMR, ^13^C-NMR and ESI-MS of EVO-PROTACs** S17](#_Toc117414937)

[**Representative HPLC Purity of PROTACs** S44](#_Toc117414937)

**The unprocessed images of western blot**................................................................S48

**References**.................................................................................................................S49

**Experiment methods**

*General Chemistry Methods*

^1^H NMR and ^13^C NMR spectra were performed on AVANCE300 or AVANCE600 spectrometers (Bruker Company, Germany), DMSO-*d*_6_ or CDCl_3_ were served as the solvent and TMS as the internal standard. Coupling constants (*J*) and chemical shifts (*δ*) are reported in Hz and ppm, respectively. The mass spectra were conducted with an Esquire 3000 LC-MS mass spectrometer. Precoated plates GF254 (Qingdao Haiyang Chemical, China) were used for the analytical TLC, and silica gel 60 G (Qingdao Haiyang Chemical, China) were adopted for silica gel column chromatography separations. The purities of the target compounds were determined by HPLC (Agilent Technologies 1260 Inﬁnity) using MeOH/H_2_O as the mobile phase at a ﬂow rate of 0.4 mL/min on a C18 column (Agilent 20RBA × SB-C18, 5 μm, 4.6 mm × 150 mm). All final compounds exhibited purities greater than 95%.

**3-Fluoro-14-methyl-5-oxo-5,7,8,13,13b,14-hexahydroindolo[2',3':3,4]pyrido[2,1-b]quinazolin-10-yl 7-(2-(2,6-dioxopiperidin-3-yl)-1-oxoisoindolin-4-yl)hept-6-ynoate (6b)**. Pale yellow solid, 73 mg, yield 66%. ^1^H NMR (600 MHz, DMSO-*d*_6_): *δ* 11.30 (s, 1H), 10.97 (s, 1H), 7.71 (d, *J* = 7.6 Hz, 1H), 7.64 (d, *J* = 7.6 Hz, 1H), 7.54 (dd, *J* = 8.9, 3.1 Hz, 1H), 7.51 (t, *J* = 7.6 Hz, 1H), 7.41-7.37 (m, 1H), 7.35 (d, *J* = 8.7 Hz, 1H), 7.23-7.17 (m, 2H), 6.86 (dd, *J* = 8.7, 2.2 Hz, 1H), 6.10 (s, 1H), 5.16-5.04 (m, 1H), 4.66-4.57 (m, 1H), 4.46 (d, *J* = 17.5 Hz, 1H), 4.33 (dd, *J* = 17.7, 3.3 Hz, 1H), 3.24-3.15 (m, 1H), 2.89-2.77 (m, 3H), 2.68 (s, 3H), 2.66 (t, *J* = 7.3 Hz, 2H), 2.57 (t, *J* = 6.9 Hz, 2H), 2.49-2.34 (m, 2H), 1.98-1.91 (m, 1H), 1.88-1.80 (m, 2H), 1.74-1.66 (m, 2H). ^13^C NMR (150 MHz, DMSO-*d*_6_): *δ* 172.73, 172.36, 170.89, 167.62, 162.99, 157.16 (d, *J* = 239.1 Hz), 145.98, 143.75, 143.65, 134.35, 134.04, 131.97, 131.10, 128.57, 125.84, 122.61, 122.07 (d, *J* = 6.8 Hz), 121.77 (d, *J* = 7.3 Hz), 120.56 (d, *J* = 23.5 Hz), 118.77, 116.31, 113.37 (d, *J* = 23.6 Hz), 111.98, 111.83, 110.56, 96.01, 76.63, 69.15, 51.61, 47.03, 40.26, 36.68, 33.00, 31.10, 27.38, 23.74, 22.25, 19.46, 18.50. HR-ESI-MS calcd for C_39_H_34_FN_5_O_6_ [M+H]^+^ 688.2566, found 688.2567.

**3-Fluoro-14-methyl-5-oxo-5,7,8,13,13b,14-hexahydroindolo[2',3':3,4]pyrido[2,1-b]quinazolin-10-yl 8-(2-(2,6-dioxopiperidin-3-yl)-1-oxoisoindolin-4-yl)oct-7-ynoate (6c)**. Pale yellow solid, 61 mg, yield 69%. ^1^H NMR (600 MHz, DMSO-*d*_6_): *δ* 11.30 (s, 1H), 10.98 (s, 1H), 7.70 (d, *J* = 7.8 Hz, 1H), 7.63 (d, *J* = 7.6 Hz, 1H), 7.54 (dd, *J* = 8.8, 3.1 Hz, 1H), 7.50 (t, *J* = 7.6 Hz, 1H), 7.42-7.36 (m, 1H), 7.31 (d, *J* = 8.7 Hz, 1H), 7.23-7.16 (m, 2H), 6.83 (dd, *J* = 8.7, 2.2 Hz, 1H), 6.10 (s, 1H), 5.10 (dd, *J* = 12.7, 4.3 Hz, 1H), 4.60 (dd, *J* = 12.8, 3.4 Hz, 1H), 4.44 (d, *J* = 17.4 Hz, 1H), 4.31 (dd, *J* = 17.6, 2.2 Hz, 1H), 3.23-3.15 (m, 1H), 2.91-2.83 (m, 1H), 2.82-2.74 (m, 2H), 2.67 (d, *J* = 2.6 Hz, 3H), 2.60 (t, *J* = 7.3 Hz, 2H), 2.56-2.51 (m, 3H), 2.45-2.35 (m, 1H), 1.97-1.89 (m, 1H), 1.76-1.69 (m, 2H), 1.68-1.61 (m, 2H), 1.60-1.52 (m, 2H). ^13^C NMR (150 MHz, DMSO-*d*_6_): *δ* 172.78, 172.45, 170.93, 167.62, 163.00, 157.17 (d, *J* = 238.7 Hz), 145.98, 143.72, 143.65, 134.34, 134.05, 131.95, 131.10, 128.54, 125.84, 122.57, 122.08 (d, *J* = 5.9 Hz), 121.80 (d, *J* = 7.3 Hz), 120.57 (d, *J* = 23.3 Hz), 118.82, 116.30, 113.38 (d, *J* = 23.1 Hz), 111.96, 111.80, 110.55, 96.22, 76.50, 69.15, 51.62, 46.96, 40.26, 36.69, 33.48, 31.14, 27.69, 24.02, 22.28, 19.45, 18.65. HR-ESI-MS calcd for C_40_H_36_FN_5_O_6_ [M-H]^-^ 700.2577, found 700.2569.

**3-Fluoro-14-methyl-5-oxo-5,7,8,13,13b,14-hexahydroindolo[2',3':3,4]pyrido[2,1-b]quinazolin-10-yl 9-(2-(2,6-dioxopiperidin-3-yl)-1-oxoisoindolin-4-yl)non-8-ynoate (6d)**. Pale yellow solid, 46 mg, yield 61%. ^1^H NMR (600 MHz, DMSO-*d*_6_): *δ* 11.29 (s, 1H), 10.99 (s, 1H), 7.70 (d, *J* = 7.5 Hz, 1H), 7.63 (d, *J* = 7.6 Hz, 1H), 7.54 (dd, *J* = 8.8, 3.1 Hz, 1H), 7.50 (t, *J* = 7.6 Hz, 1H), 7.42-7.35 (m, 1H), 7.35 (d, *J* = 8.7 Hz, 1H), 7.24-7.17 (m, 2H), 6.84 (dd, *J* = 8.7, 2.2 Hz, 1H), 6.10 (s, 1H), 5.12 (dd, *J* = 13.3, 5.1 Hz, 1H), 4.65-4.58 (m, 1H), 4.45 (d, *J* = 17.5 Hz, 1H), 4.32 (d, *J* = 17.5 Hz, 1H), 3.24-3.16 (m, 1H), 2.93-2.85 (m, 1H), 2.85-2.78 (m, 2H), 2.68 (s, 3H), 2.61-2.51 (m, 5H), 2.48-2.38 (m, 1H), 2.03-1.95 (m, 1H), 1.73-1.65 (m, 2H), 1.65-1.58 (m, 2H), 1.54-1.47 (m, 2H), 1.46-1.40 (m, 2H). ^13^C NMR (150 MHz, DMSO-*d*_6_): *δ* 172.81, 172.48, 170.95, 167.65, 163.01, 157.17 (d, *J* = 239.1 Hz), 145.97, 143.72, 143.67, 134.35, 134.04, 131.95, 131.11, 128.55, 125.86, 122.56, 122.07 (d, *J* = 7.2 Hz), 121.77 (d, *J* = 7.4 Hz), 120.57 (d, *J* = 23.5 Hz), 118.84, 116.34, 113.39 (d, *J* = 23.4 Hz), 111.98, 111.83, 110.57, 96.28, 76.45, 69.17, 51.63, 46.96, 40.28, 36.69, 33.47, 31.17, 27.92, 27.87, 24.38, 22.34, 19.48, 18.69. HR-ESI-MS calcd for C_41_H_38_FN_5_O_6_ [M+H]^+^ 716.2879, found 716.2873.

**3-Fluoro-14-methyl-5-oxo-5,7,8,13,13b,14-hexahydroindolo[2',3':3,4]pyrido[2,1-b]quinazolin-10-yl 10-(2-(2,6-dioxopiperidin-3-yl)-1-oxoisoindolin-4-yl)dec-9-ynoate (6e)**. Pale yellow solid, 55 mg, yield 63%. ^1^H NMR (600 MHz, DMSO-*d*_6_): *δ* 11.30 (s, 1H), 11.00 (s, 1H), 7.70 (d, *J* = 7.6 Hz, 1H), 7.63 (d, *J* = 7.7 Hz, 1H), 7.54 (dd, *J* = 8.9, 3.1 Hz, 1H), 7.50 (t, *J* = 7.6 Hz, 1H), 7.42-7.36 (m, 1H), 7.35 (d, *J* = 8.7 Hz, 1H), 7.23-7.16 (m, 2H), 6.84 (dd, *J* = 8.7, 2.2 Hz, 1H), 6.10 (s, 1H), 5.14 (dd, *J* = 13.3, 5.1 Hz, 1H), 4.64-4.57 (m, 1H), 4.44 (d, *J* = 17.6 Hz, 1H), 4.31 (d, *J* = 17.6 Hz, 1H), 3.24-3.15 (m, 1H), 2.95-2.85 (m, 1H), 2.85-2.78 (m, 2H), 2.68 (s, 3H), 2.63-2.54 (m, 3H), 2.49-2.38 (m, 3H), 2.05-1.96 (m, 1H), 1.70-1.63 (m, 2H), 1.63-1.56 (m, 2H), 1.51-1.43 (m, 2H), 1.43-1.34 (m, 4H). ^13^C NMR (150 MHz, DMSO-*d*_6_): *δ* 172.81, 172.47, 170.95, 167.63, 162.99, 157.15 (d, *J* = 239.0 Hz), 145.96, 143.70, 143.66, 134.33, 134.02, 131.95, 131.11, 128.56, 125.85, 122.55, 122.06 (d, *J* = 7.2 Hz), 121.76 (d, *J* = 7.3 Hz), 120.57 (d, *J* = 23.0 Hz), 118.83, 116.33, 113.37 (d, *J* = 23.1 Hz), 111.97, 111.81, 110.55, 96.34, 76.39, 69.16, 51.61, 46.93, 40.27, 36.68, 33.50, 31.16, 28.33, 28.10, 27.98, 24.39, 22.35, 19.47, 18.71. HR-ESI-MS calcd for C_42_H_40_FN_5_O_6_ [M+H]^+^ 730.3035, found 730.3022.

**3-Fluoro-14-methyl-5-oxo-5,7,8,13,13b,14-hexahydroindolo[2',3':3,4]pyrido[2,1-b]quinazolin-10-yl 8-(2-(1-methyl-2,6-dioxopiperidin-3-yl)-1-oxoisoindolin-4-yl)oct-7-ynoate (9a).** The synthetic route was similar to compound **7a**. Yellow solid, 40 mg, yield 67%. ^1^H NMR (600 MHz, DMSO-*d*_6_): *δ* 11.29 (s, 1H), 7.71 (d, *J* = 7.5 Hz, 1H), 7.63 (d, *J* = 7.5 Hz, 1H), 7.54 (dd, *J* = 8.8, 3.0 Hz, 1H), 7.50 (t, *J* = 7.6 Hz, 1H), 7.42-7.36 (m, 1H), 7.32 (d, *J* = 8.6 Hz, 1H), 7.22-7.17 (m, 2H), 6.83 (dd, *J* = 8.7, 2.2 Hz, 1H), 6.09 (s, 1H), 5.21-5.15 (m, 1H), 4.60 (dd, *J* = 13.0, 3.4 Hz, 1H), 4.44 (d, *J* = 17.6 Hz, 1H), 4.31 (d, *J* = 17.6 Hz, 1H), 3.23-3.14 (m, 1H), 2.99 (s, 3H), 2.98-2.90 (m, 1H), 2.85-2.74 (m, 2H), 2.73-2.65 (m, 4H), 2.60 (t, *J* = 7.3 Hz, 2H), 2.53 (t, *J* = 6.6 Hz,, 2H), 2.45-2.34 (m, 1H), 1.99-1.91 (m, 1H), 1.75-1.69 (m, 2H), 1.67-1.61 (m, 2H), 1.60-1.52 (m, 2H). ^13^C NMR (150 MHz, DMSO-*d*_6_): *δ* 172.41, 171.79, 170.52, 167.63, 162.98, 157.15 (d, *J* = 238.7 Hz), 145.97, 143.73, 143.63, 134.33, 134.07, 131.92, 131.09, 128.53, 125.83, 122.59, 122.07 (d, *J* = 6.9 Hz), 121.77 (d, *J* = 7.4 Hz), 120.55 (d, *J* = 23.3 Hz), 118.81, 116.27, 113.36 (d, *J* = 23.4 Hz), 111.94, 111.79, 110.51, 96.17, 76.53, 69.14, 52.08, 46.91, 40.24, 36.67, 33.47, 31.28, 27.67, 26.52, 24.01, 21.55, 19.43, 18.63. HR-ESI-MS calcd for C_41_H_38_FN_5_O_6_ [M-H]^-^ 714.2733, found 714.2739.

**3-Fluoro-14-methyl-5-oxo-5,7,8,13,13b,14-hexahydroindolo[2',3':3,4]pyrido[2,1-b]quinazolin-10-yl 10-(2-(1-methyl-2,6-dioxopiperidin-3-yl)-1-oxoisoindolin-4-yl)dec-9-ynoate (9b).** Yellow solid, 27 mg, yield 56%. ^1^H NMR (600 MHz, DMSO-*d*_6_): *δ* 11.29 (s, 1H), 7.71 (dd, *J* = 7.6, 0.8 Hz, 1H), 7.63 (dd, *J* = 7.6, 0.8 Hz, 1H), 7.54 (dd, *J* = 8.9, 3.1 Hz, 1H), 7.51 (t, *J* = 7.6 Hz, 1H), 7.41-7.36 (m, 1H), 7.35 (d, *J* = 8.7 Hz, 1H), 7.22-7.18 (m, 2H), 6.84 (dd, *J* = 8.7, 2.3 Hz, 1H), 6.10 (s, 1H), 5.20 (dd, *J* = 13.5, 5.1 Hz, 1H), 4.64-4.58 (m, 1H), 4.44 (d, *J* = 17.6 Hz, 1H), 4.31 (d, *J* = 17.6 Hz, 1H), 3.24-3.16 (m, 1H), 3.00 (s, 3H), 3.02-2.94 (m, 1H), 2.87-2.78 (m, 2H), 2.76-2.71 (m, 1H), 2.68 (s, 3H), 2.56 (t, *J* = 7.4 Hz, 2H), 2.49 (t, *J* = 7.8 Hz, 2H), 2.47-2.38 (m, 1H), 2.05-1.98 (m, 1H), 1.70-1.63 (m, 2H), 1.63-1.56 (m, 2H), 1.50-1.43 (m, 2H), 1.43-1.33 (m, 4H). ^13^C NMR (150 MHz, DMSO-*d*_6_): *δ* 172.41 , 171.80, 170.53, 167.64, 162.96, 157.13 (d, *J* = 238.8 Hz), 145.94, 143.67, 143.64, 134.32, 134.05, 131.92, 131.09, 128.53, 125.83, 122.56, 122.04 (d, *J* = 7.1 Hz), 121.72 (d, *J* = 7.4 Hz), 120.53 (d, *J* = 23.3 Hz), 118.82, 116.28, 113.35 (d, *J* = 23.4 Hz), 111.94, 111.80, 110.51, 96.28, 76.39, 69.15, 52.09, 46.89, 40.25, 36.66, 33.47, 31.29, 28.30, 28.07, 27.95, 26.51, 24.36, 21.60, 19.45, 18.69. HR-ESI-MS calcd for C_43_H_42_FN_5_O_6_ [M+H]^+^ 744.3192, found 744.3189.

**7-(2-(2,6-Dioxopiperidin-3-yl)-1-oxoisoindolin-4-yl)-*N*-(3-((3-fluoro-14-methyl-5-oxo-5,7,8,13,13b,14-hexahydroindolo[2',3':3,4]pyrido[2,1-b]quinazolin-10-yl)oxy)propyl)hept-6-ynamide (13b).** Yellow solid, 27 mg, yield 53%. ^1^H NMR (600 MHz, DMSO-*d*_6_): *δ* 10.98 (s, 2H), 7.90 (t, *J* = 5.5 Hz, 1H), 7.70 (d, *J* = 7.5 Hz, 1H), 7.60 (d, *J* = 7.0 Hz, 1H), 7.53 (dd, *J* = 8.9, 3.1 Hz, 1H), 7.49 (t, *J* = 7.6 Hz, 1H), 7.41-7.35 (m, 1H), 7.24 (d, *J* = 8.8 Hz, 1H), 7.17 (dd, *J* = 8.9, 4.5 Hz, 1H), 6.98 (d, *J* = 2.1 Hz, 1H), 6.76 (dd, *J* = 8.8, 2.3 Hz, 1H), 6.05 (s, 1H), 5.14 (dd, *J* = 13.2, 5.1 Hz, 1H), 4.65-4.55 (m, 1H), 4.45 (d, *J* = 17.7 Hz, 1H), 4.32 (d, *J* = 17.8 Hz, 1H), 3.97 (t, *J* = 6.3 Hz, 2H), 3.25-3.14 (m, 3H), 2.96-2.87 (m, 1H), 2.86-2.76 (m, 2H), 2.67 (d, *J* = 0.8 Hz, 3H), 2.62-2.56 (m, 1H), 2.56-2.51 (m, 1H), 2.48 (t, *J* = 6.9 Hz, 2H), 2.13 (t, *J* = 7.3 Hz, 2H), 2.03-1.96 (m, 1H), 1.89-1.81 (m, 2H), 1.72-1.64 (m, 2H), 1.59-1.50 (m, 2H). ^13^C NMR (150 MHz, DMSO-*d*_6_): *δ* 172.82, 171.85, 170.94, 167.66, 163.01, 157.06 (d, *J* = 238.5 Hz), 152.56, 145.96, 143.82, 133.91, 131.94, 131.75, 130.22, 128.53, 126.10, 122.56, 121.93 (d, *J* = 6.7 Hz), 121.53 (d, *J* = 7.34 Hz), 120.53 (d, *J* = 23.2 Hz), 118.80, 113.36 (d, *J* = 22.9 Hz), 112.56, 112.27, 111.34, 101.32, 96.11, 76.56, 69.29, 65.82, 51.60, 46.98, 40.37, 36.56, 35.57, 34.76, 31.20, 29.16, 27.57, 24.46, 22.27, 19.59, 18.50. HR-ESI-MS calcd for C_42_H_41_FN_6_O_6_ [M+H]^+^ 745.3144, found 745.3137.

**8-(2-(2,6-Dioxopiperidin-3-yl)-1-oxoisoindolin-4-yl)-*N*-(3-((3-fluoro-14-methyl-5-oxo-5,7,8,13,13b,14-hexahydroindolo[2',3':3,4]pyrido[2,1-b]quinazolin-10-yl)oxy)propyl)oct-7-ynamide (13c).** Yellow solid, 52 mg, yield 56%. ^1^H NMR (600 MHz, DMSO-*d*_6_): *δ* 11.00 (s, 2H), 7.86 (t, *J* = 5.5 Hz, 1H), 7.69 (d, *J* = 7.6 Hz, 1H), 7.61 (d, *J* = 7.6 Hz, 1H), 7.53 (dd, *J* = 8.9, 3.1 Hz, 1H), 7.49 (t, *J* = 7.6 Hz, 1H), 7.40-7.35 (m, 1H), 7.24 (d, *J* = 8.7 Hz, 1H), 7.17 (dd, *J* = 8.9, 4.5 Hz, 1H), 6.97 (d, *J* = 2.1 Hz, 1H), 6.75 (dd, *J* = 8.8, 2.3 Hz, 1H), 6.06 (s, 1H), 5.13 (dd, *J* = 13.3, 5.1 Hz, 1H), 4.61 (dd, *J* = 11.5, 3.7 Hz, 1H), 4.44 (d, *J* = 17.7 Hz, 1H), 4.31 (d, *J* = 17.7 Hz, 1H), 3.95 (t, *J* = 6.3 Hz, 2H), 3.24-3.14 (m, 3H), 2.94-2.87 (m, 1H), 2.86-2.77 (m, 2H), 2.67 (s, 3H), 2.58 (d, *J* = 17.3 Hz, 1H), 2.49-2.40 (m, 3H), 2.09 (t, *J* = 7.3 Hz, 2H), 2.04-1.99 (m, 1H), 1.86-1.79 (m, 2H), 1.60-1.51 (m, 4H), 1.44-1.36 (m, 2H). ^13^C NMR (150 MHz, DMSO-*d*_6_): *δ* 172.82, 172.01, 170.96, 167.65, 163.02, 157.08 (d, *J* = 238.6 Hz), 152.56, 145.97, 143.71, 134.04, 131.96, 131.75, 130.21, 128.54, 126.10, 122.55, 121.96 (d, *J* = 7.0 Hz), 121.57 (d, *J* = 7.2 Hz), 120.54 (d, *J* = 23.0 Hz), 118.83, 113.37 (d, *J* = 23.5 Hz), 112.58, 112.28, 111.36, 101.28, 96.28, 76.40, 69.28, 65.78, 51.65, 46.98, 40.37, 36.56, 35.56, 35.30, 31.19, 29.14, 27.93, 27.81, 24.80, 22.32, 19.62, 18.65. HR-ESI-MS calcd for C_43_H_43_FN_6_O_6_ [M+H]^+^ 759.3301, found 759.3292.

**9-(2-(2,6-Dioxopiperidin-3-yl)-1-oxoisoindolin-4-yl)-*N*-(3-((3-fluoro-14-methyl-5-oxo-5,7,8,13,13b,14-hexahydroindolo[2',3':3,4]pyrido[2,1-b]quinazolin-10-yl)oxy)propyl)non-8-ynamide (13d).** Yellow solid, 49 mg, yield 50%. ^1^H NMR (600 MHz, DMSO-*d*_6_): *δ* 10.99 (s, 2H), 7.84 (t, *J* = 5.3 Hz, 1H), 7.69 (d, *J* = 7.4 Hz, 1H), 7.62 (d, *J* = 7.7 Hz, 1H), 7.53 (dd, *J* = 8.8, 3.1 Hz, 1H), 7.50 (t, *J* = 7.6 Hz, 1H), 7.40-7.34 (m, 1H), 7.24 (d, *J* = 8.7 Hz, 1H), 7.17 (dd, *J* = 8.9, 4.5 Hz, 1H), 6.98 (d, *J* = 2.2 Hz, 1H), 6.76 (dd, *J* = 8.8, 2.3 Hz, 1H), 6.05 (s, 1H), 5.13 (dd, *J* = 13.2, 5.3 Hz, 1H), 4.65-4.57 (m, 1H), 4.44 (d, *J* = 17.6 Hz, 1H), 4.31 (d, *J* = 17.7 Hz, 1H), 3.97 (t, *J* = 6.3 Hz, 2H), 3.26-3.14 (m, 3H), 2.95-2.87 (m, 1H), 2.86-2.76 (m, 2H), 2.67 (s, 3H), 2.62-2.56 (m, 1H), 2.45 (t, *J* = 6.9 Hz, 3H), 2.07 (t, *J* = 7.3 Hz, 2H), 2.03-1.98 (m, 1H), 1.87-1.81 (m, 2H), 1.58-1.48 (m, 4H), 1.45-1.37 (m, 2H), 1.31-1.26 (m, 2H). ^13^C NMR (150 MHz, DMSO-*d*_6_): *δ* 172.79, 172.07, 170.93, 167.64, 163.02, 157.06 (d, *J* = 239.0 Hz), 152.57, 145.95, 143.70, 134.00, 131.94, 131.75, 130.22, 128.54, 126.10, 122.53, 121.92 (d, *J* = 7.4 Hz), 121.51 (d, *J* = 8.1 Hz), 120.52 (d, *J* = 23.0 Hz), 118.83, 113.35 (d, *J* = 23.1 Hz), 112.57, 112.26, 111.34, 101.32, 96.29, 76.38, 69.29, 65.84, 51.65, 46.97, 40.37, 36.55, 35.55, 35.35, 31.17, 29.15, 28.10, 28.02, 27.90, 25.20, 22.34, 19.60, 18.67.HR-ESI-MS calcd for C_44_H_45_FN_6_O_6_ [M-H]^-^ 771.3312, found 771.3330.

**10-(2-(2,6-Dioxopiperidin-3-yl)-1-oxoisoindolin-4-yl)-*N*-(3-((3-fluoro-14-methyl-5-oxo-5,7,8,13,13b,14-hexahydroindolo[2',3':3,4]pyrido[2,1-b]quinazolin-10-yl)oxy)propyl)dec-9-ynamide (13e).** Yellow solid, yield 48%. ^1^H NMR (600 MHz, DMSO-*d*_6_): *δ* 10.99 (d, *J* = 2.7 Hz, 2H), 7.84 (t, *J* = 5.6 Hz, 1H), 7.69 (d, *J* = 7.6 Hz, 1H), 7.61 (d, *J* = 7.1 Hz, 1H), 7.53 (dd, *J* = 8.9, 3.1 Hz, 1H), 7.49 (t, *J* = 7.6 Hz, 1H), 7.40-7.34 (m, 1H), 7.24 (d, *J* = 8.7 Hz, 1H), 7.17 (dd, *J* = 8.9, 4.5 Hz, 1H), 6.97 (d, *J* = 2.2 Hz, 1H), 6.76 (dd, *J* = 8.8, 2.4 Hz, 1H), 6.05 (s, 1H), 5.13 (dd, *J* = 13.3, 5.1 Hz, 1H), 4.64-4.58 (m, 1H), 4.43 (d, *J* = 17.7 Hz, 1H), 4.30 (d, *J* = 17.6 Hz, 1H), 3.96 (t, *J* = 6.4 Hz, 2H), 3.24-3.15 (m, 3H), 2.93-2.87 (m, 1H), 2.85-2.77 (m, 2H), 2.67 (s, 3H), 2.59 (d, *J* = 15.3 Hz, 1H), 2.47-2.39 (m, 3H), 2.06 (t, *J* = 7.4 Hz, 2H), 2.03-1.99 (m, 1H), 1.87-1.81 (m, 2H), 1.56-1.47 (m, 4H), 1.43-1.35 (m, 2H), 1.30-1.23 (m, 4H). ^13^C NMR (150 MHz, DMSO-*d*_6_): *δ* 172.80, 172.12, 170.94, 167.65, 163.02, 157.06 (d, *J* = 238.5 Hz), 152.58, 145.96, 143.68, 134.02, 131.94, 131.76, 130.22, 128.55, 126.10, 122.53, 121.92 (d, *J* = 7.1 Hz), 121.52 (d, *J* = 7.4 Hz), 120.53 (d, *J* = 23.1 Hz), 118.84, 113.35 (d, *J* = 23.5 Hz), 112.58, 112.27, 111.35, 101.31, 96.35, 76.34, 69.30, 65.83, 51.65, 46.96, 40.37, 36.56, 35.54, 35.38, 31.17, 29.15, 28.54, 28.19, 28.15, 28.00, 25.21, 22.35, 19.61, 18.69. HR-ESI-MS calcd for C_45_H_47_FN_6_O_6_ [M+H]^+^ 787.3614, found 787.3622.

***N*-(3-((3-fluoro-14-methyl-5-oxo-5,7,8,13,13b,14-hexahydroindolo[2',3':3,4] pyrido[2,1-b]quinazolin-10-yl)oxy)propyl)-8-(2-(1-methyl-2,6-dioxopiperidin-3-yl)-1-oxoisoindolin-4-yl)oct-7-ynamide (14a).** The synthetic route was similar to compound **13a**. Yellow solid, 48 mg, yield 44%. ^1^H NMR (600 MHz, DMSO-*d*_6_): *δ* 10.99 (s, 1H), 7.85 (t, *J* = 5.6 Hz, 1H), 7.70 (d, *J* = 7.6 Hz, 1H), 7.62 (d, *J* = 7.6 Hz, 1H), 7.53 (dd, *J* = 8.9, 3.1 Hz, 1H), 7.50 (t, *J* = 7.6 Hz, 1H), 7.41-7.35 (m, 1H), 7.24 (d, *J* = 8.7 Hz, 1H), 7.17 (dd, *J* = 8.9, 4.5 Hz, 1H), 6.97 (d, *J* = 2.1 Hz, 1H), 6.75 (dd, *J* = 8.8, 2.3 Hz, 1H), 6.05 (s, 1H), 5.21 (dd, *J* = 13.4, 5.1 Hz, 1H), 4.61 (dd, *J* = 11.7, 3.9 Hz, 1H), 4.44 (d, *J* = 17.6 Hz, 1H), 4.31 (d, *J* = 17.6 Hz, 1H), 3.95 (t, *J* = 6.3 Hz, 2H), 3.24-3.14 (m, 3H), 3.00 (s, 3H), 3.03-2.93 (m, 1H), 2.87-2.77 (m, 2H), 2.74 (d, *J* = 14.1 Hz, 1H), 2.67 (s, 3H), 2.48-2.41 (m, 3H), 2.08 (t, *J* = 7.3 Hz, 2H), 2.05-1.98 (m, 1H), 1.87-1.79 (m, 2H), 1.60-1.50 (m, 4H), 1.45-1.35 (m, 2H). ^13^C NMR (150 MHz, DMSO-*d*_6_): *δ* 171.97, 171.82, 170.55, 167.65, 162.99, 157.06 (d, *J* = 238.4 Hz), 152.55 , 145.95 , 143.70 , 134.05 , 131.93 , 131.74 , 130.20 , 128.52 , 126.08 , 122.57 , 121.94 (d, *J* = 7.2 Hz), 121.54 (d, *J* = 7.4 Hz), 120.50 (d, *J* = 23.3 Hz), 118.82, 113.34 (d, *J* = 23.3 Hz), 112.55, 112.25, 111.34, 101.28, 96.21, 76.42, 69.27, 65.78, 52.11, 46.92, 40.35, 36.54, 35.53, 35.30, 31.32, 29.13, 27.90, 27.77, 26.52, 24.79, 21.59, 19.60, 18.63. HR-ESI-MS calcd for C_44_H_45_FN_6_O_6_ [M-H]^-^ 771.3312, found 771.3319.

***N*-(3-((3-fluoro-14-methyl-5-oxo-5,7,8,13,13b,14-hexahydroindolo[2',3':3,4] pyrido[2,1-b]quinazolin-10-yl)oxy)propyl)-10-(2-(1-methyl-2,6-dioxopiperidin-3-yl)-1-oxoisoindolin-4-yl)dec-9-ynamide (14b).** Yellow solid, 27 mg, yield 53%. ^1^H NMR (600 MHz, DMSO-*d*_6_): *δ* 10.98 (s, 1H), 7.82 (t, *J* = 5.6 Hz, 1H), 7.70 (d, *J* = 7.6 Hz, 1H), 7.61 (d, *J* = 7.6 Hz, 1H), 7.53 (dd, *J* = 8.9, 3.1 Hz, 1H), 7.50 (t, *J* = 7.6 Hz, 1H), 7.40-7.34 (m, 1H), 7.24 (d, *J* = 8.8 Hz, 1H), 7.17 (dd, *J* = 8.9, 4.5 Hz, 1H), 6.97 (d, *J* = 2.1 Hz, 1H), 6.76 (dd, *J* = 8.8, 2.2 Hz, 1H), 6.05 (s, 1H), 5.20 (dd, *J* = 13.5, 5.1 Hz, 1H), 4.61 (dd, *J* = 11.8, 4.1 Hz, 1H), 4.42 (d, *J* = 17.5 Hz, 1H), 4.30 (d, *J* = 17.6 Hz, 1H), 3.96 (t, *J* = 6.3 Hz, 2H), 3.24-3.15 (m, 3H), 3.00 (s, 3H), 3.04-2.94 (m, 1H), 2.87-2.78 (m, 2H), 2.77-2.72 (m, 1H), 2.67 (s, 3H), 2.48-2.37 (m, 3H), 2.09-1.99 (m, 3H), 1.88-1.80 (m, 2H), 1.58-1.46 (m, 4H), 1.42-1.35 (m, 2H), 1.30-1.23 (m, 4H). ^13^C NMR (150 MHz, DMSO-*d*_6_): *δ* 172.01, 171.80, 170.53, 167.64, 162.98, 157.04 (d, *J* = 238.4 Hz), 152.55, 145.93, 143.65, 134.06, 131.91, 131.74, 130.19, 128.52, 126.08, 122.54, 121.92 (d, *J* = 7.2 Hz), 121.50 (d, *J* = 7.4 Hz), 120.48 (d, *J* = 23.1 Hz), 118.81, 113.33 (d, *J* = 23.4 Hz), 112.55, 112.23, 111.31, 101.28, 96.26, 76.34, 69.26, 65.80, 52.10, 46.90, 40.34, 36.53, 35.50, 35.33, 31.30, 29.14, 28.49, 28.16, 28.12, 27.98, 26.51, 25.15, 21.60, 19.58, 18.67. HR-ESI-MS calcd for C_46_H_49_FN_6_O_6_ [M-H]^-^ 799.3625, found 799.3632.

*Cell Culture*

The HCT116 (human colon cancer), MCF-7 (human breast cancer), A549 (human lung cancer) cells were cultured in Dulbecco's Modified Eagle Medium (DMEM, Cytiva) medium supplemented with 10% (V/V) fetal bovine serum (FBS) (Biowest) and 1% (V/V) penicillin/streptomycin (Cytiva) at 37 °C, 5% CO_2_ and 95% humidity.

*In vitro stability assay*

2 × 10^6^ HCT116 cells were planted in a T25 culture dish with 5 mL of DMEM medium and cultured at 37 ^o^C, 5% CO_2_ for 24 h. Then 10 μM of the test compounds were added and incubated with HCT116 cells for another 72 h, followed by a centrifugation to collect the medium. Subsequently, the ethyl acetate was used to extract the corresponding compound, and then analyzed by LC-MS/MS.

*Cellular Thermal Shift Assay*

Cellular thermal shift assay for compound **2** was performed according to the reported protocol.[1] The HCT116 cells were treated with compound **2** (4 μM) or DMSO for 4 h and then were harvested with PBS. Then the samples were lysed using liquid nitrogen and three repeated cycles of freeze-thaw. Subsequently, the cell suspension was aliquoted into ten PCR tubes and heated for 5 minutes to 49, 51, 53, 55, 57, 59, 61, 63, 65 or 68 °C. After a centrifugation at 12,000 rpm for 15 min at 4 °C, the supernatant was collected for further western blotting analysis.

*Colony formation assay*

HCT116 cells were seeded in the 6-well plates at a low density (2000 cells per well) in 2 mL culture media and cultured in an incubator with 5 % CO_2_ at 37 °C for 24 h. The cells were incubated with test compound at indicated concentrations to proliferation for one week. Afterwards, cells were washed with PBS, fixed with 4% paraformaldehyde for 15 min, and stained with 1% crystal violet dye for 15 min, and then images of each well were taken *via* digital camera.

*Wound-Healing Assay*

Briefly, HCT116 cells were seeded into 6-well plates with a density of 5 × 10^5^ cells/well and cultured until a confluent monolayer was formed. A scratch on the monolayer cells was generated by using a sterilized 200 μL pipette tip, and then the PBS was added gently into each well to wash the cells. Further incubation with various concentrations of compound **13c** were performed. The closure of the “wound” was observed under the microscope, and the images of each well at 0 and 24 h were captured using Nikon Digital Sight 10.

*Cell Invasion Assay*

In the transwell experiment, HCT116 cells (2 × 10^5^/mL) were seeded in upper transwell chambers after serum starvation for 12 h. A total of 600 μL of a DMEM medium involving 20% FBS was added to the bottom chamber. Immediately, diverse concentrations of the test compounds were added to the upper chamber and cultivated for 48 h. After that, cells on the upper chamber surface were lightly wiped, and cells migrated to the lower chamber were immobilized with 4% paraformaldehyde, followed by 0.1% crystal violet staining. The images were taken using a microscope.

*Flow cytometer analysis of cell cycle*

HCT116 cells were plated in six-well plates at a density of 2 × 10^5^ cells/well and treated with various concentrations of the test compounds for 48 h. And then the HCT116 cells were collected and washed with cold PBS. After centrifugation, the precipitate was resuspended in 500 μL of DNA staining solution, and then 5 μL of Permeabilization solution were added for an additional incubation (15 min) in the dark at room temperature. The samples were then analyzed by ﬂow cytometry (BD Accuri C6).

*In Vivo Antitumor Experiment*

The *in vivo* antitumor activities of compounds **2** and **13c** at a dose of 10 mg/kg were estimated. The nude mice xenograft model was prepared by injecting 5 × 10^6^ HCT116 cells (in 200 μL PBS) subcutaneously into the right axilla region of the BALB/C nude female mice. The animals were randomized to different groups (five or six mice in each group) when the tumor volume reached around 100 mm^3^, and then the test compounds were administrated by intraperitoneal injection twice per day for 14 consecutive days. The vehicle mice were administered by IP injection with saline to serve as a control. Tumor volumes were determined by caliper measurement of the length and width and calculated using the formula TV = 1/2 × *a* × *b*^2^, where *a* is the tumor length and *b* is the width. The tumor volumes and body weights were recorded every 2 days during the treatment period. At the end of the experiment, the mice were euthanized, and the tumors were removed and analyzed. The H&E staining, Ki67 staining and Tunel staining of tumor tissue were performed by Shanghai Shycbio Biological Technology Co.Ltd.

*In Vivo Western Blotting Analysis*

The segregated tumor tissues were cut into pieces and lysed with RIPA buffer containing phosphatase inhibitor (EpiZyme #GRF102) and protease inhibitors (EpiZyme #GRF101) by high throughput tissue grinder to extract the total protein, and the lysates were centrifuged at 12,000 rpm at 4 °C for 15 min. The cell supernatant was collected and determined the protein concentration with the BCA kit. Equal amounts of protein (30 μg) were analyzed using SDS-PAGE gels, and then the protein was transferred to polyvinylidene fluoride membranes. The membrane was then blocked with 5% BSA for 2 h at room temperature and incubated with primary antibodies against REXO4 (Proteintech, 18890-1-AP, 1:1,000) and anti-GAPDH (Abcam #ab181602, 1:10,000) overnight at 4 °C. After washing with TBST for three times, the membrane was incubated with fluorescent secondary antibodies for 2 h and analyzed using a LI-COR Odyssey imaging system.

*REXO4 KD HCT116 cell line*

The REXO4 KD HCT116 cell line (HCT116 cells that the REXO4 gene was knocked down) was provided by Genomeditech (Shanghai, China).

**
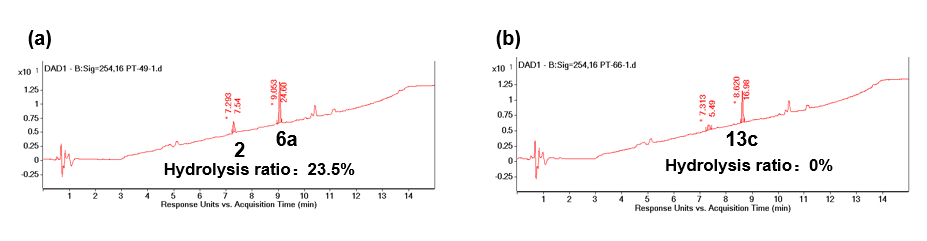
**

**Figure S1.** The stability test of compounds **6a** and **13c**. Compounds **6a** and **13c** at a concentration of 10 μM was incubated with HCT116 cells, respectively, and then was detected by LC-MS/MS.


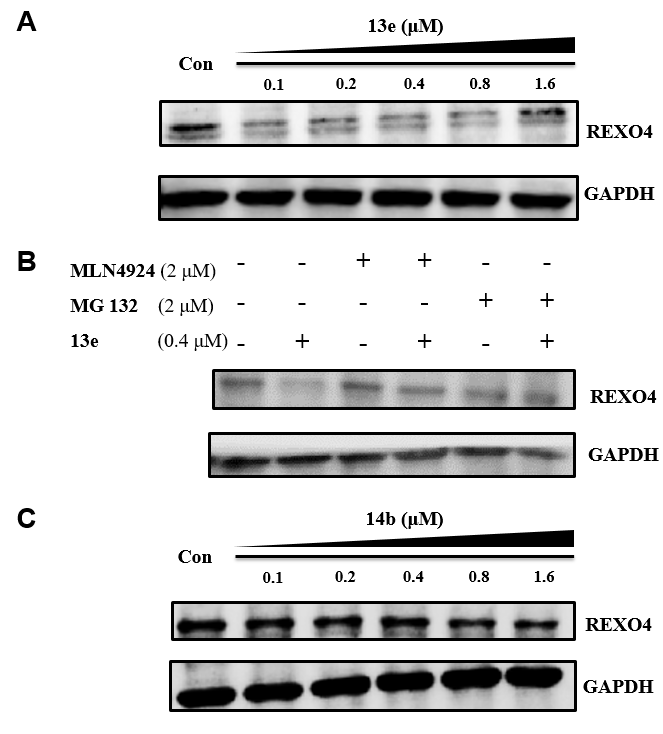


**Figure S2.** EVO-PROTAC **13e** induce the REXO4 protein degradation through the UPS. The expression level of REXO4 protein in HCT116 cells after the treatment of EVO-PROTAC **13e** (A) and negative control **14b** (C) for 24 h. (B) The expression level of REXO4 protein after the pretreatment with proteasome inhibitor MG132 (2 μM) or the neddylation inhibitor MLN4924 (2 μM) for 2 h before the incubation with either vehicle (DMSO) or EVO-PROTAC **13e** (0.4 μM) for 24 h.

# Representative ^1^H-NMR, ^13^C-NMR and ESI-MS of EVO-PROTACs

^1^H NMR (600 MHz) ^13^C NMR (150 MHz) and ESI-MS of compound **6a** in DMSO-*d_6_*


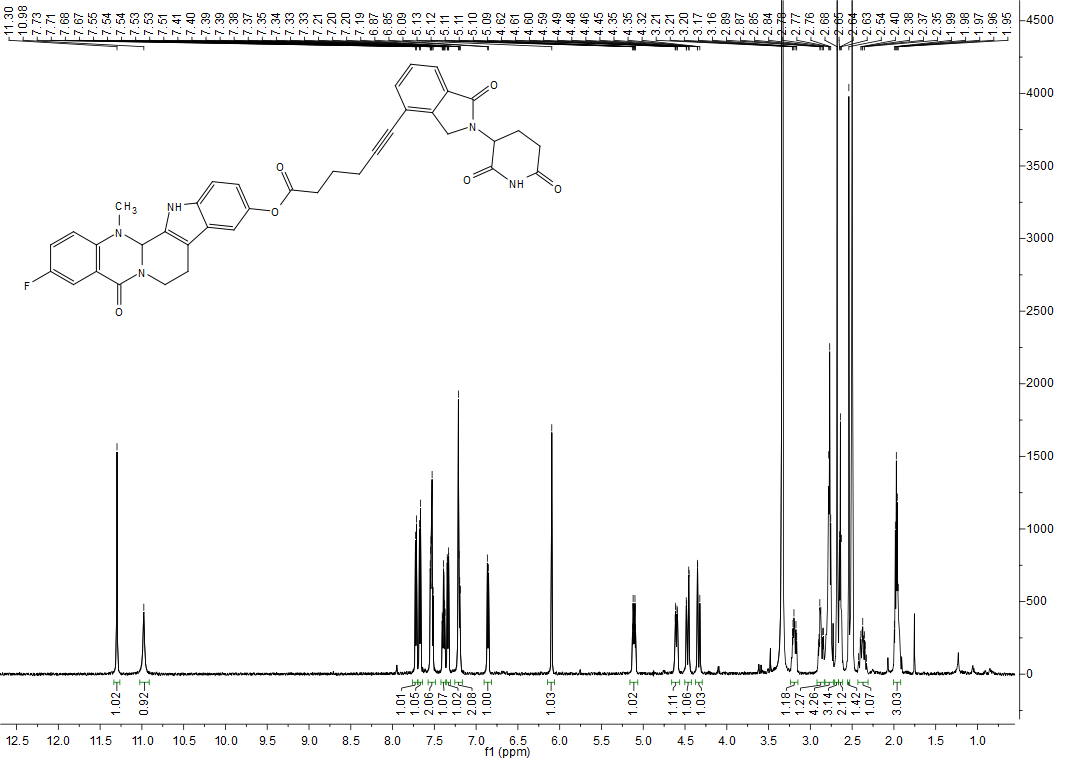


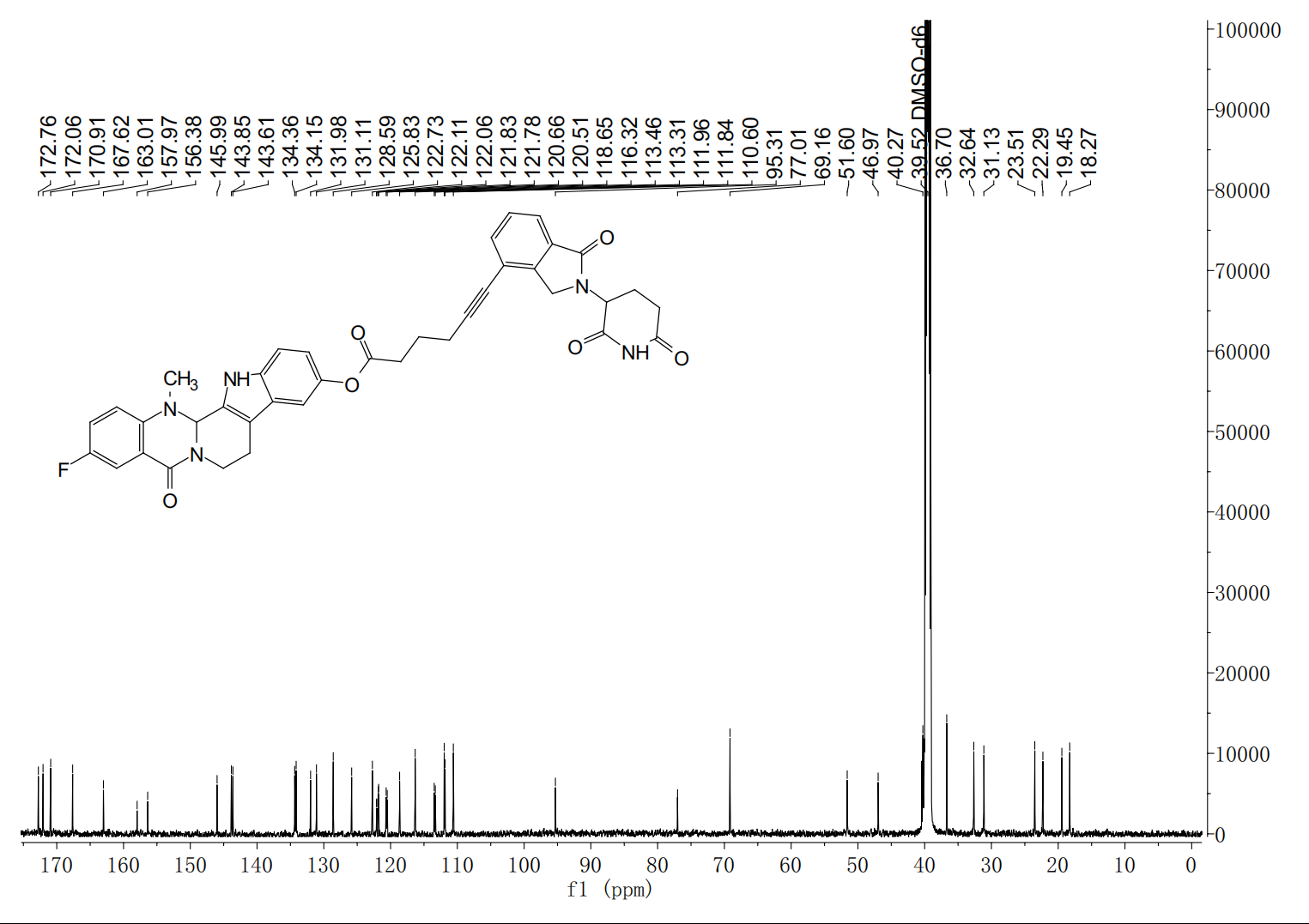


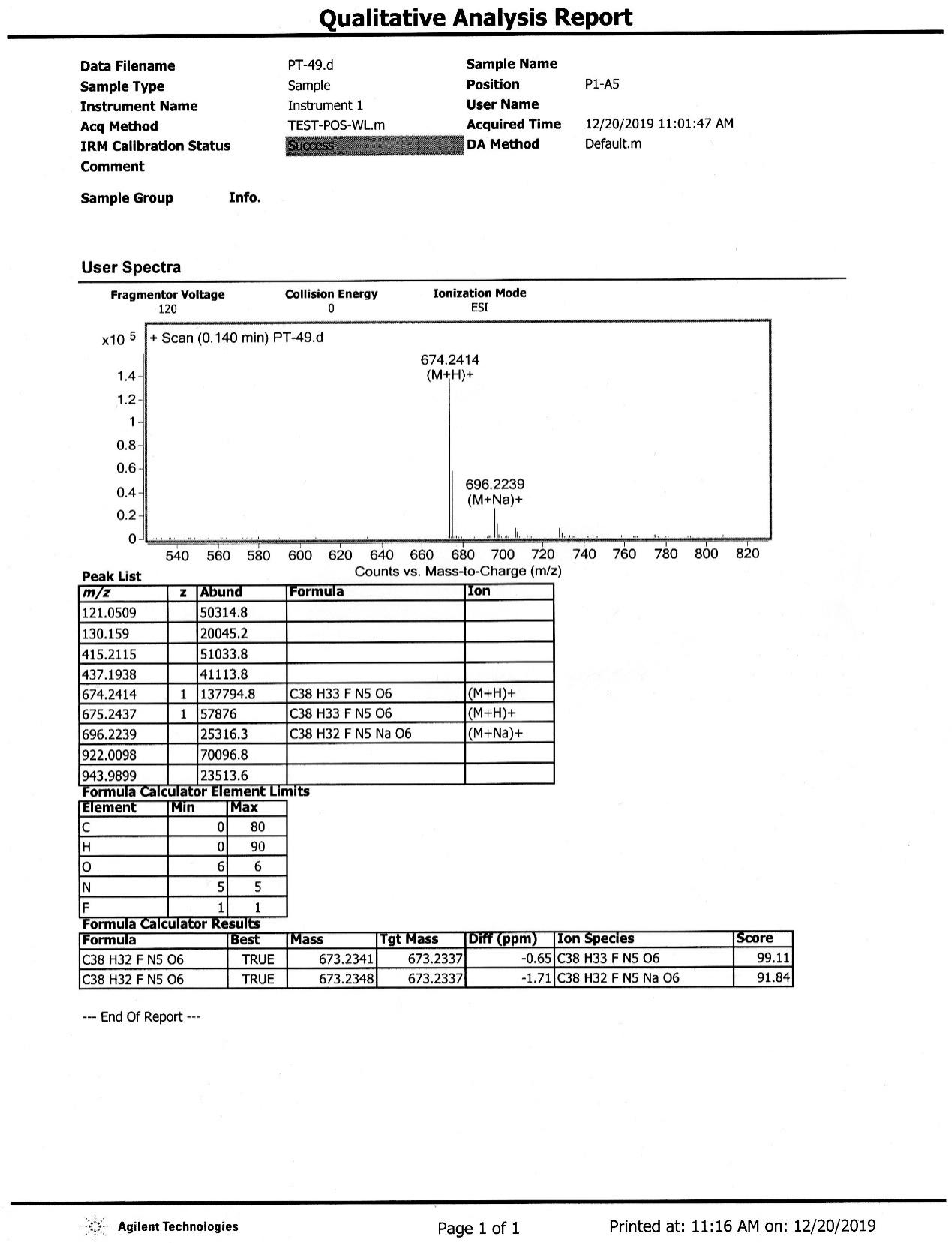


^1^H NMR (600 MHz) ^13^C NMR (150 MHz) and ESI-MS of compound **6b** in DMSO-*d_6_*


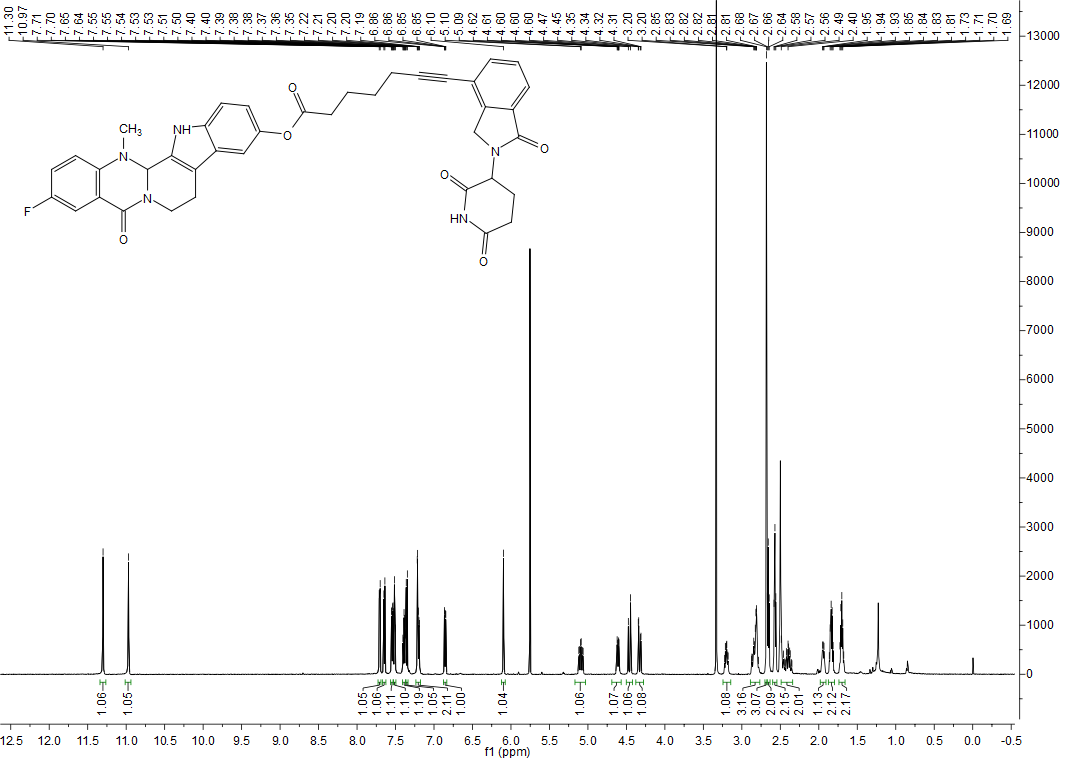


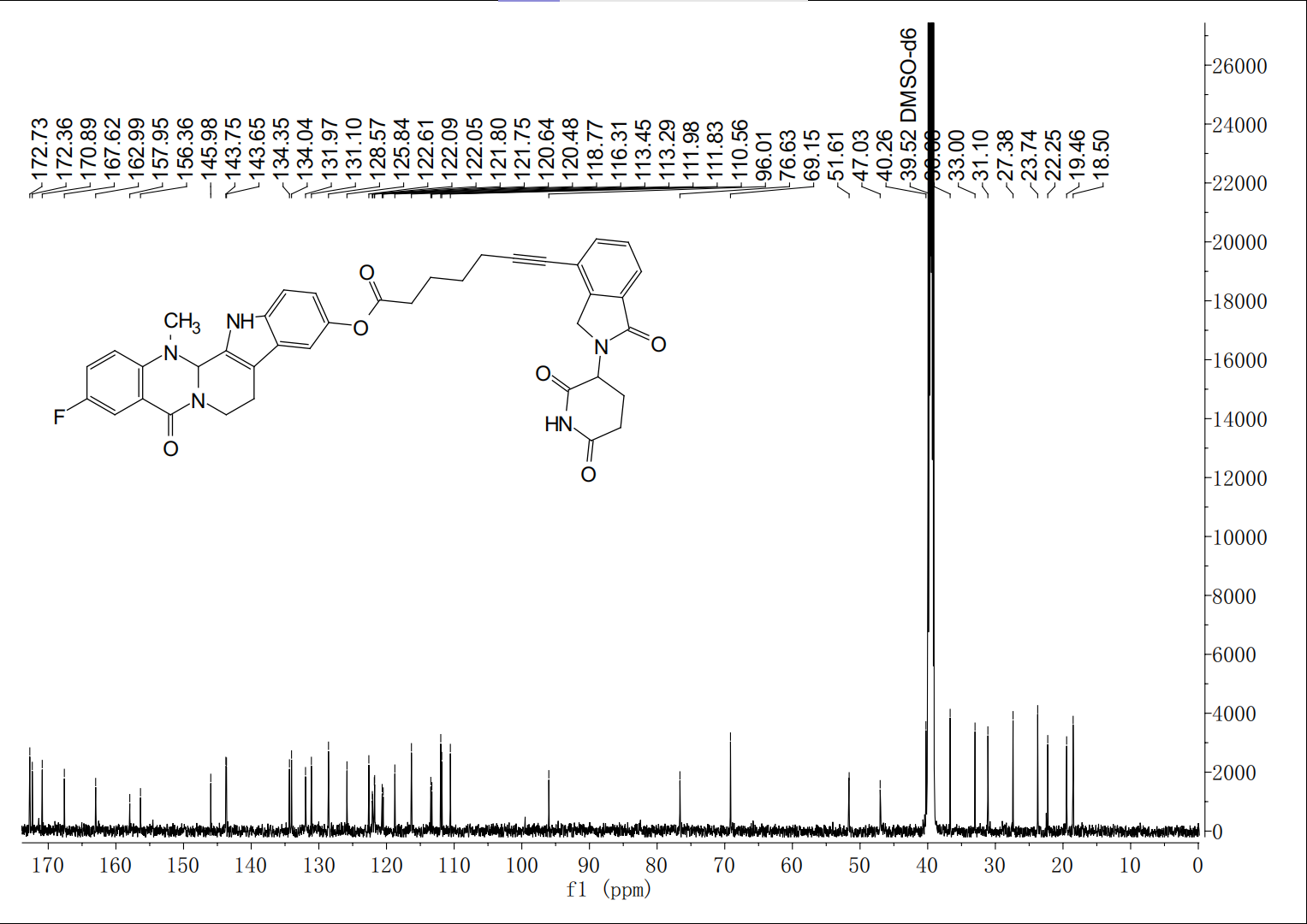


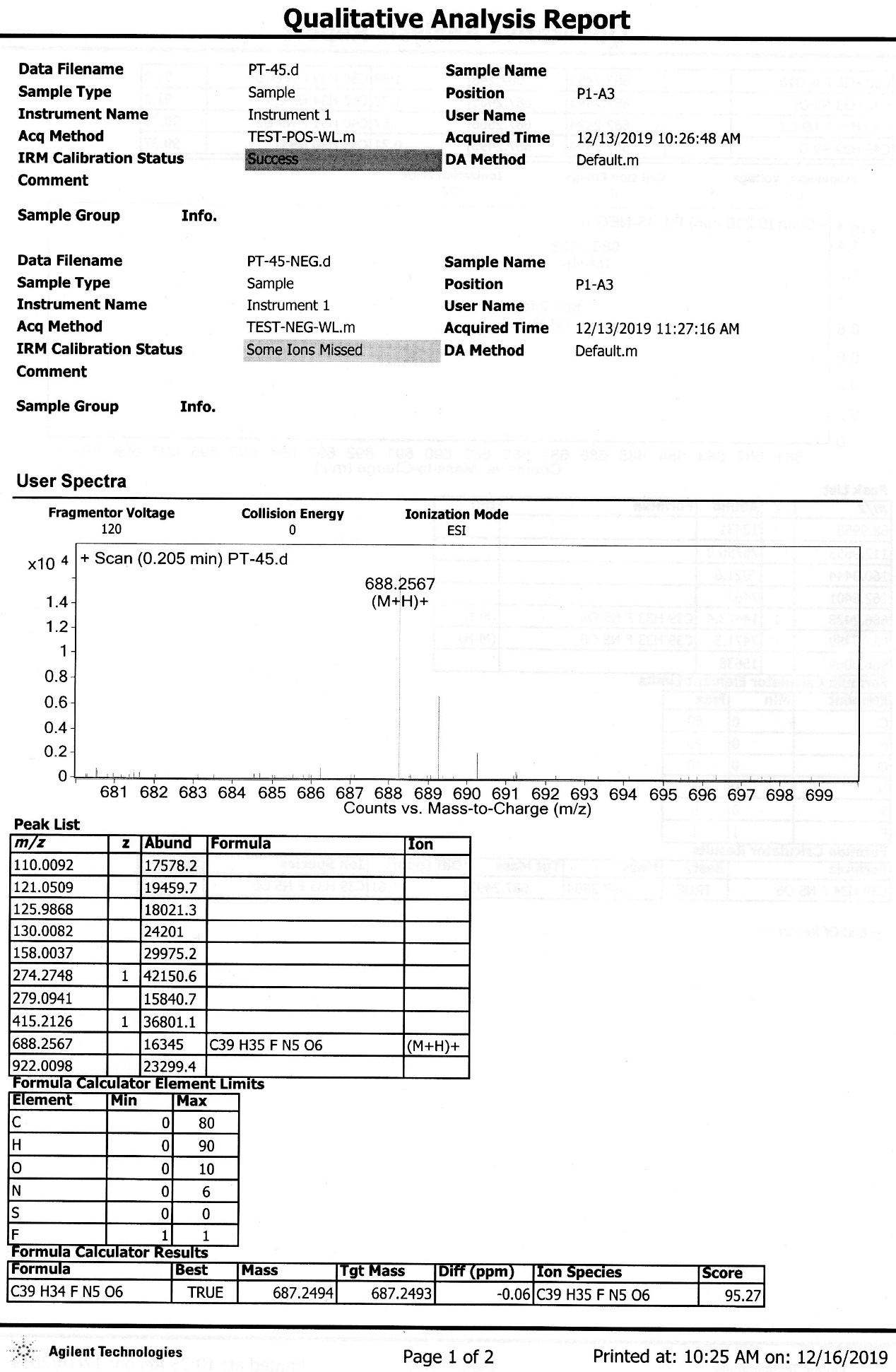


^1^H NMR (600 MHz) ^13^C NMR (150 MHz) of compound **6c** in DMSO-*d_6_*


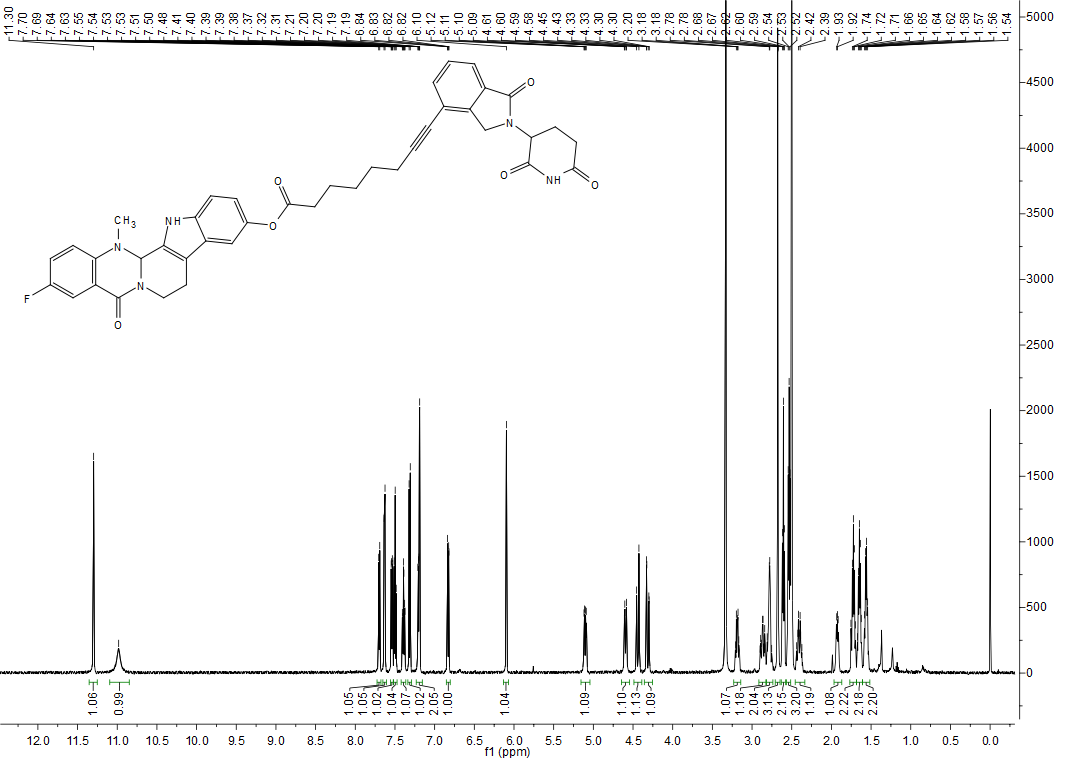


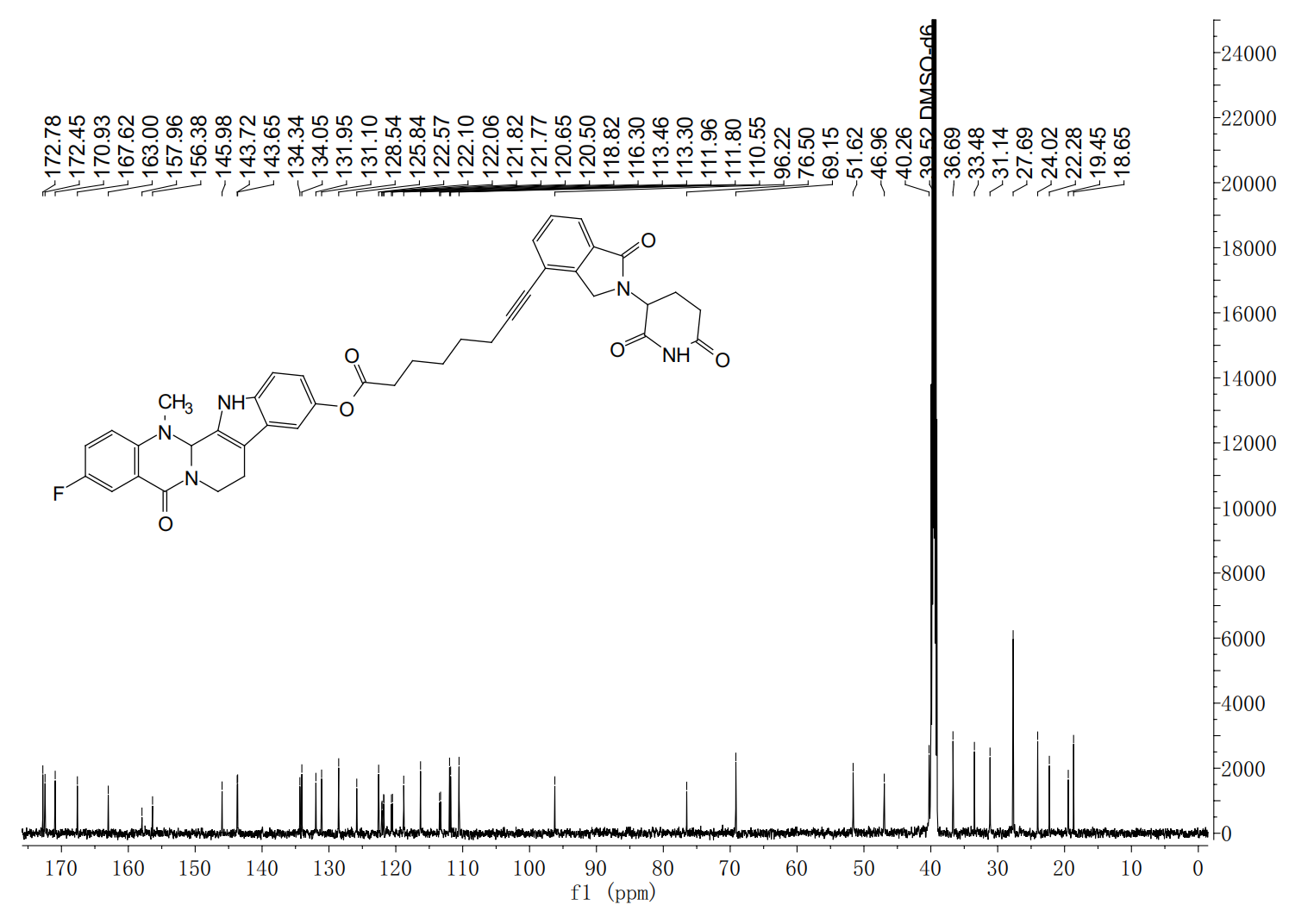


^1^H NMR (600 MHz) ^13^C NMR (150 MHz) and ESI-MS of compound **6d** in DMSO-*d_6_*


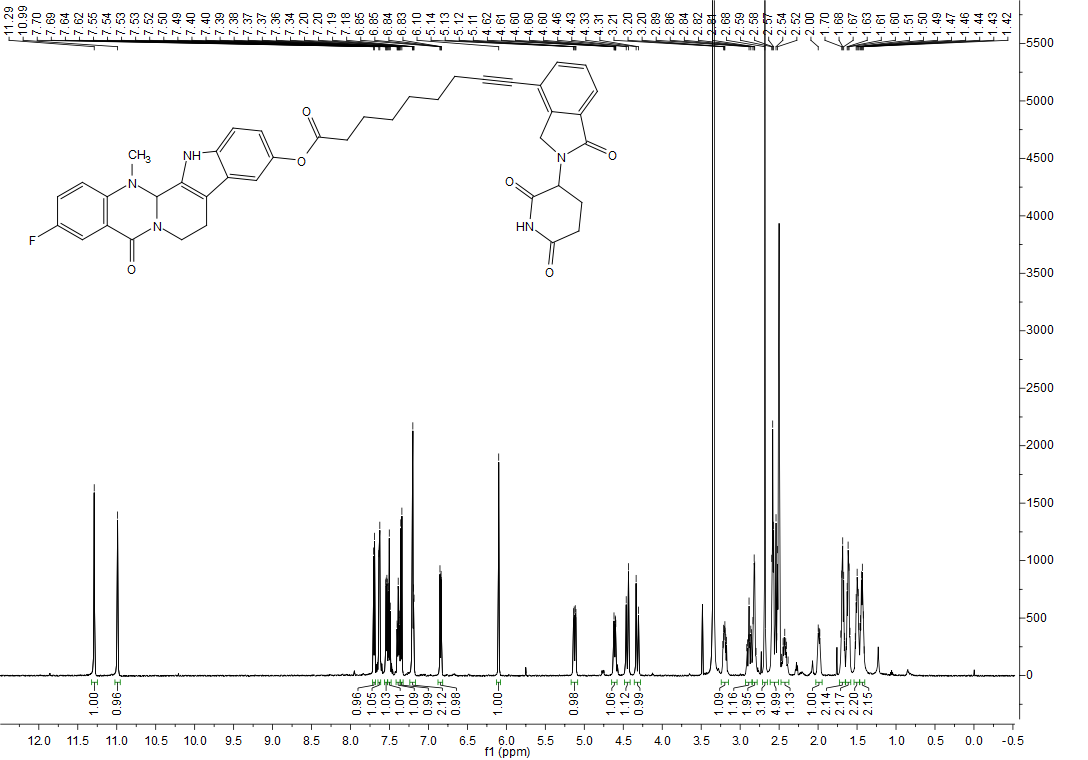


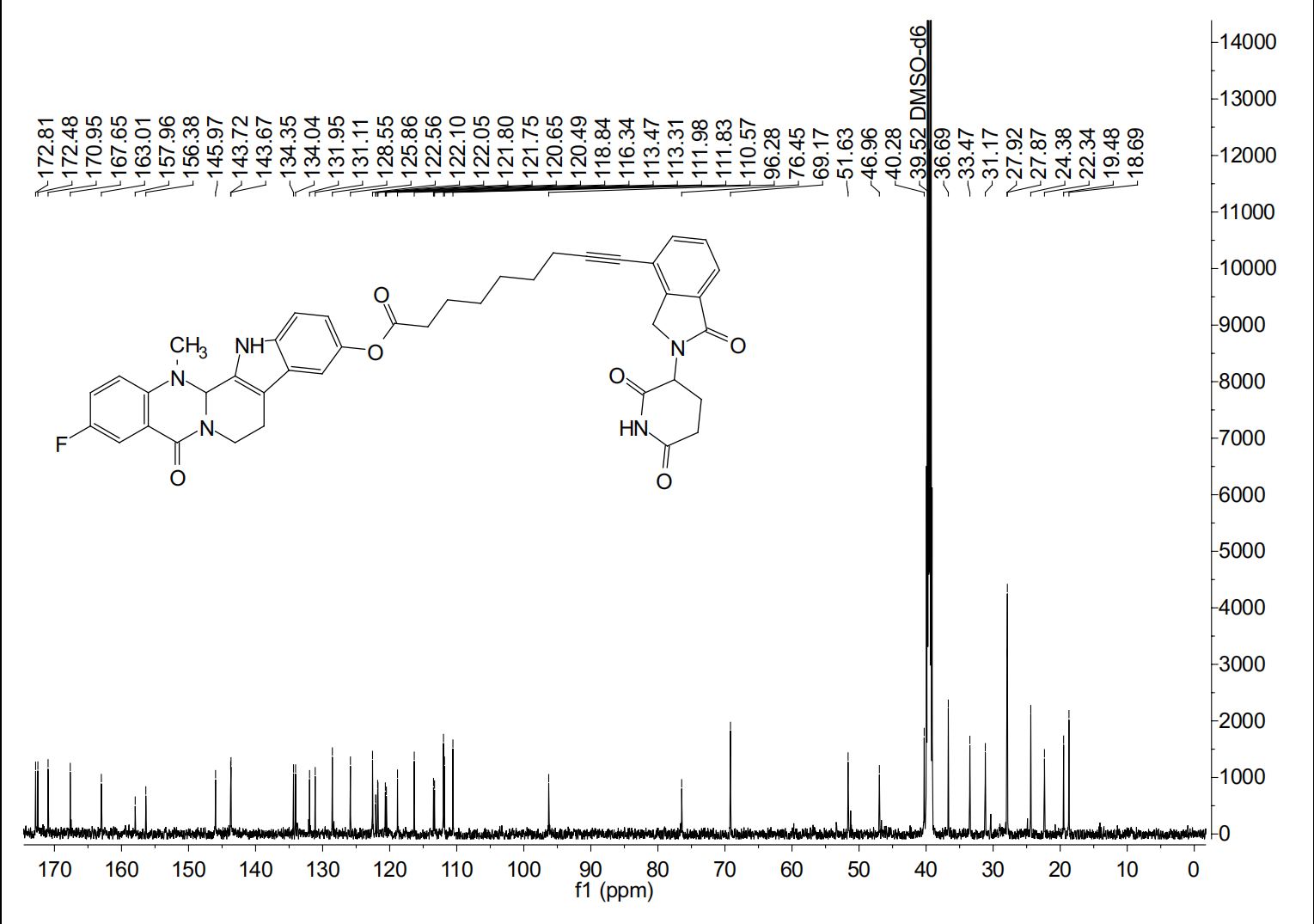


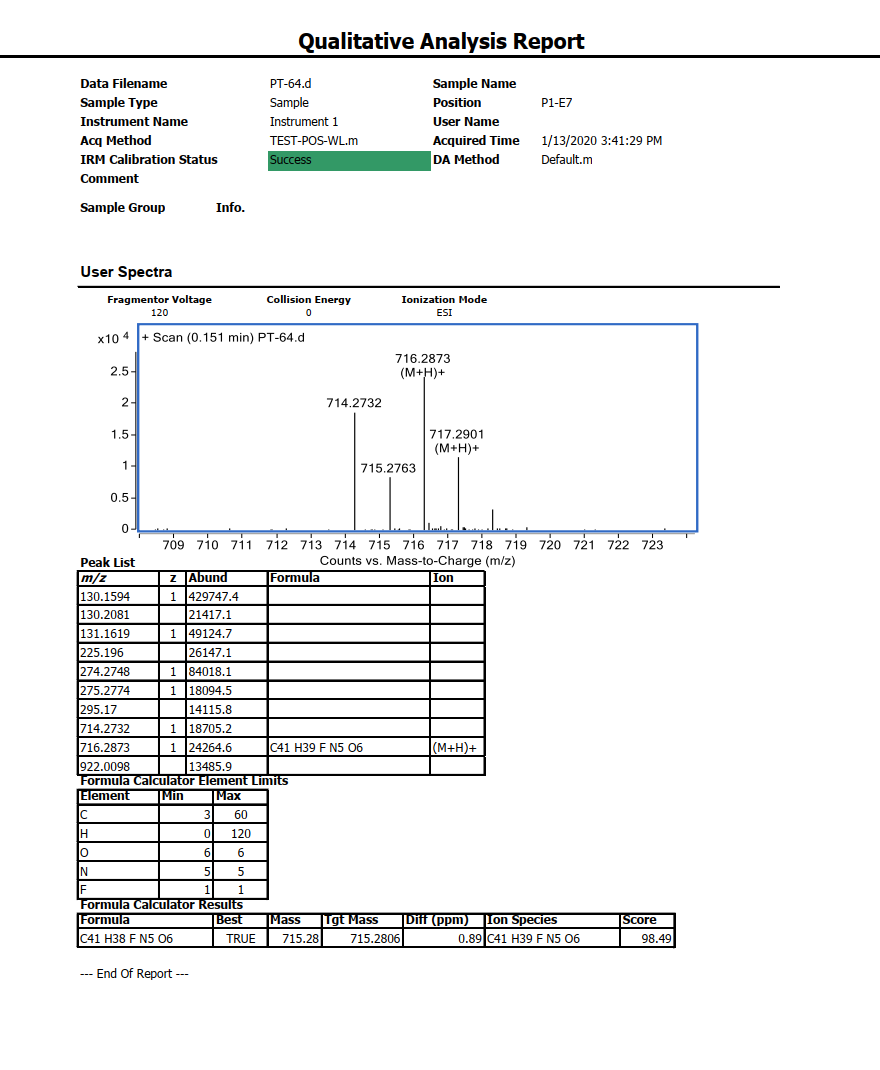


^1^H NMR (600 MHz) ^13^C NMR (150 MHz) and ESI-MS of compound **6e** in DMSO-*d_6_*


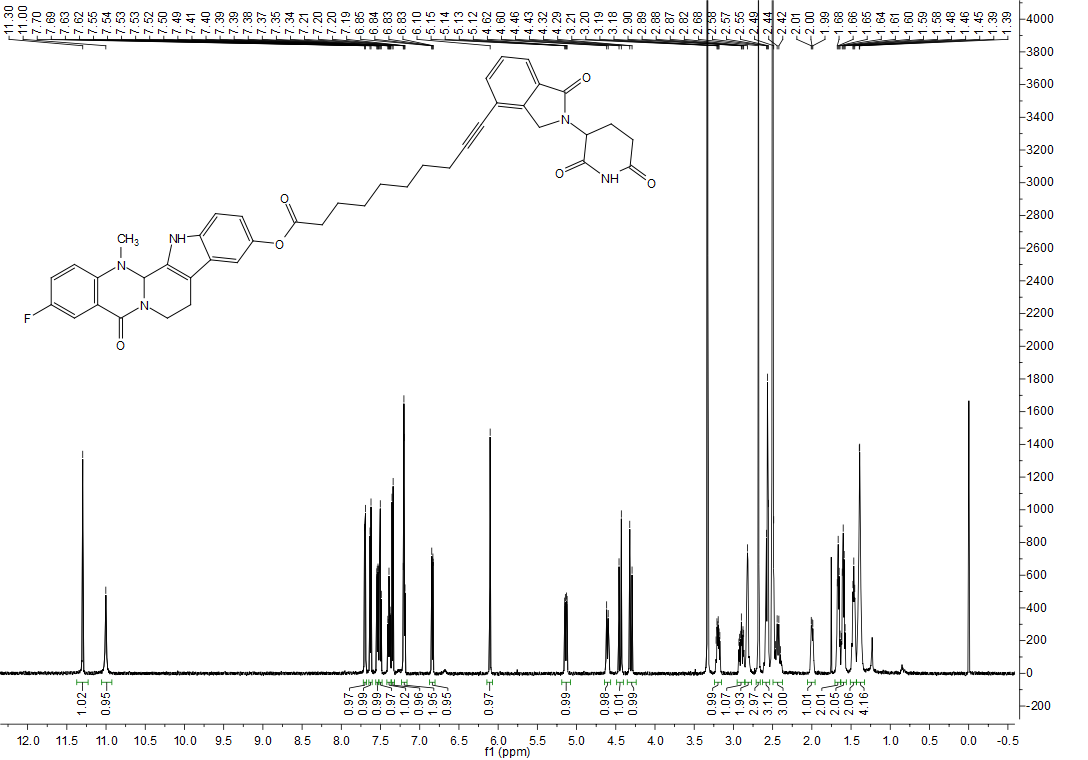


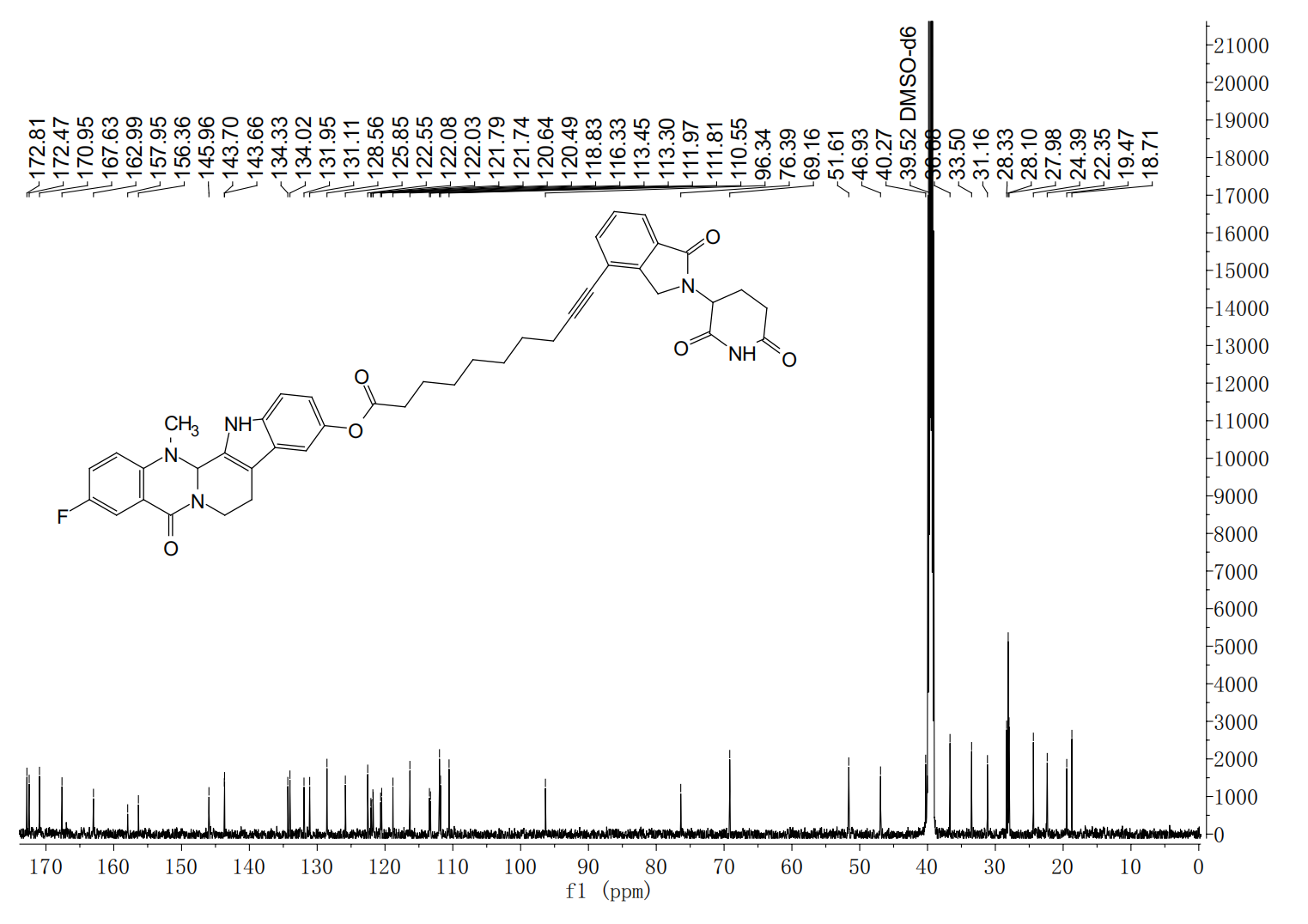


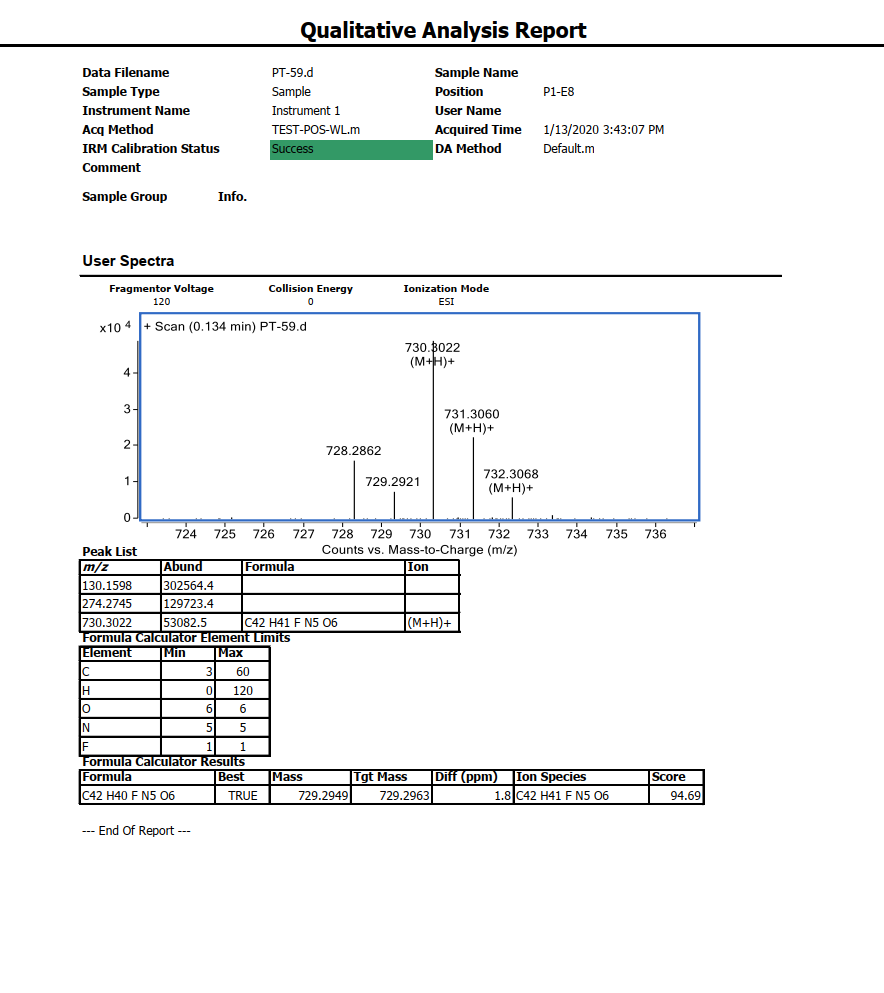


^1^H NMR (600 MHz) ^13^C NMR (150 MHz) and ESI-MS of compound **13a** in DMSO-*d_6_*


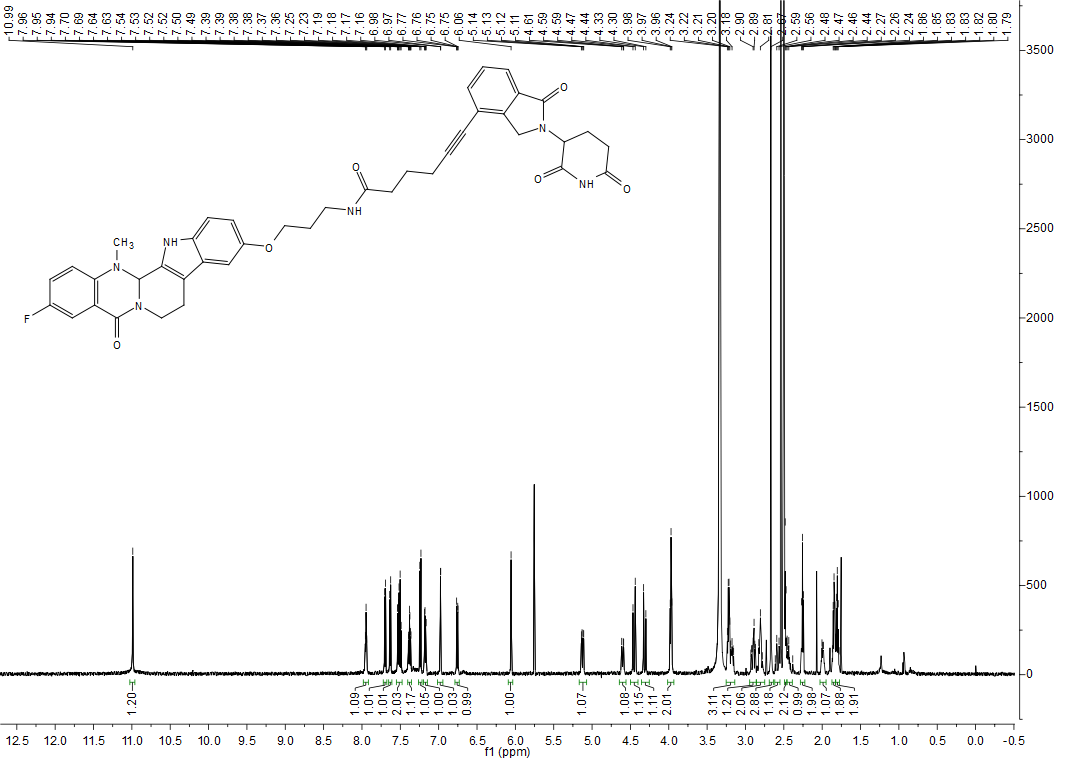


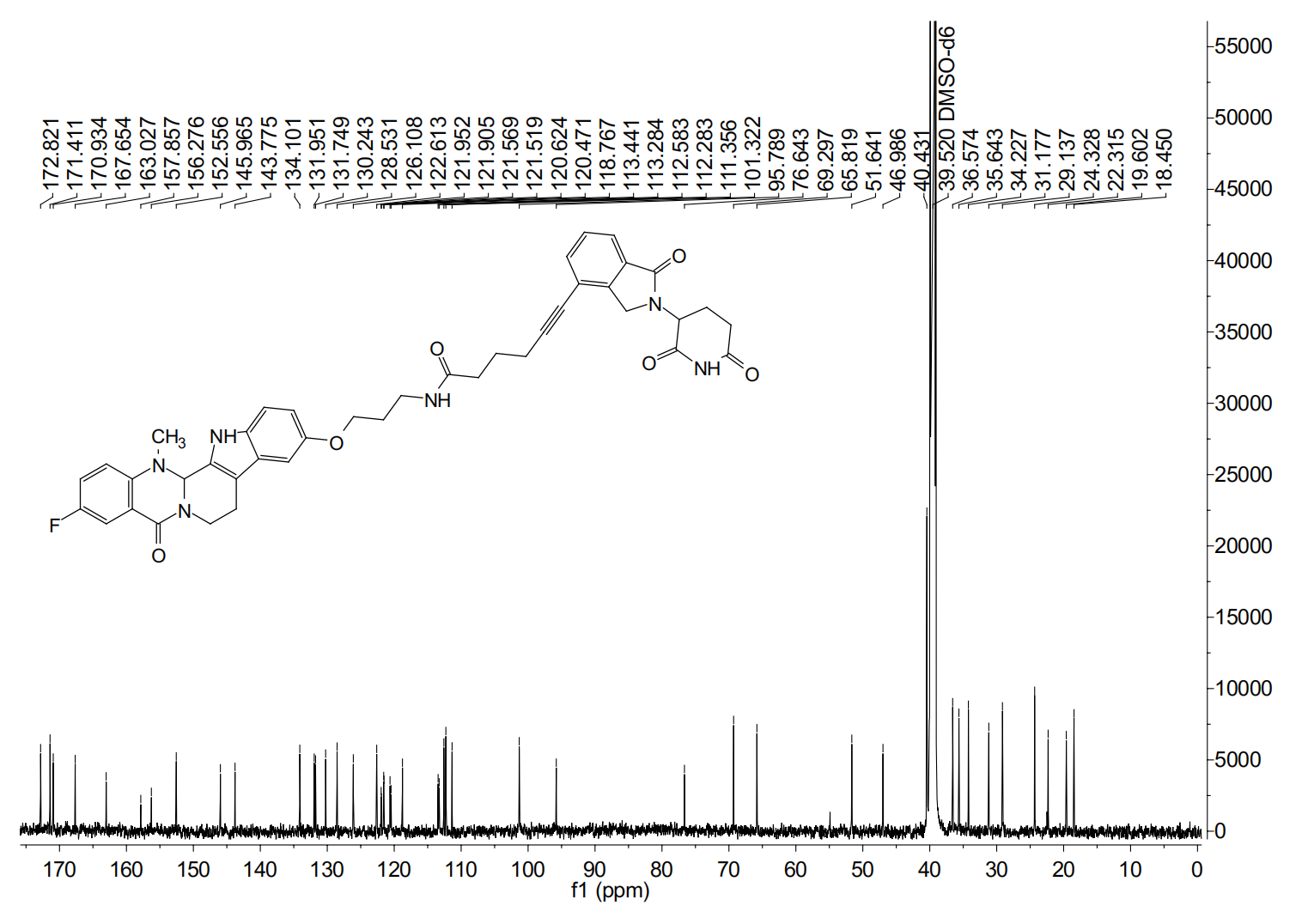


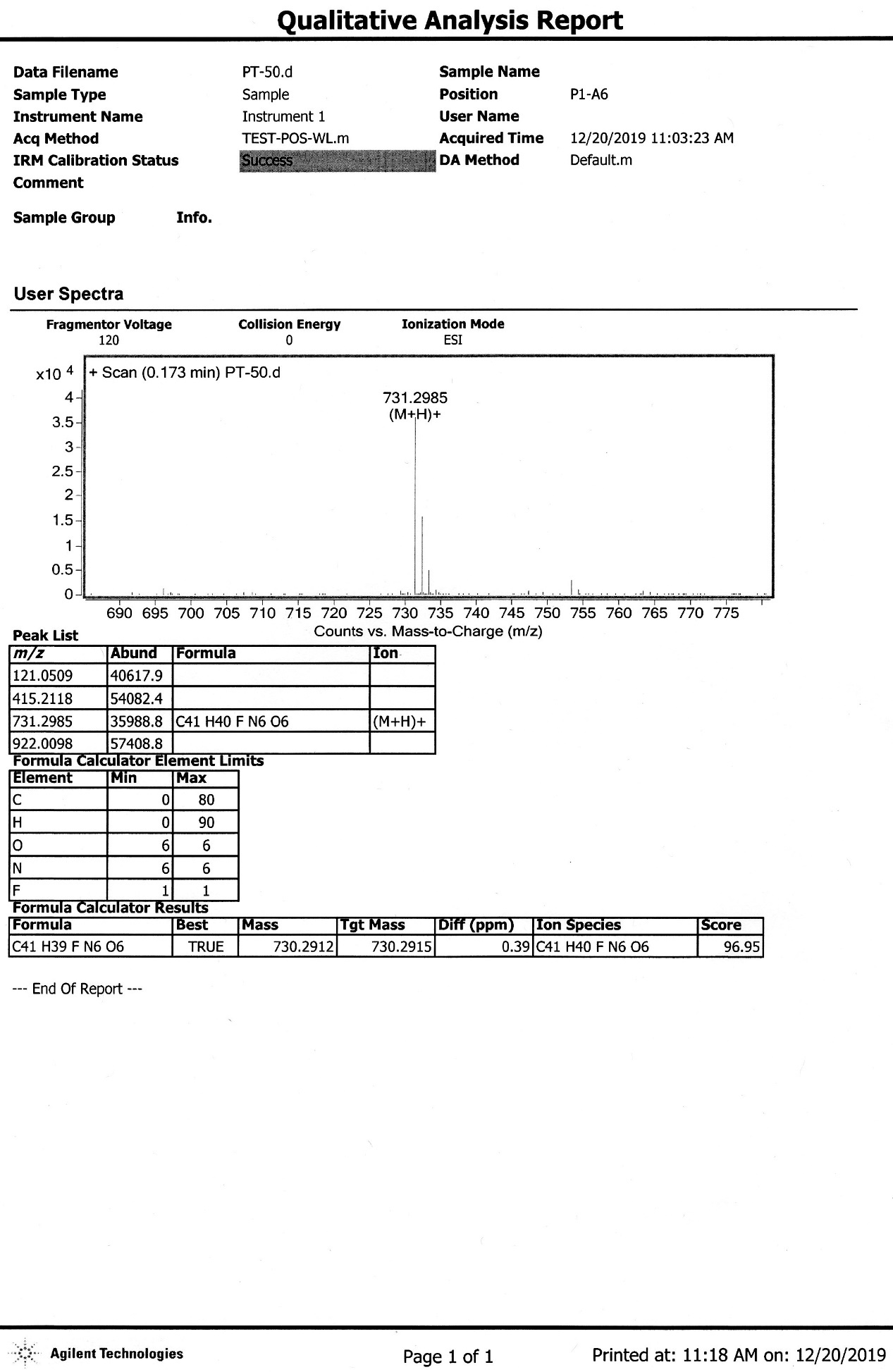


^1^H NMR (600 MHz) ^13^C NMR (150 MHz) and ESI-MS of compound **13b** in DMSO-*d_6_*


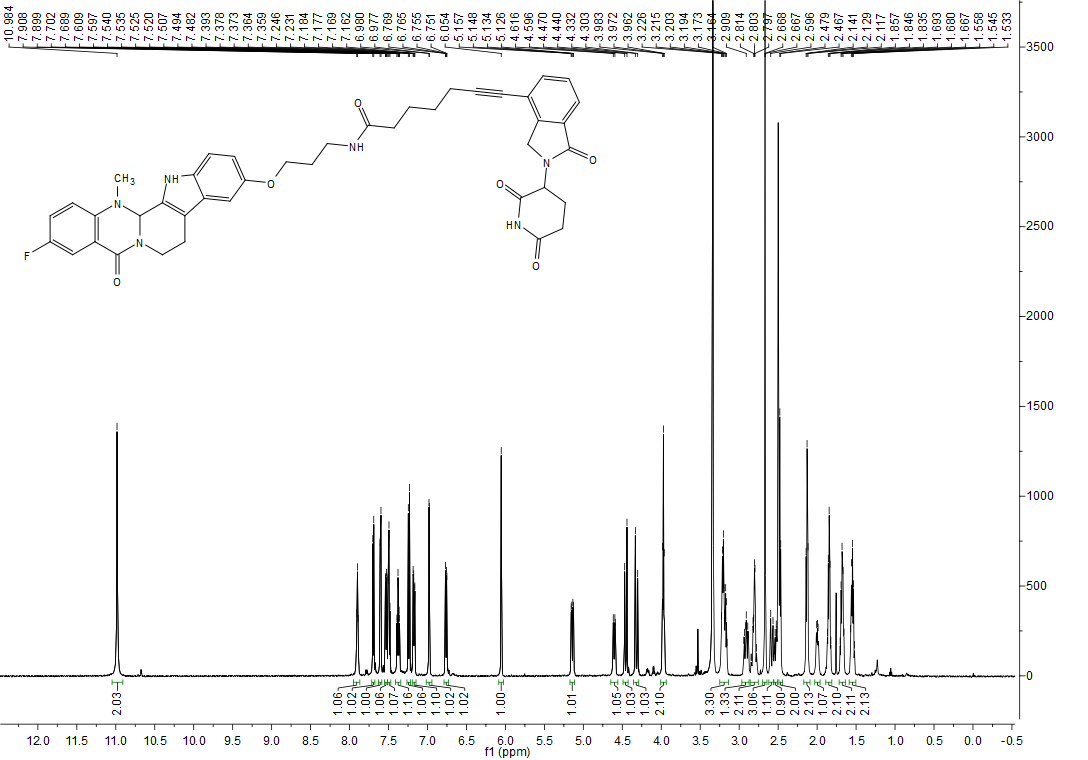


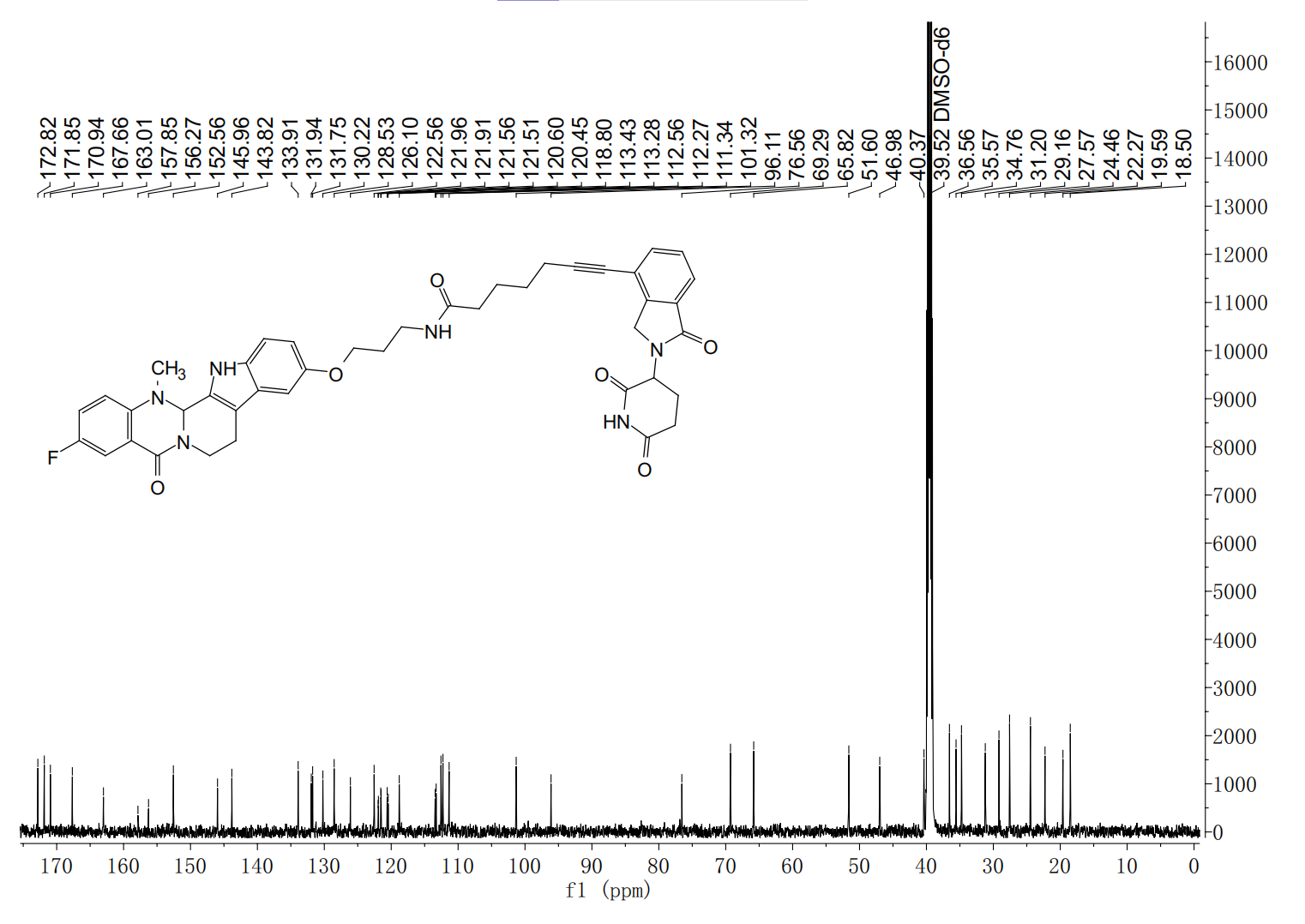


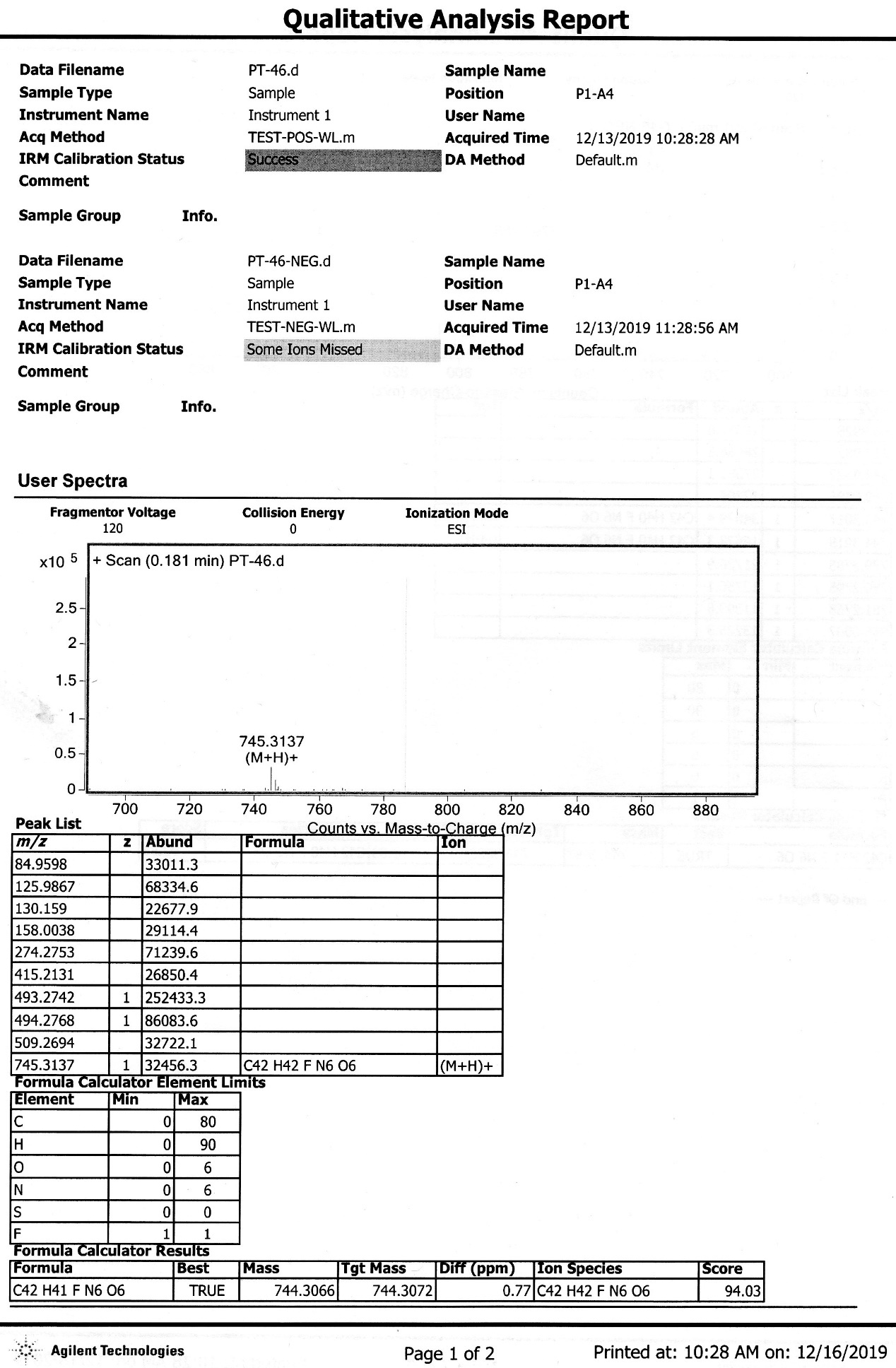


^1^H NMR (600 MHz) ^13^C NMR (150 MHz) and ESI-MS of compound **13c** in DMSO-*d_6_*


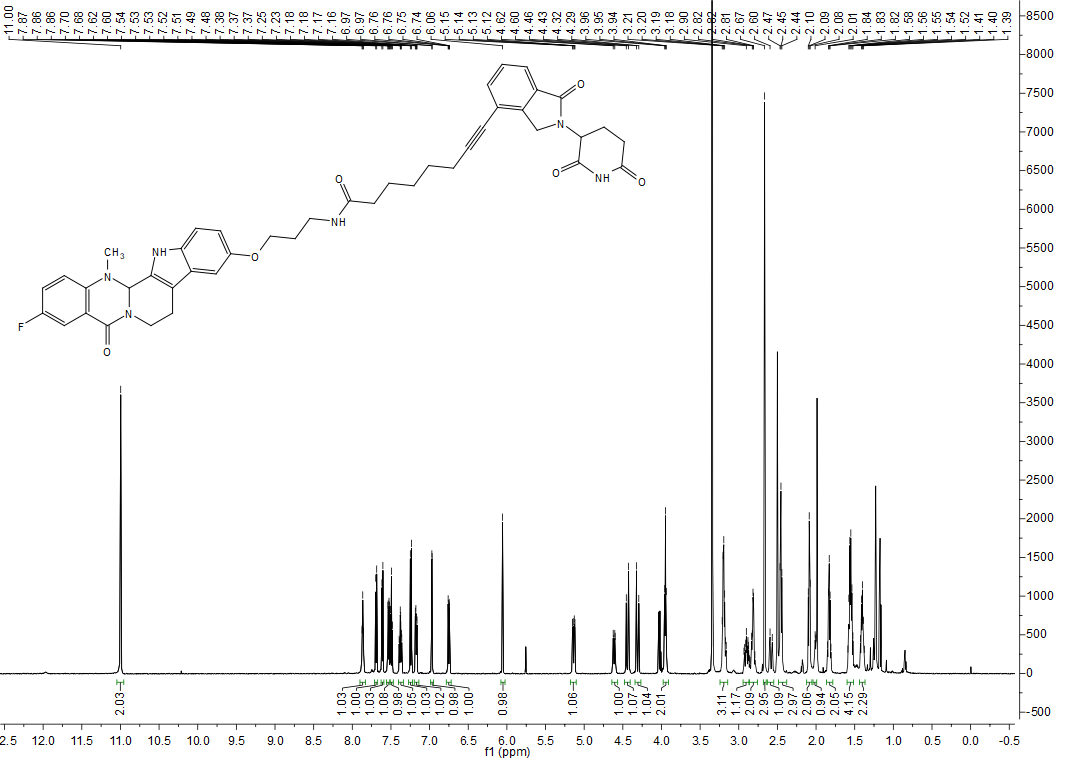


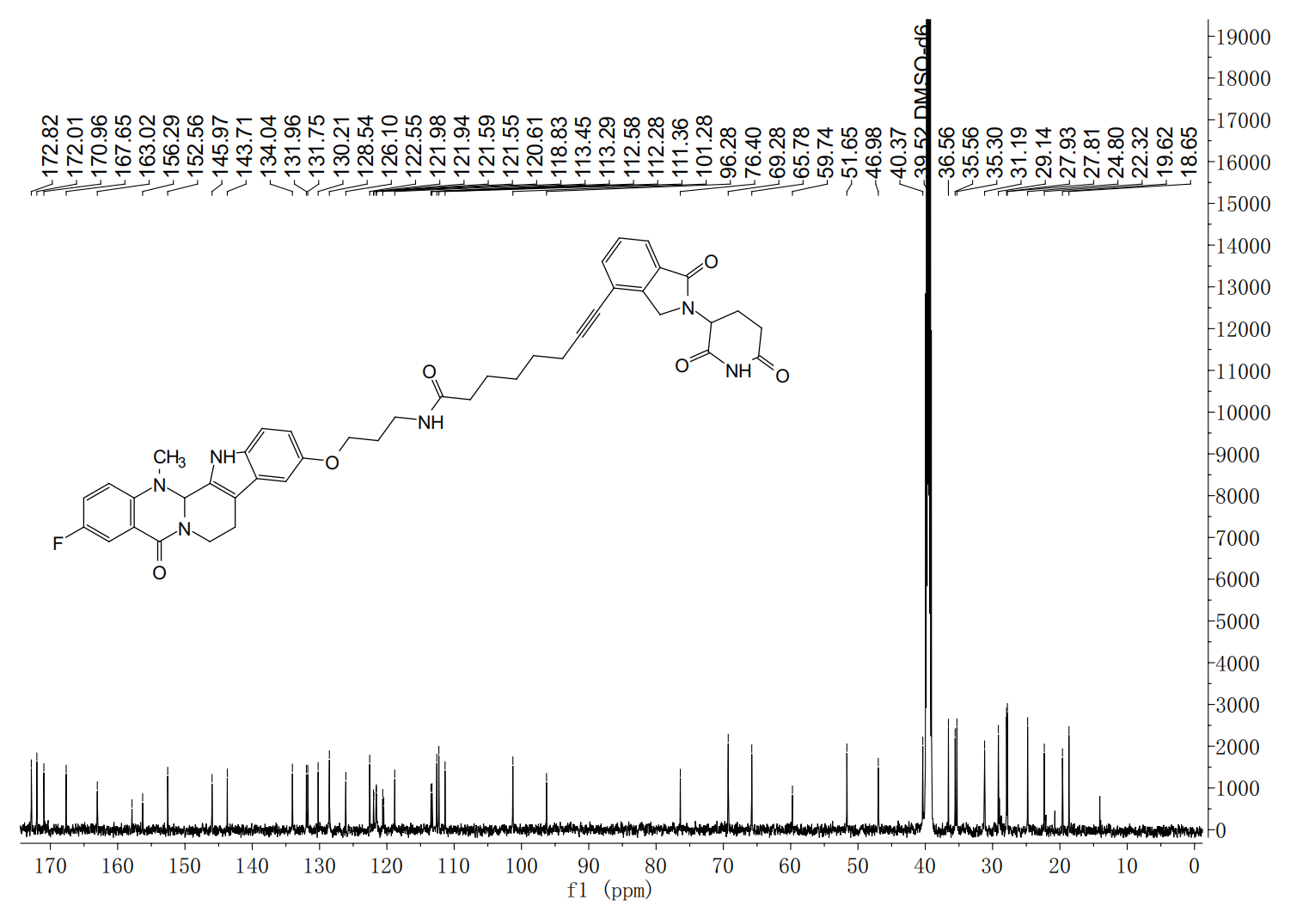


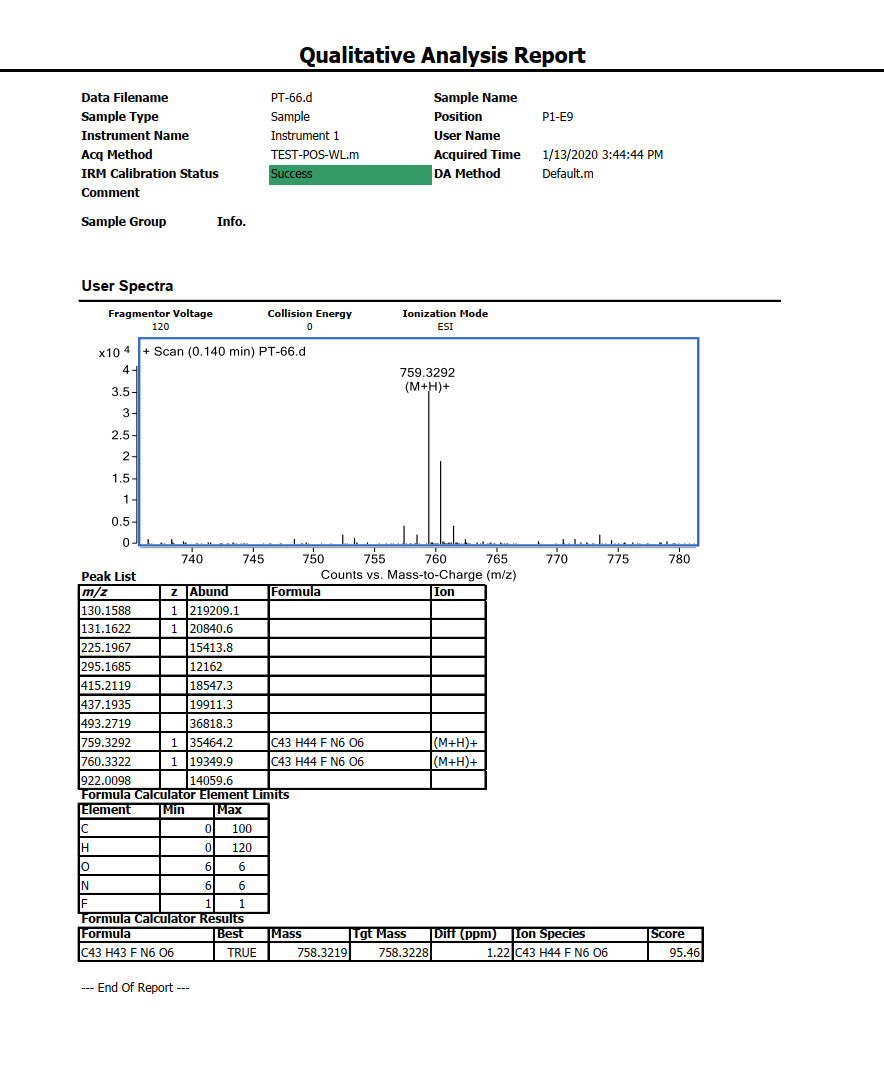


^1^H NMR (600 MHz) ^13^C NMR (150 MHz) and ESI-MS of compound **13d** in DMSO-*d_6_*


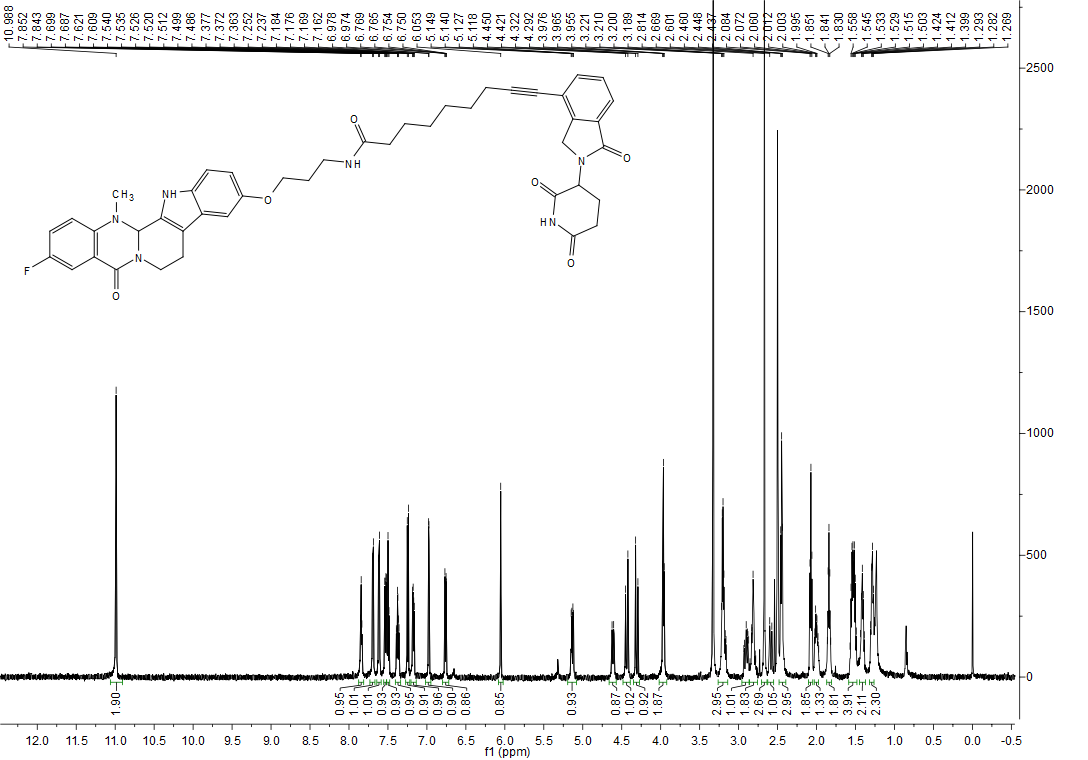


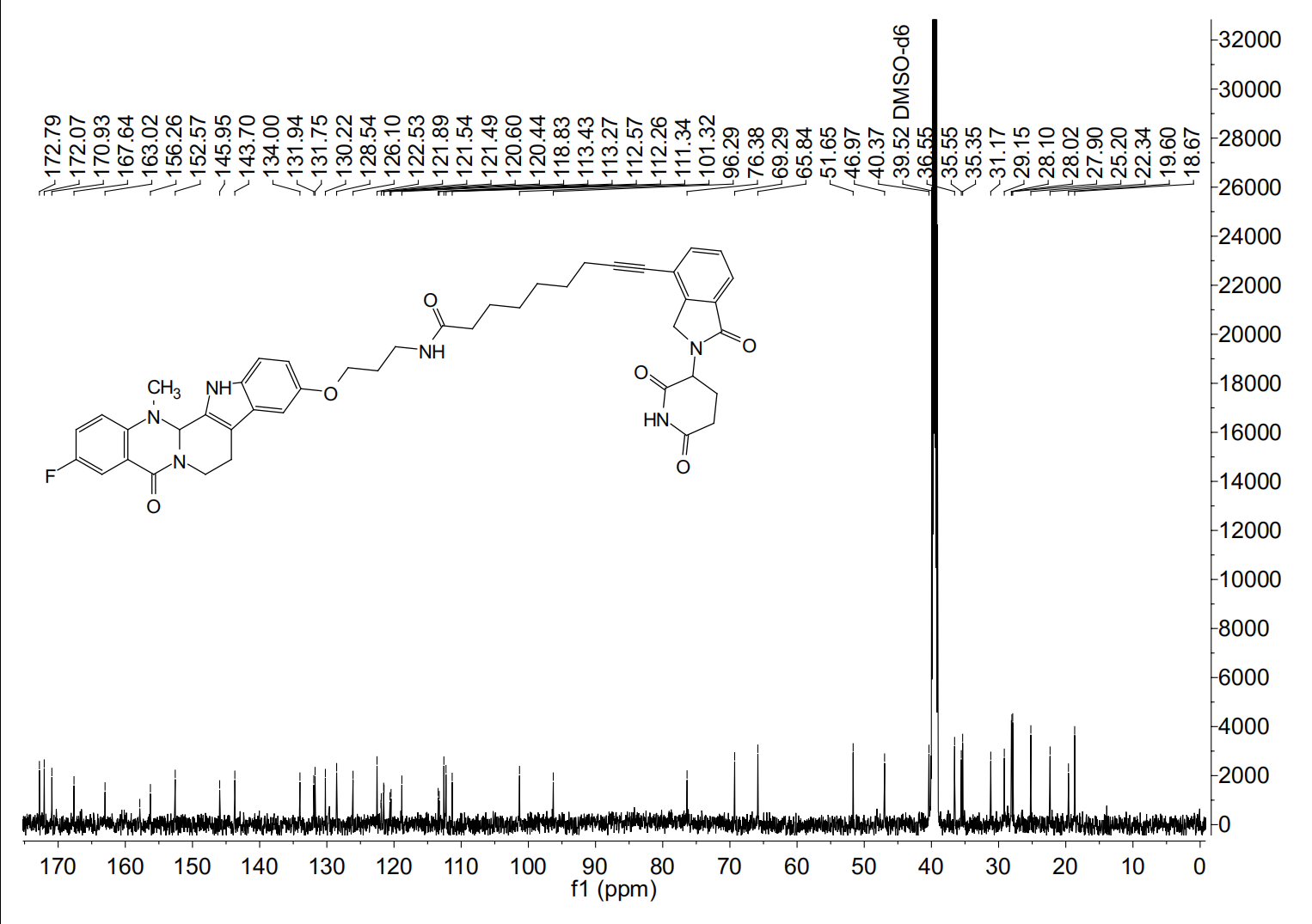


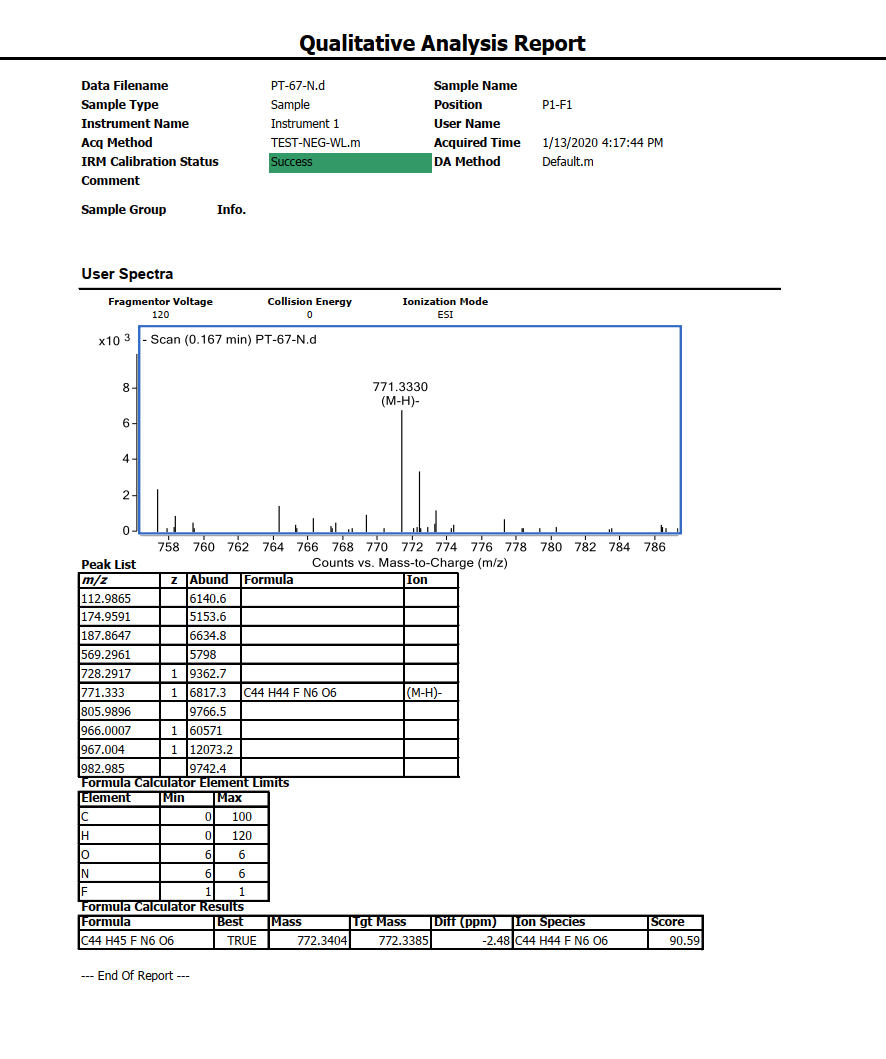


^1^H NMR (600 MHz) ^13^C NMR (150 MHz) and ESI-MS of compound **13e** in DMSO-*d_6_*


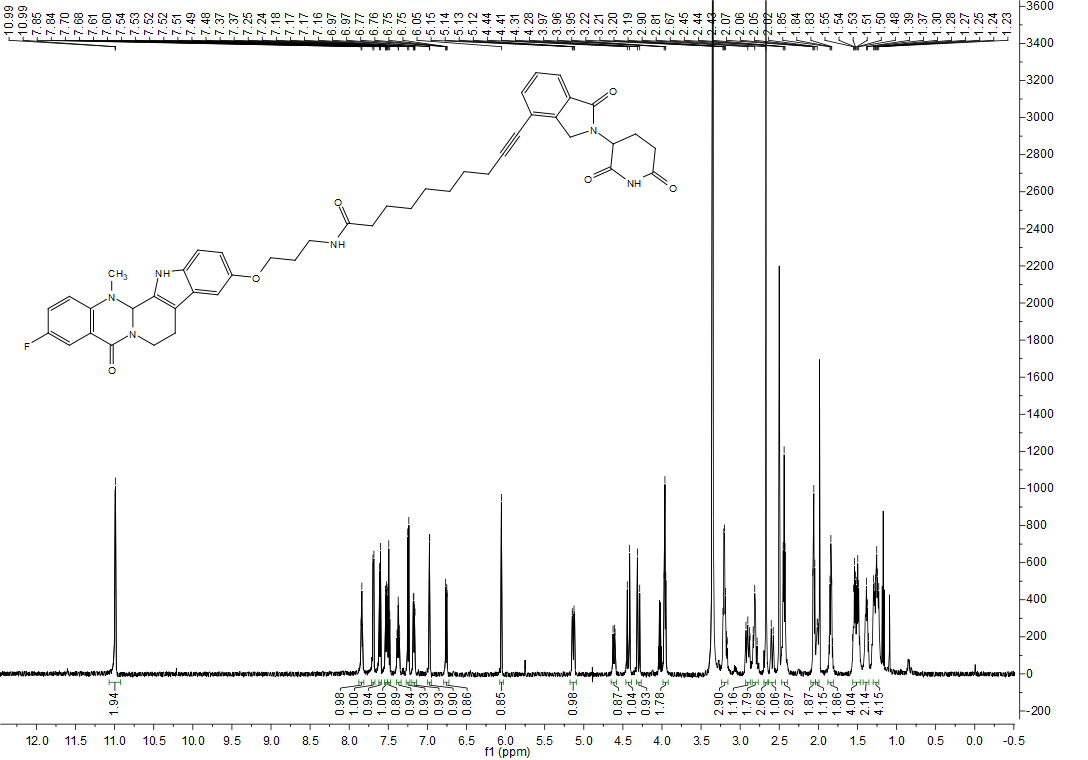


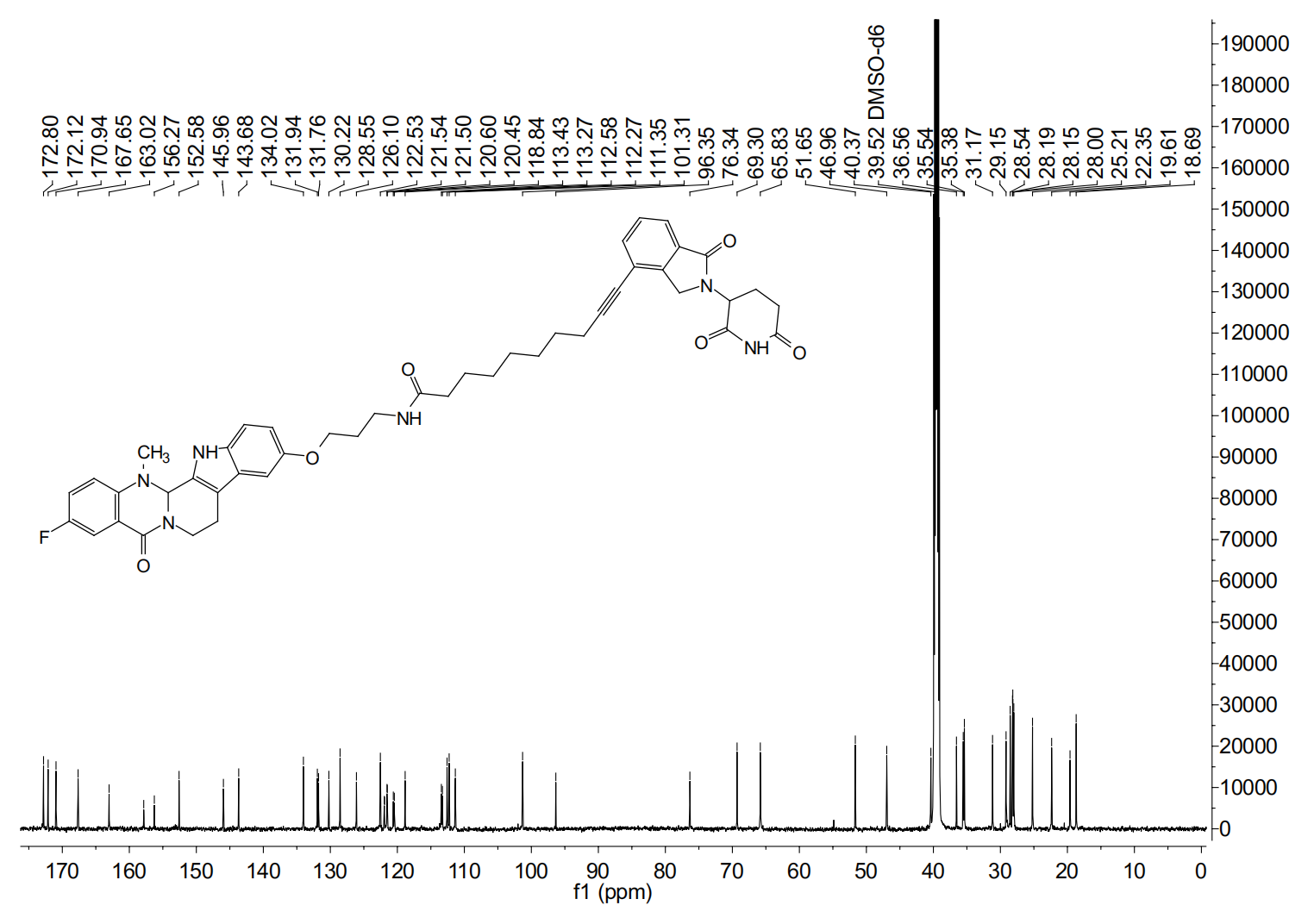


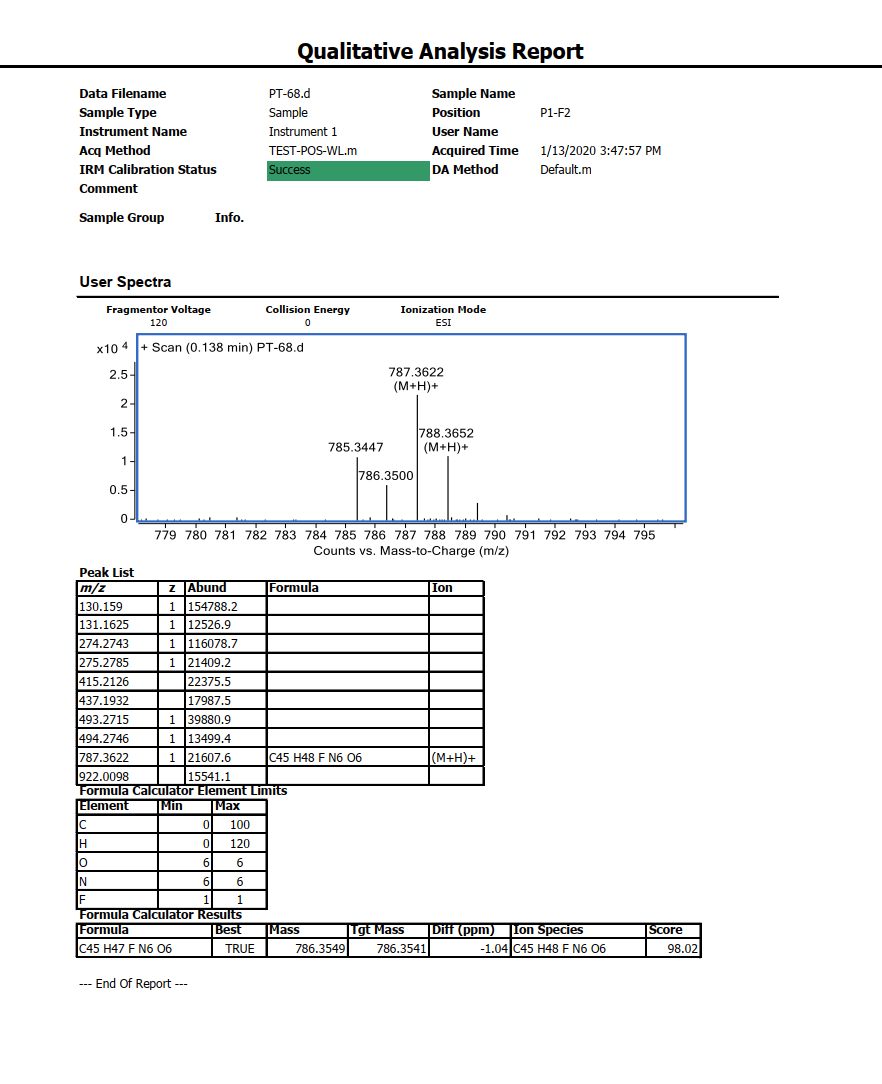


^1^H NMR (600 MHz) ^13^C NMR (150 MHz) and ESI-MS of compound **9a** in DMSO-*d_6_*


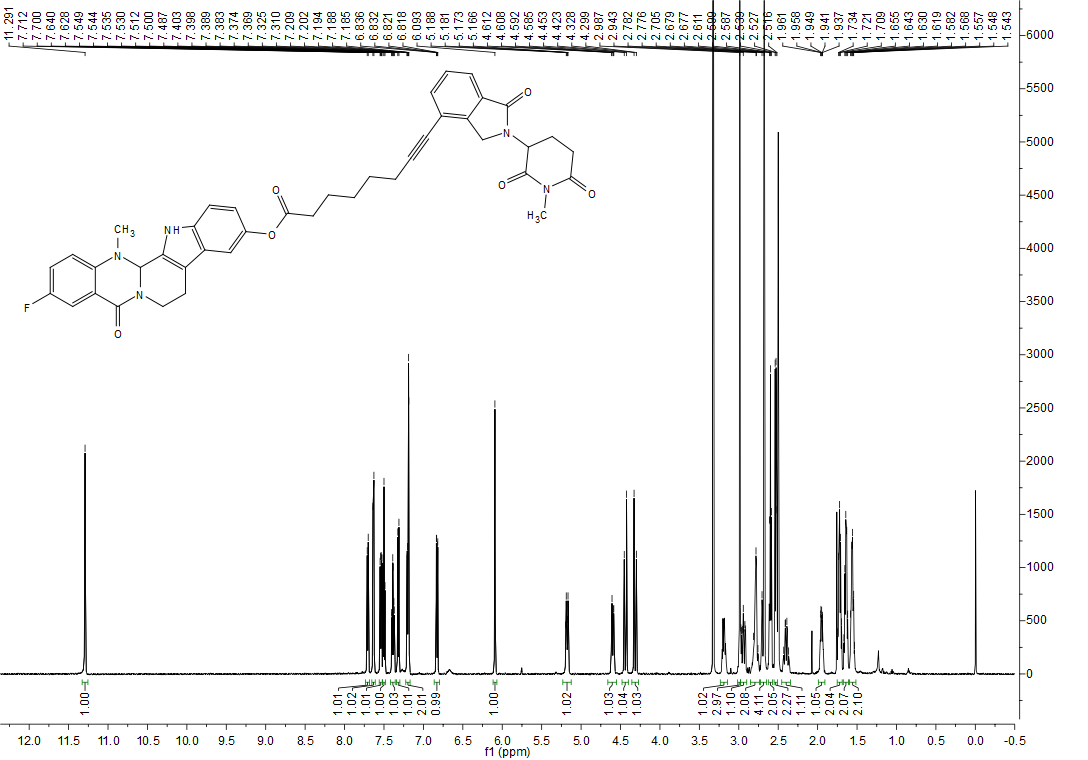


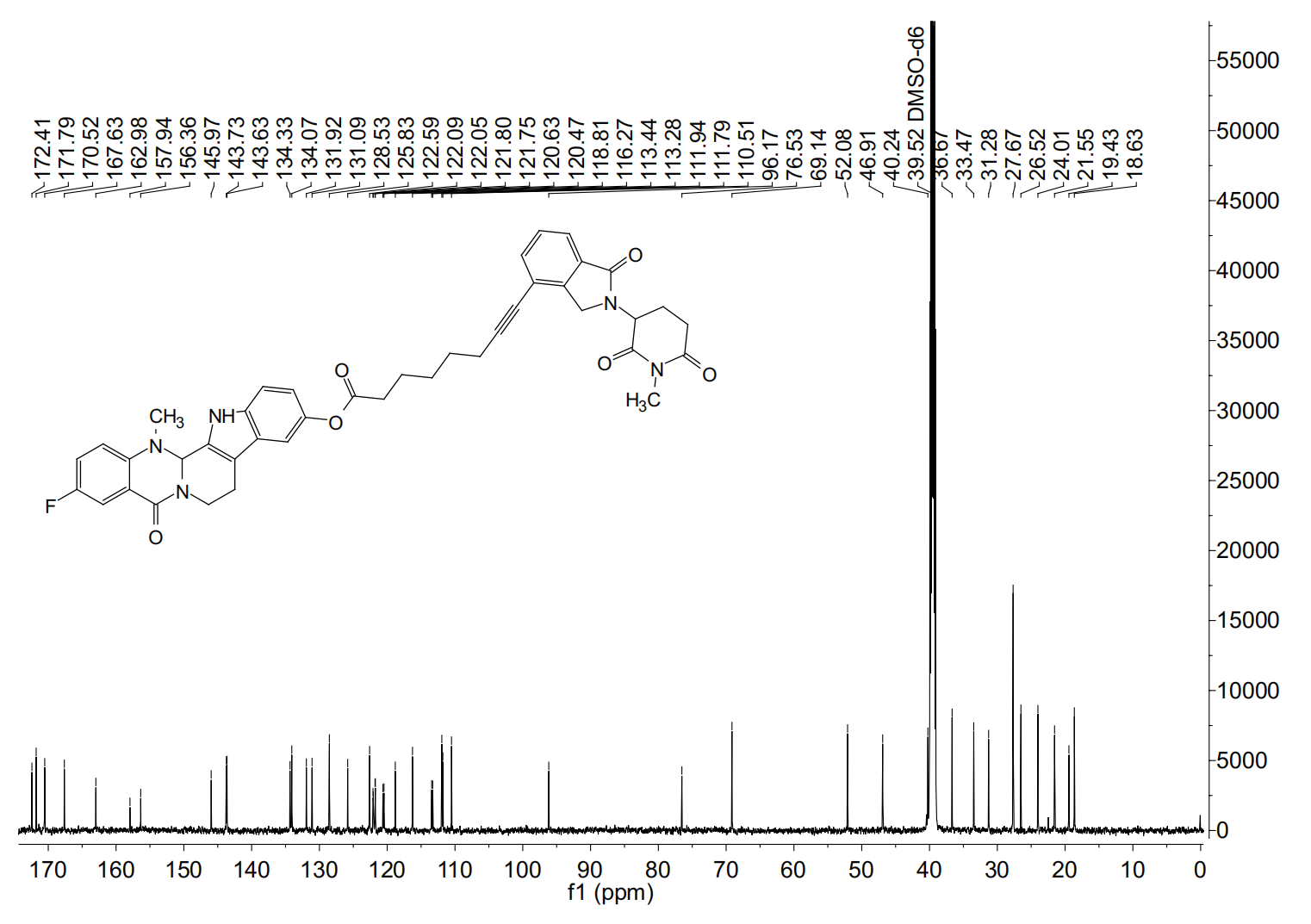


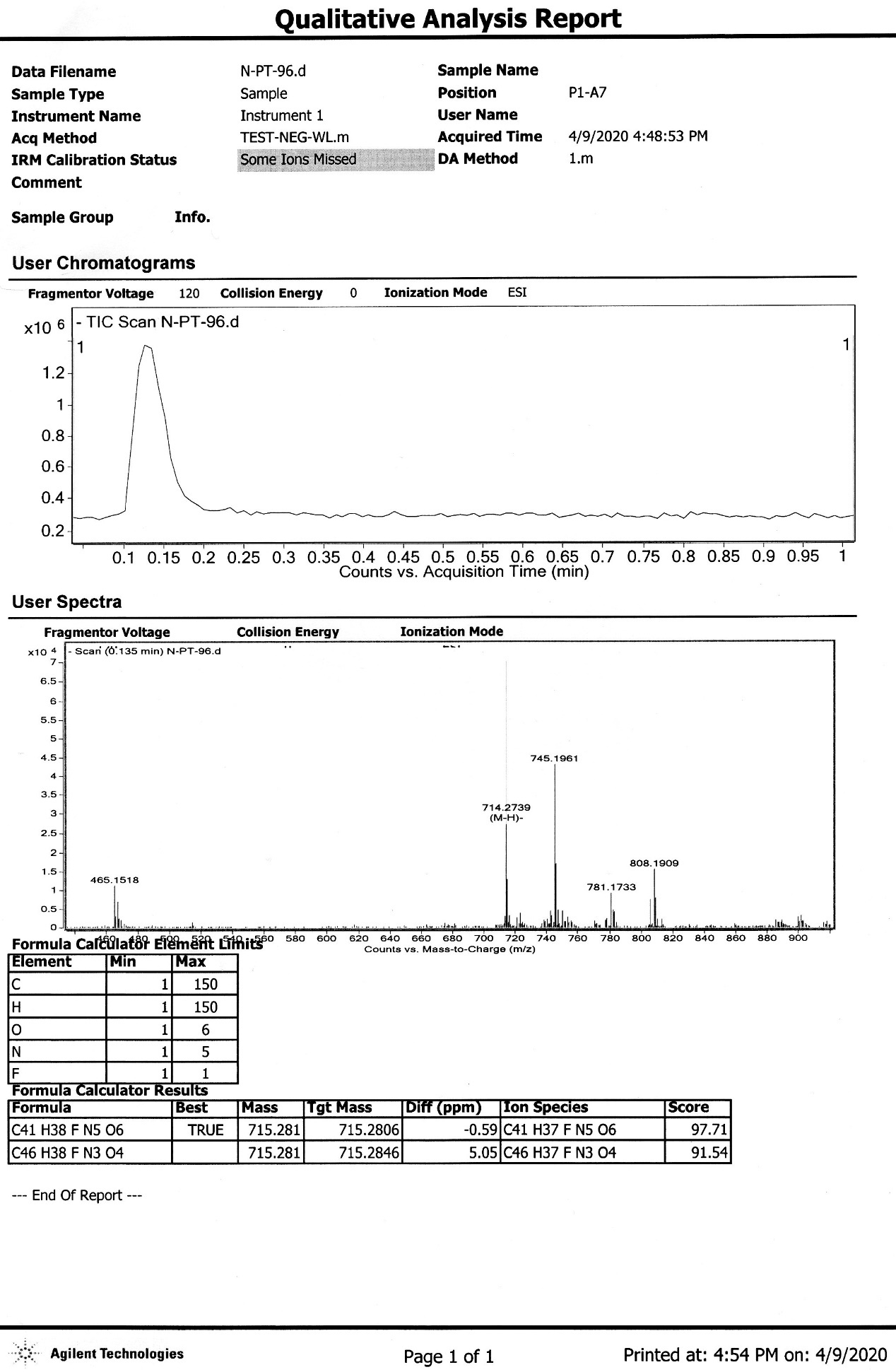


^1^H NMR (600 MHz) ^13^C NMR (150 MHz) and ESI-MS of compound **9b** in DMSO-*d_6_*


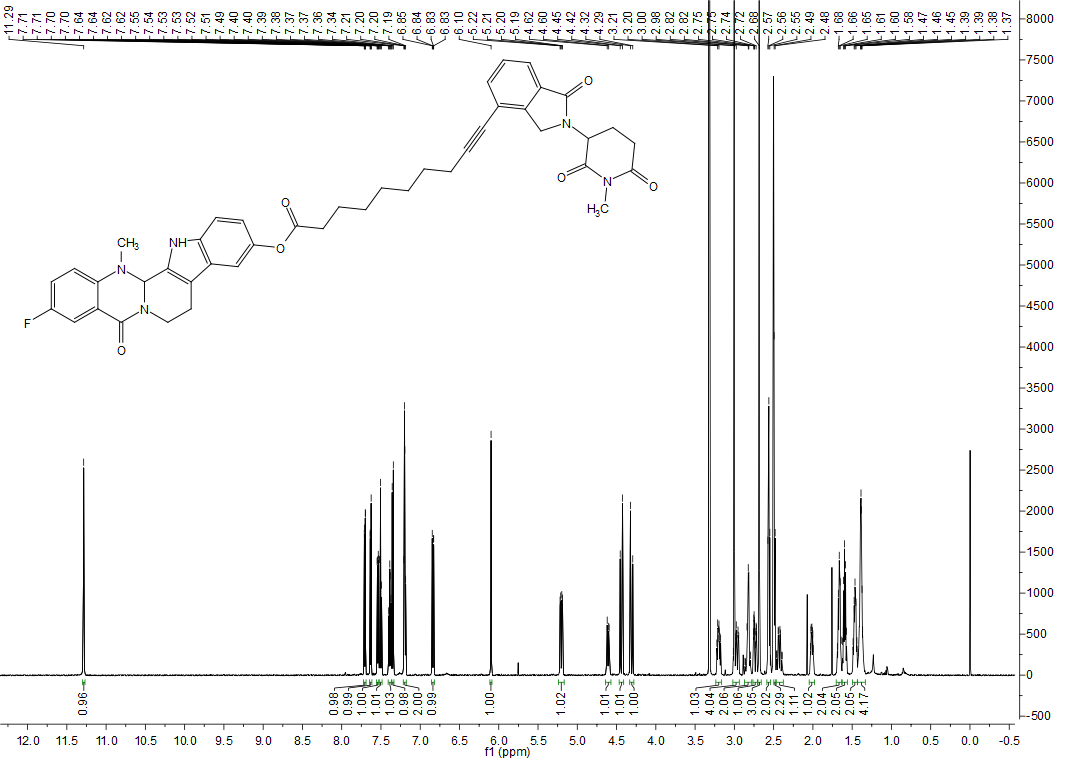


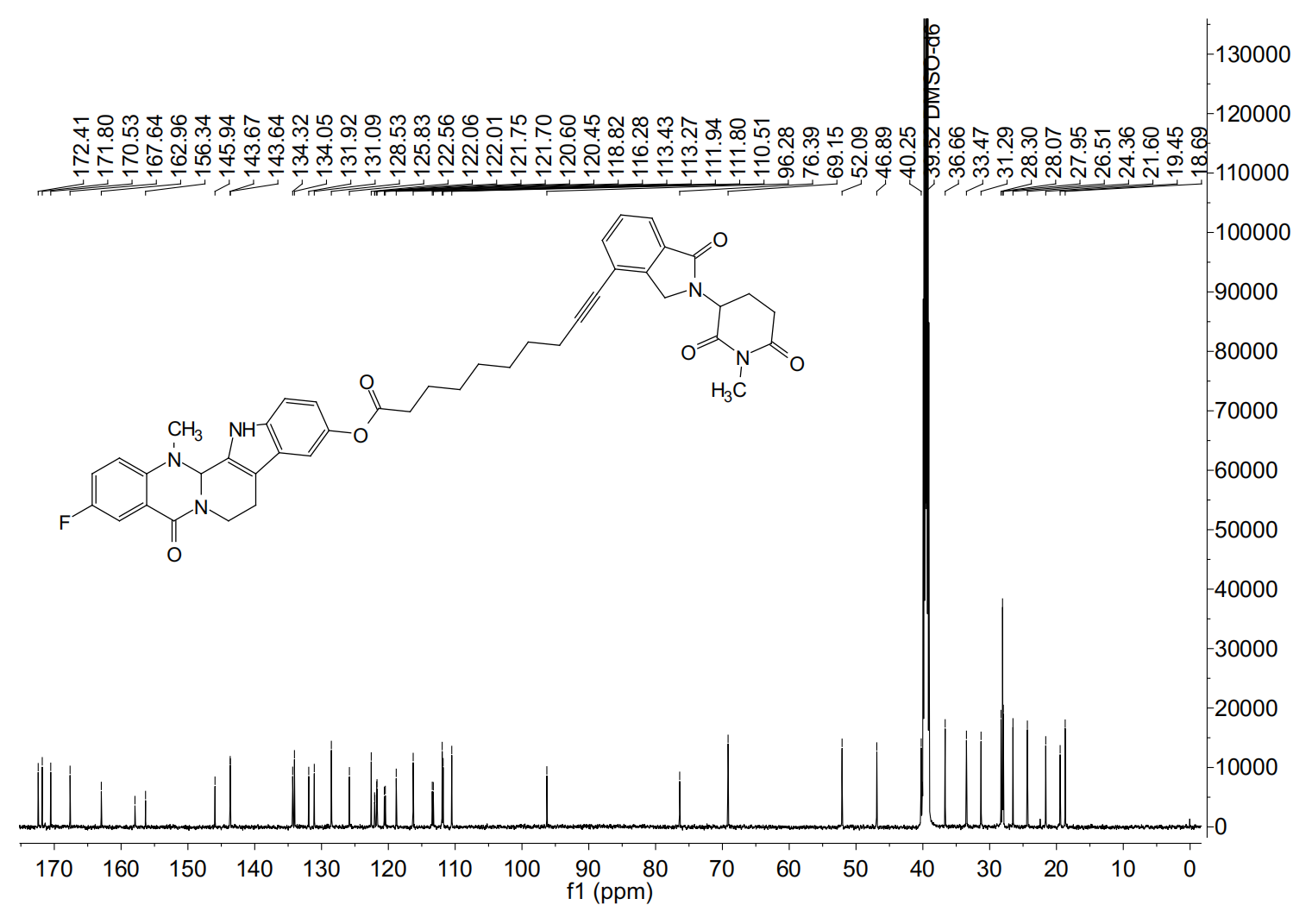


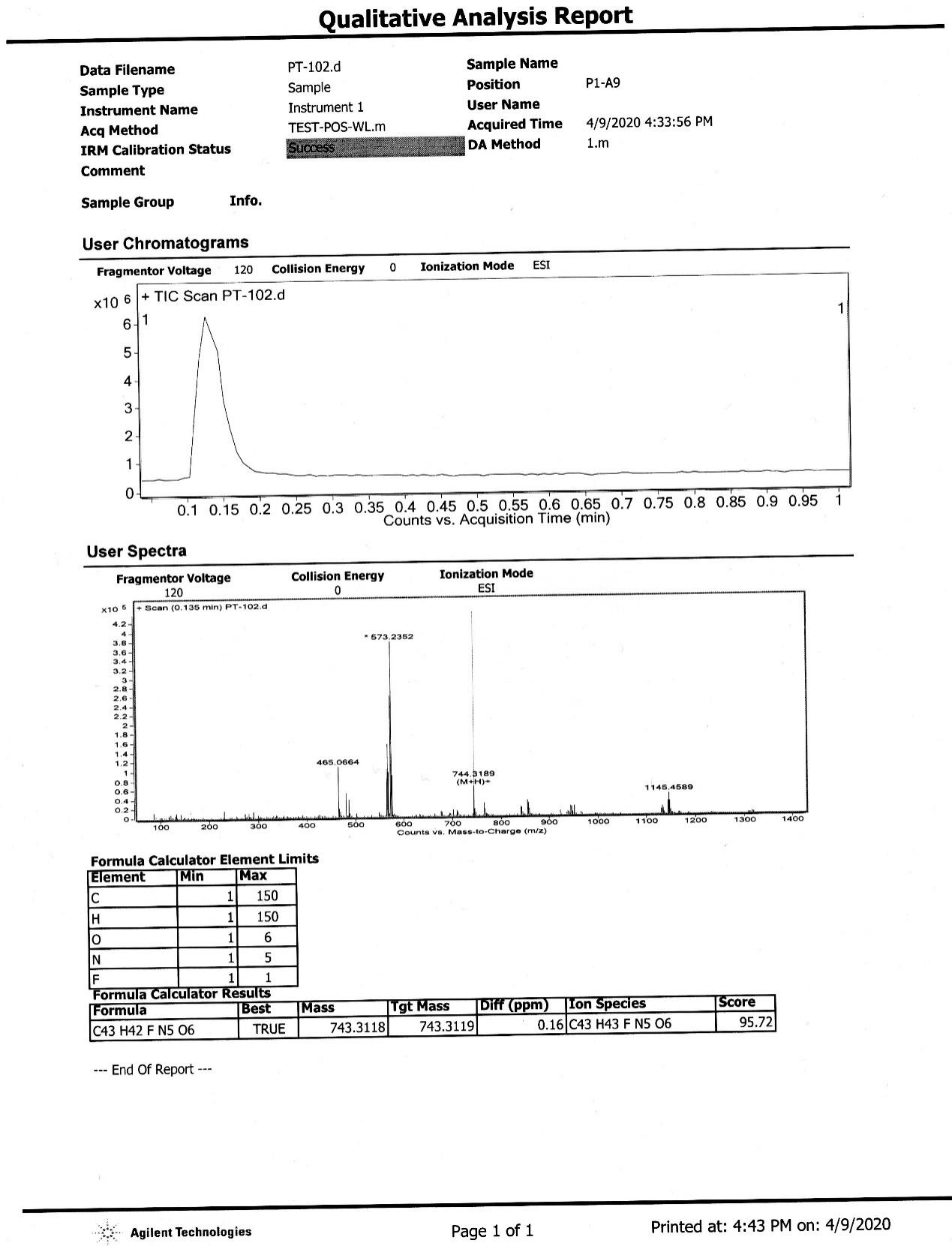


^1^H NMR (600 MHz) ^13^C NMR (150 MHz) and ESI-MS of compound **14a** in DMSO-*d_6_*


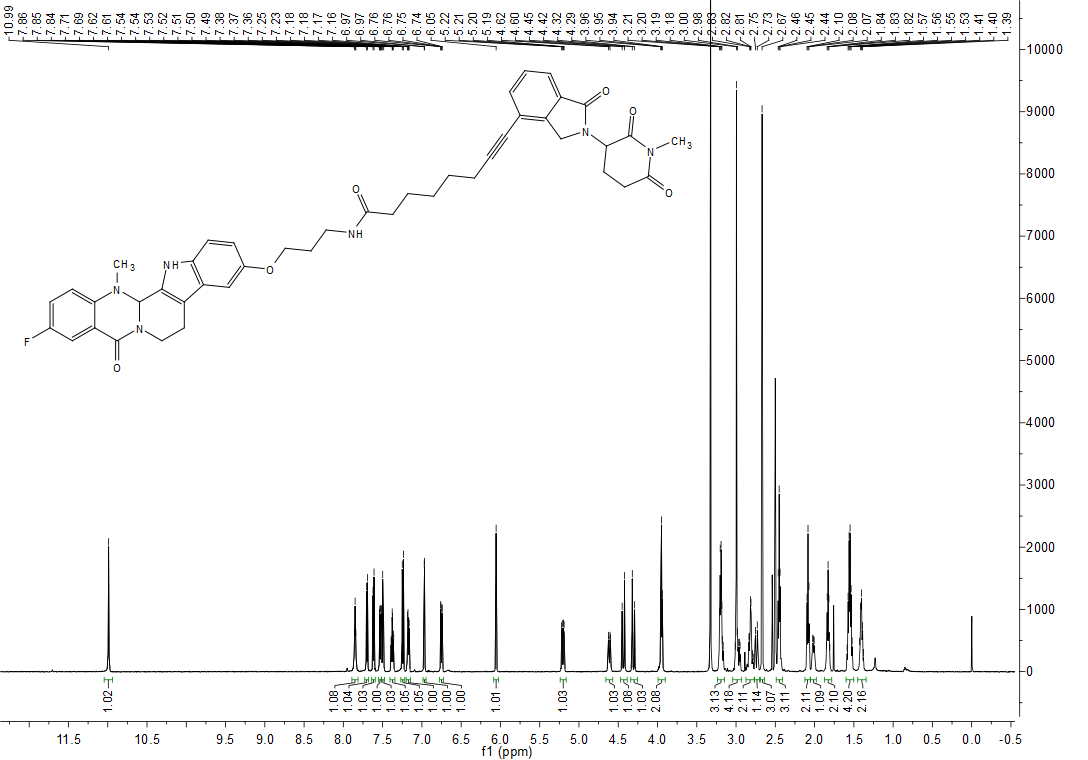


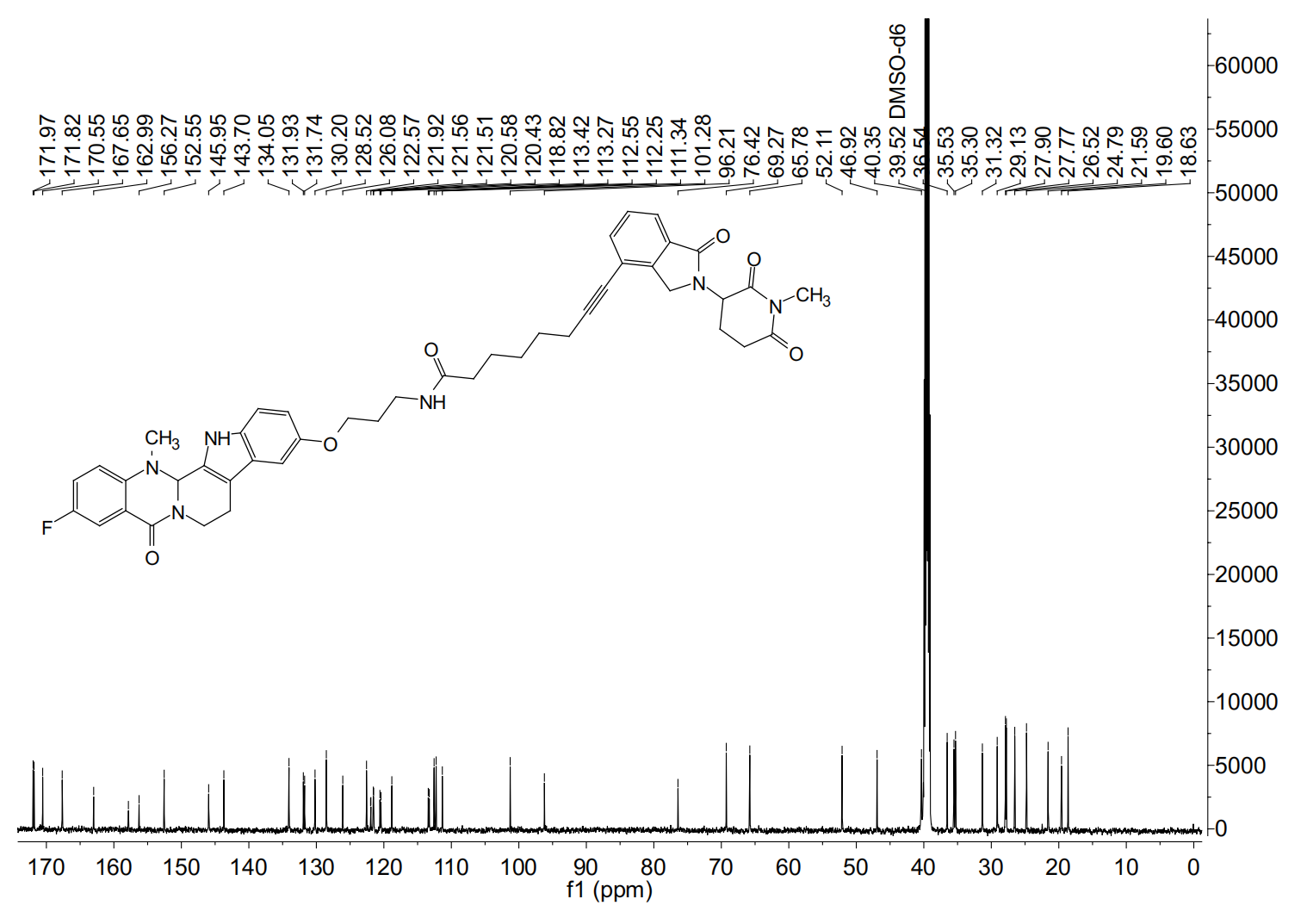


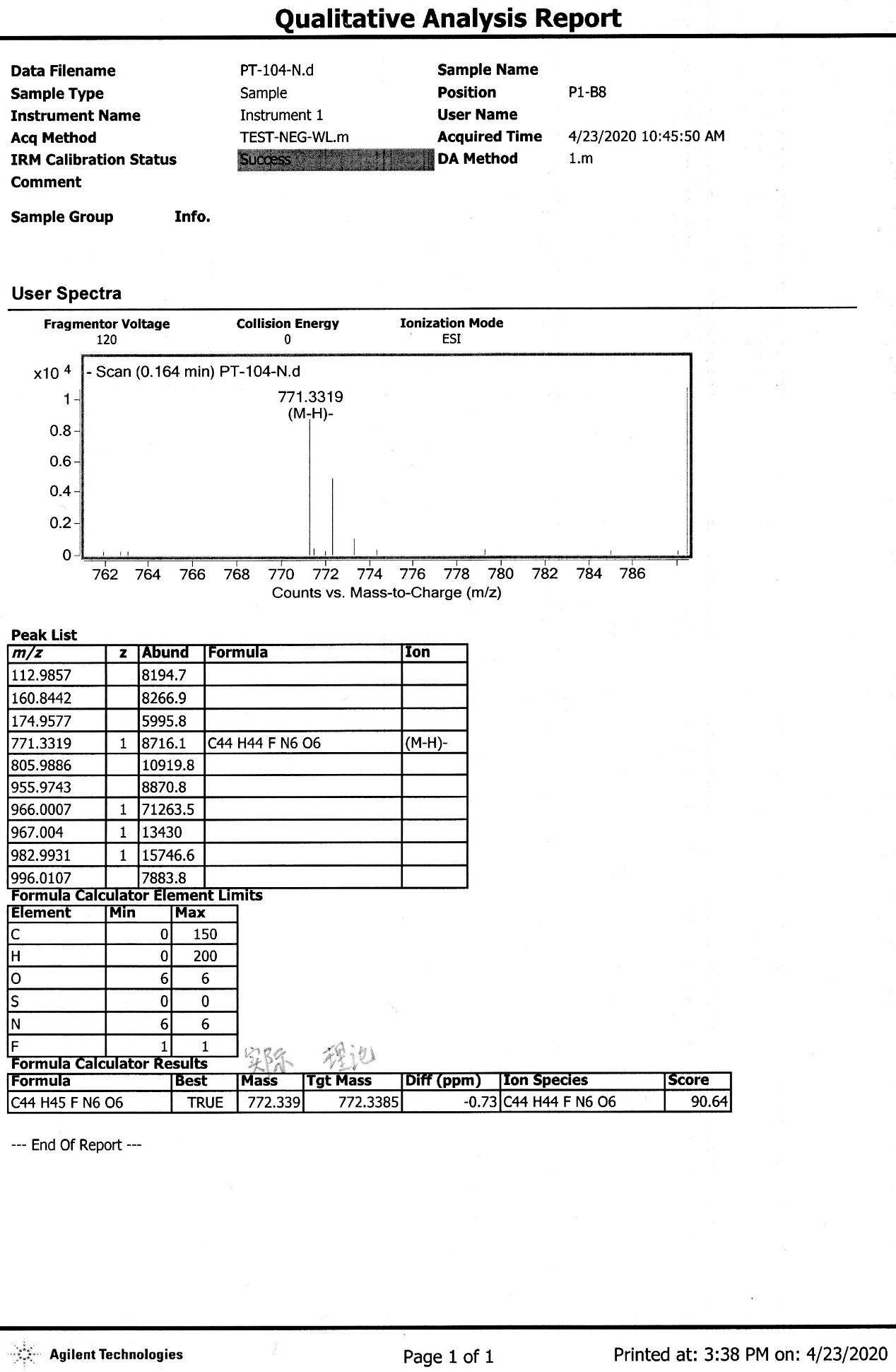


^1^H NMR (600 MHz) ^13^C NMR (150 MHz) and ESI-MS of compound **14b** in DMSO-*d_6_*


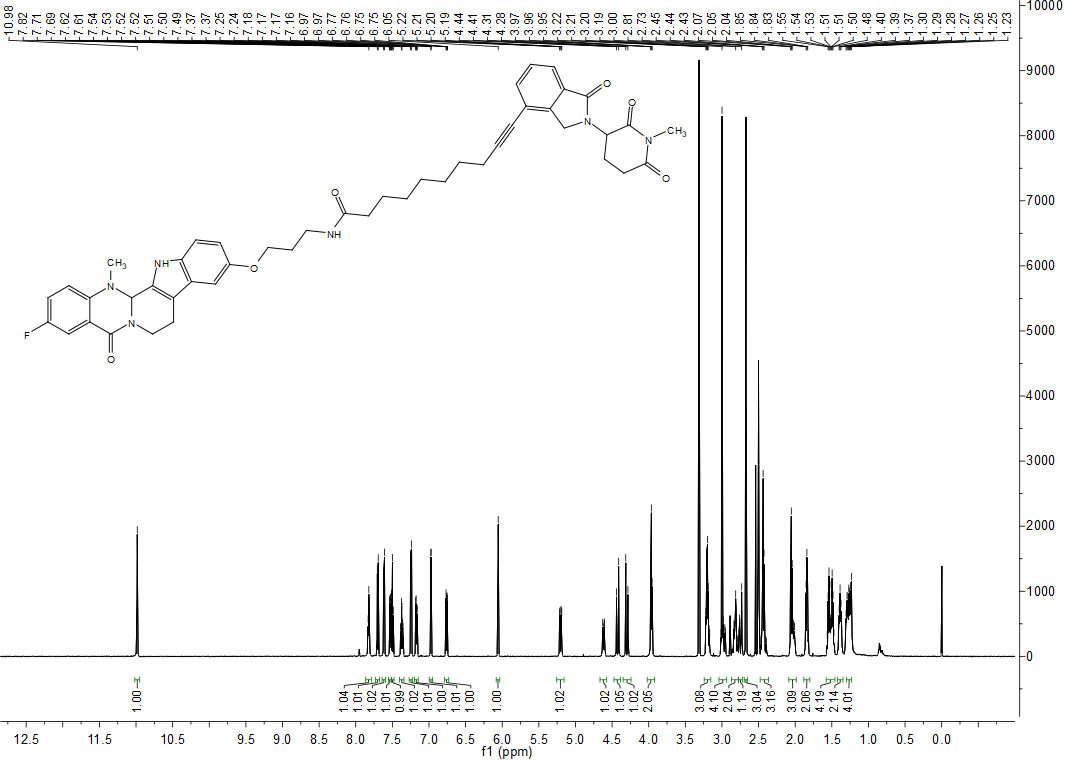


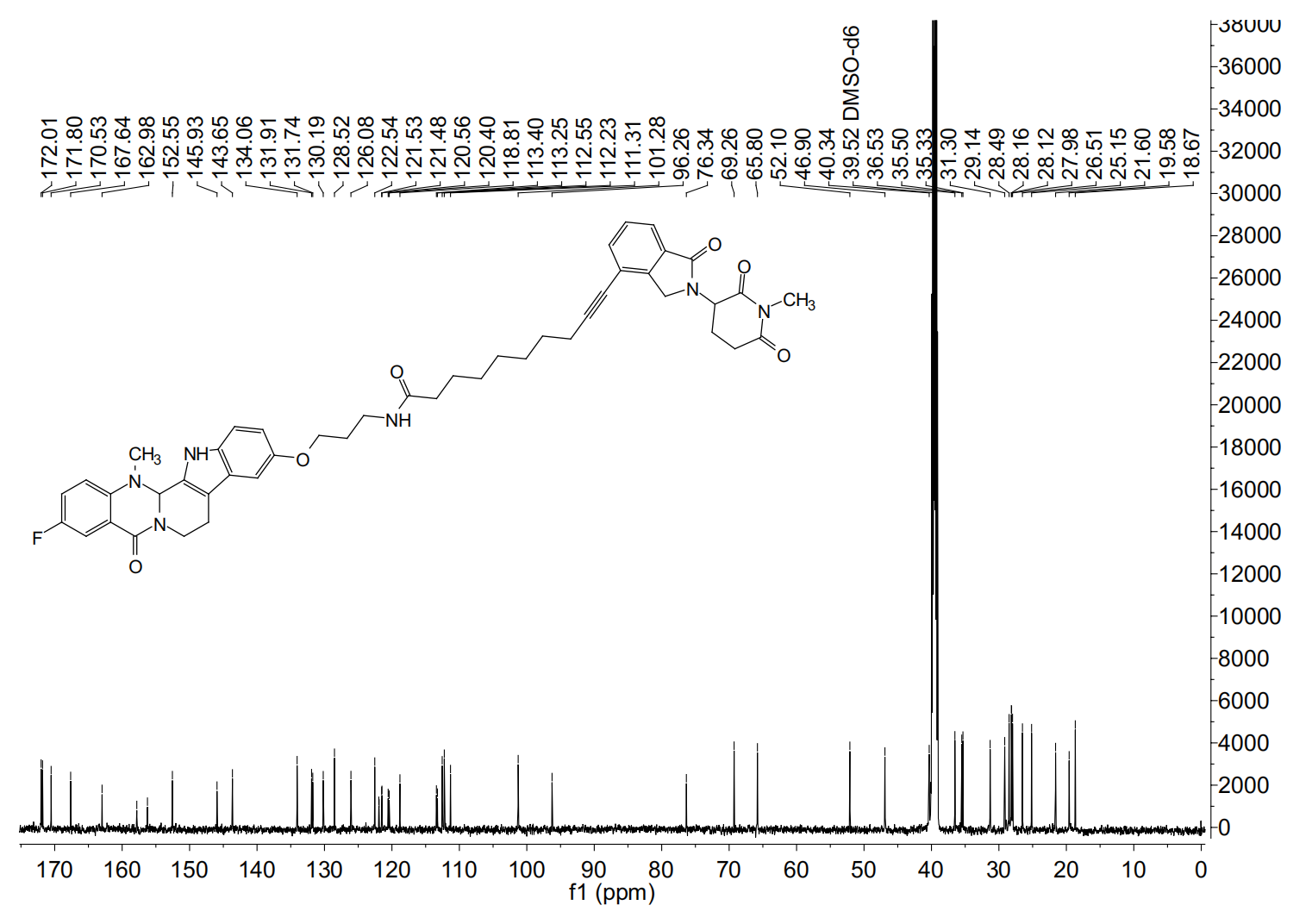


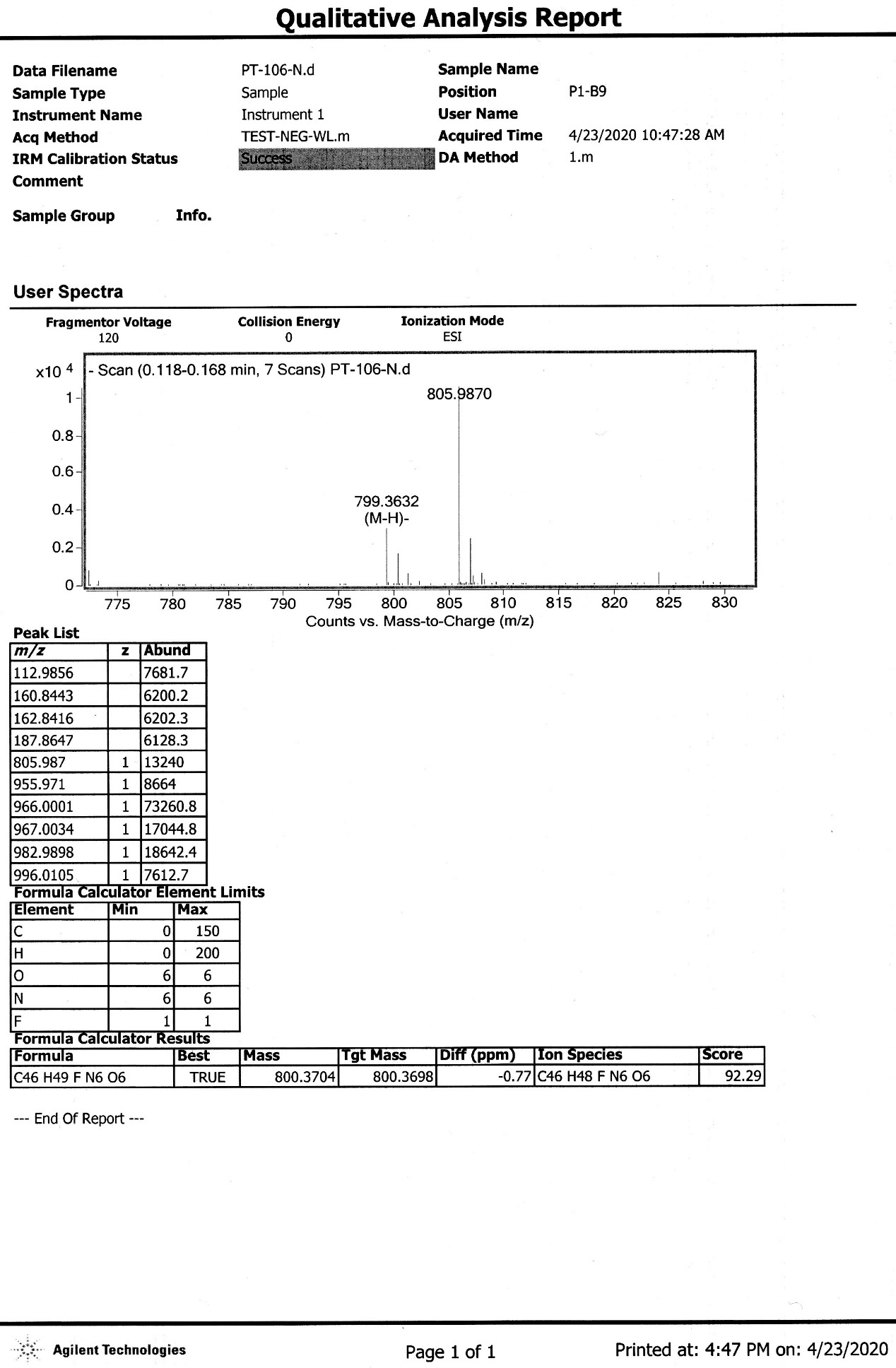


**Representative HPLC Purity of PROTACs**

**13c**


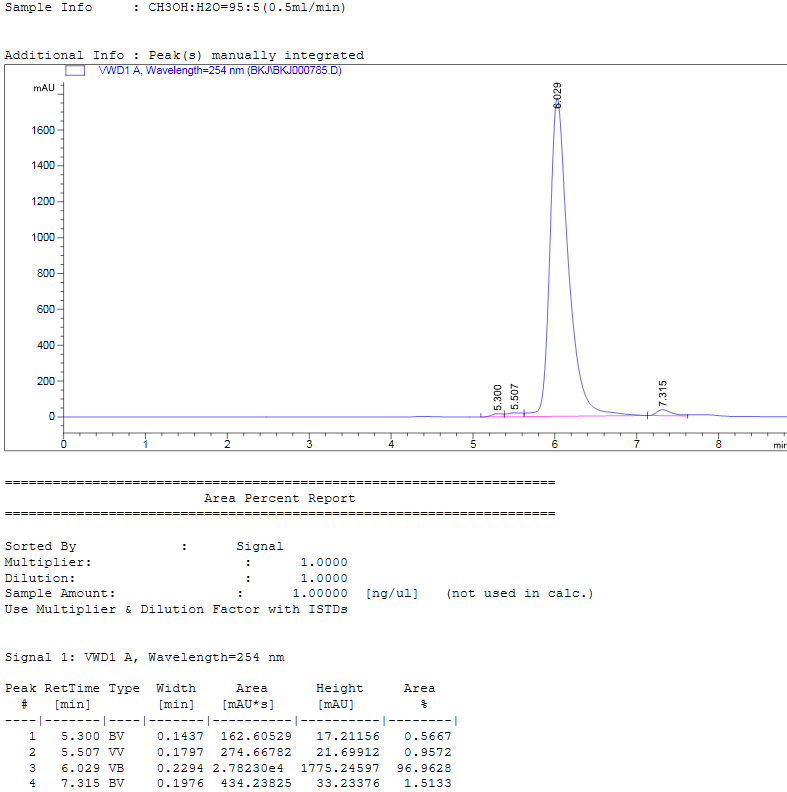


**13e**


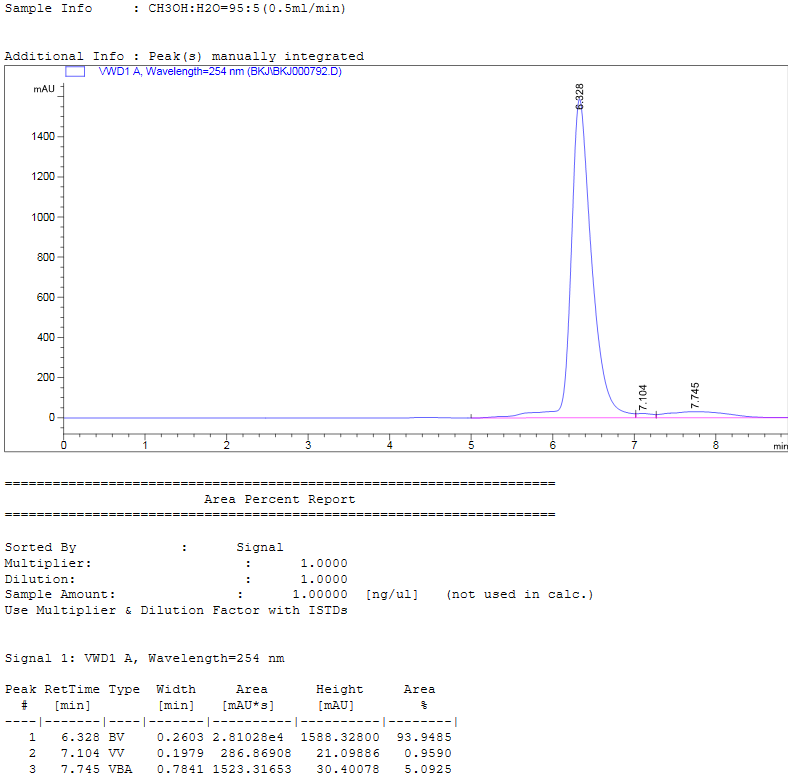


**14a**


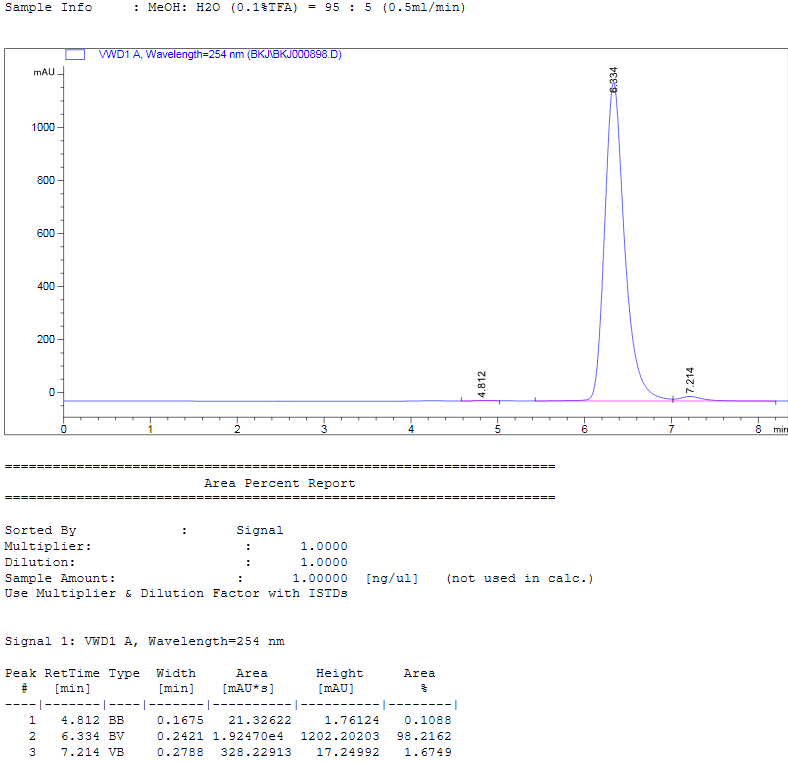


**14b**


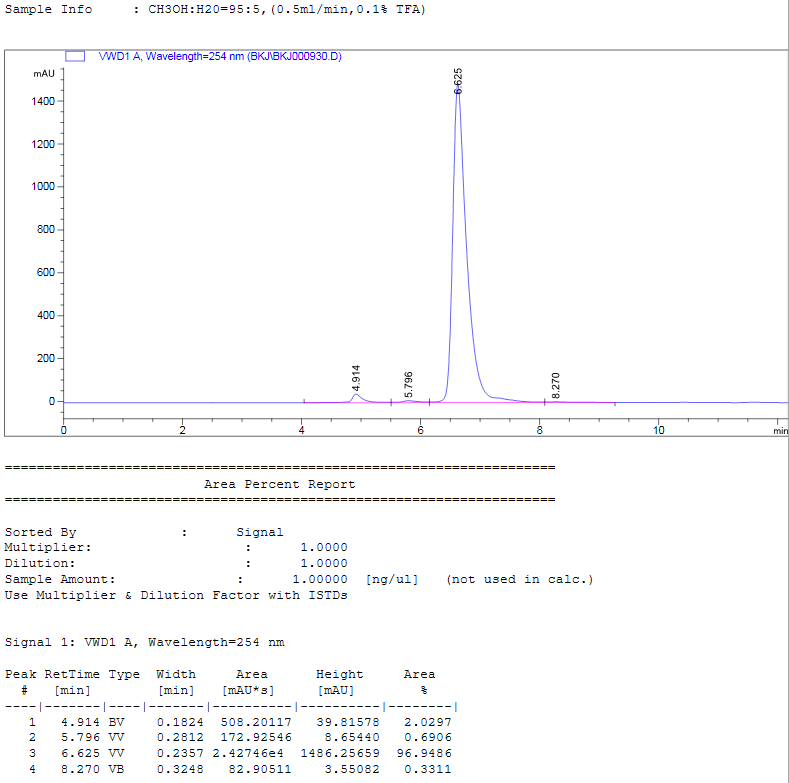


**The unprocessed images of western blot.**


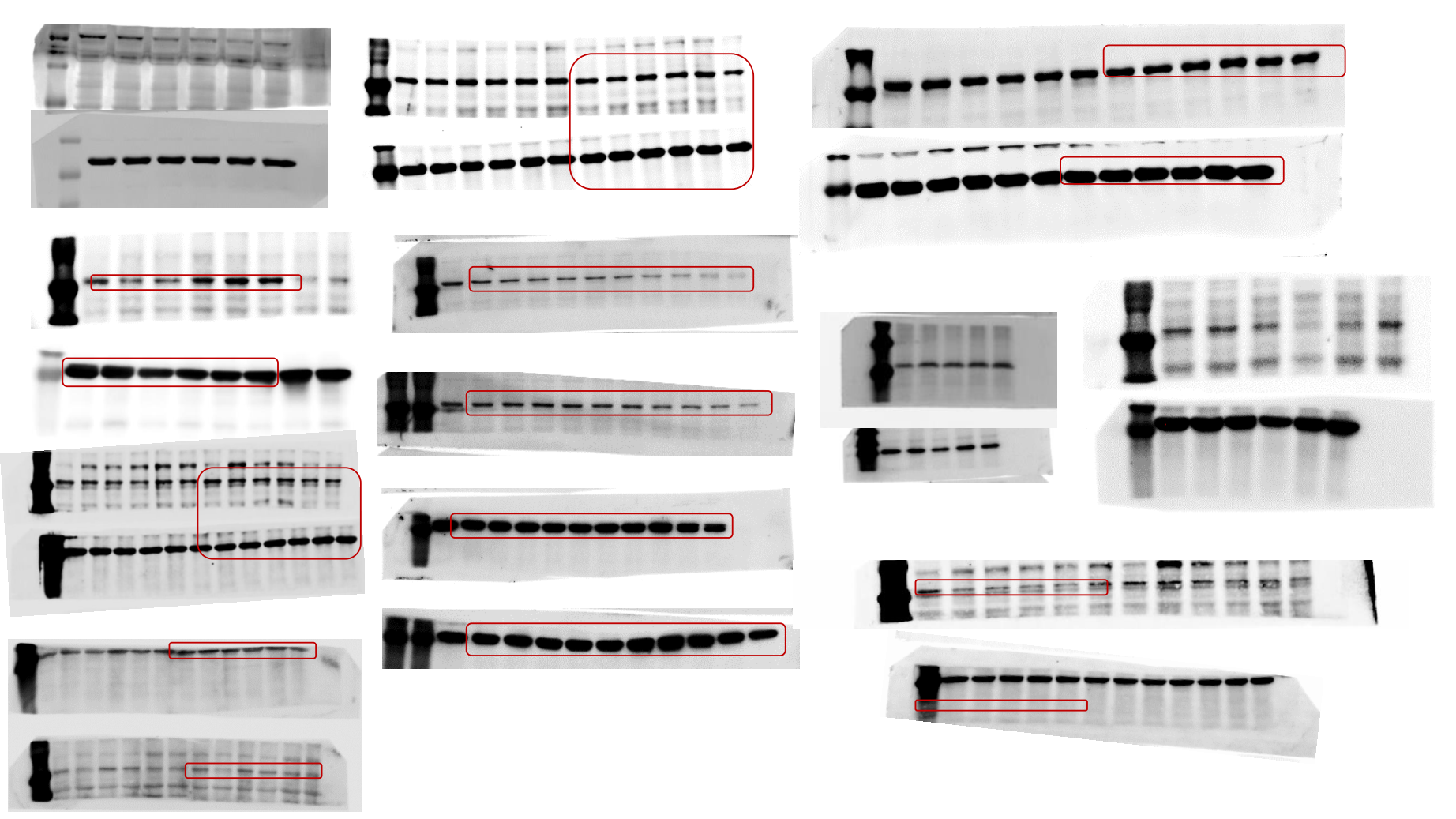


# References

[1] T. Xu, S. Zhang, G. Dong, X. Liu, X. Wang, X. Lv, et al. Discovery and characterization of novel small-molecule inhibitors targeting nicotinamide phosphoribosyltransferase. Sci. Rep. 5 (2015) 10043. DOI: 10.1038/srep10043.
